# Ambident reactivity of enolizable 5-mercapto-1*H*-tetrazoles in trapping reactions with in situ-generated thiocarbonyl *S*-methanides derived from sterically crowded cycloaliphatic thioketones

**DOI:** 10.3762/bjoc.21.113

**Published:** 2025-07-23

**Authors:** Grzegorz Mlostoń, Małgorzata Celeda, Marcin Palusiak, Heinz Heimgartner, Marta Denel-Bobrowska, Agnieszka B Olejniczak

**Affiliations:** 1 Department of Organic and Applied Chemistry, Faculty of Chemistry, University of Lodz, Tamka 12, 91-403 Łódź, Poland; 2 Department of Physical Chemistry, Faculty of Chemistry, University of Lodz, Pomorska 163/165, 90-236 Łódź, Poland; 3 Department of Chemistry, University of Zurich, Winterthurerstrasse 190, CH-8057 Zurich, Switzerland; 4 Institute of Medical Biology, Polish Academy of Sciences, 106 Lodowa St., 93-232 Łódź, Poland

**Keywords:** 2,5-dihydro-1,3,4-thiadiazoles, enolizable 5-mercapto-1*H*-tetrazoles, insertion reactions, thiiranes, thiocarbonyl ylides, X-ray analysis

## Abstract

The in situ-generated thiocarbonyl *S*-methanides (thiocarbonyl ylides), derived from cycloaliphatic thioketones, are efficiently trapped by enolizable 1-substitued 5-mercapto-1*H*-tetrazoles and formation of the corresponding N–H or S–H insertion products, i.e., thioaminals or dithioacetals, respectively, was observed. In some instances, both products were formed side by side and could be separated by chromatography. Two novel, sterically overcrowded bis-spiro(cyclopentyl) and bis-spiro(cyclohexyl)-substituted thiocarbonyl *S*-methanides were thermally generated from the corresponding 1,3,4-thiadiazolines and their reactivity towards 5-mercapto-1*H*-tetrazoles was compared with well-known analogues derived from adamantanethione and 2,2,4,4-tetramethyl-3-thioxocyclobutanone. Some of the isolated thioaminals were observed to undergo thermal isomerization in CDCl_3_ solution yielding the corresponding dithioacetals. Structural analysis of the isolated products of S–H and N–H insertion was carried out based on spectroscopic data (^1^H and ^13^C NMR) and the structures of two representatives were established by using the X-ray single crystal diffraction analysis method. Biological activity (cytotoxicity) of some selected products derived from 5-mercapto-1*H*-tetrazoles was also examined.

## Introduction

Cycloaddition reactions, including 1,3-dipolar cycloadditions, are considered as one of the most important types of organic reactions with key importance for the development of methods of modern organic synthesis [1–3]. Therefore, conversions of 1,3-dipoles which cover not only cycloadditions but also annulation and insertion reaction attract great attention worldwide [2–4]. Thiocarbonyl *S*-methanides (thiocarbonyl ylides) **1**, which were first reported in the 1980s by R. Huisgen and co-workers contributed to a substantial extension of mechanistic interpretations of cycloaddition reactions and to rapid development of methods applied not only for the synthesis of sulfur heterocycles but also for the synthesis of sulfur-containing organic compounds in general [2,5–6].

**Scheme 1:**
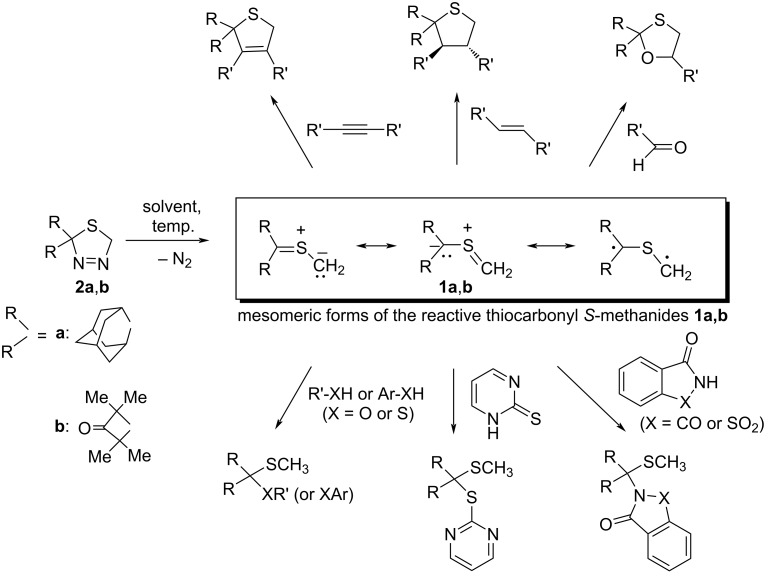
Typical [3 + 2] cycloaddition (above) and trapping (below) reactions of thiocarbonyl *S*-methanides **1a** and **1b** derived from cycloaliphatic thiones (adamantanethione (**7a**) and 3-thioxo-2,2,4,4-tetramethylcyclobutanone (**7b**), respectively).

Thiocarbonyl *S*-methanides **1** belong to the class of so-called *S*-centered, electron-rich 1,3-dipoles and in numerous studies, thermal decomposition of 1,3,4-thiadiazolines **2**, which are easily accessible via [3 + 2]-cycloaddition of respective thiocarbonyl dipolarophiles (preferably non-enolizable thioketones) with diazomethane, was demonstrated as a favorable method for their generation [5–6]. In the past three decades, 1,3-dipoles **1** have extensively been studied as useful building blocks for the preparation of various five-membered heterocycles via [3 + 2]-cycloaddition reactions. Depending on the type of the dipolarophile used in these reactions, the target heterocycles may contain only one sulfur atom (cycloadditions with C=C or C≡C dipolarophiles) or more heteroatoms (cycloadditions with C=S, C=O, C=N, N=N, S=O, N=S=O, etc. dipolarophiles) [5–10] (Scheme 1, upper part).

Notably, [3 + 2]-cycloadditions of the sterically crowded thiocarbonyl *S*-methanide **1b** with electron deficient, tetrasubstituted ethylenes activated with CN and CF_3_ (or CN and CO_2_Me) groups were encountered as the first examples of non-concerted, step wise cycloadditions leading to mixtures of five- and seven-membered heterocyclic products (cycloadducts), and respective zwitterionic intermediates were postulated to explain the unexpected reaction pathway [11].

Due to the practically equal electronegativity of carbon and sulfur (χ = 2.55 (for C) and χ = 2.58 (for S) according to the Pauling scale), thiocarbonyl *S*-methanides **1** are considered as electron-rich 1,3-dipoles with basic and nucleophilic reactivity displayed by the =S^+^–CH_2_^‒^ unit [6]. Therefore, acidic compounds of type R–XH (X = NR’, O, S), which are able to protonate this basic fragment, undergo 1,3-addition leading to corresponding products of *S*,*S-*, *O*,*S-*, or *N*,*S*-acetal type. For example, trapping of the in situ-generated adamantanethione *S*-methanide (**1a**) with *tert*-butylthiol or benzyl alcohol leads to the corresponding *S*,*S*-dithioacetal and *O*,*S*-thioacetal, respectively [2,6]. On the other hand, enolizable imidazole-2-thiones and pyrimidine-2-thione were reported to react with **1a** and **1b** yielding *S*,*S*-dithioacetals as exclusive trapping/insertion products [12] (Scheme 1). Notably, enolizable nitrogen heterocycles such as imidazole and pyrazole, or bicyclic heterocycles like saccharin and phthalimide, reacted with **1a** and **1b** yielding the corresponding N–H insertion products [13–14] (Scheme 1, lower part).

**Scheme 2:**
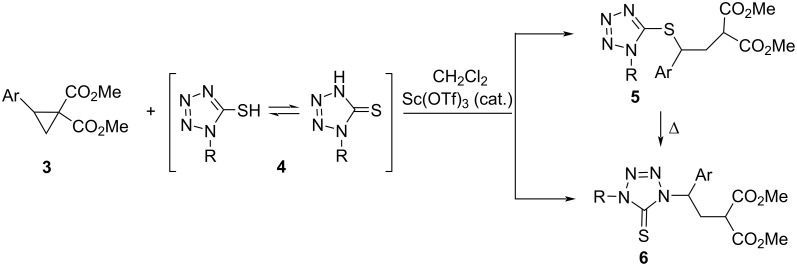
Ambident reactivity of 5-mercapto-1*H*-tetrazoles **4** towards dimethyl 2-arylcyclopropane dicarboxylates **3**; formation of the S- and N-insertion products **5** and **6**, respectively [15].

In a recent publication, ring opening reactions of so-called D–A cyclopropanes (dimethyl 2-arylcyclopropane-1,1-dicarboxylates **3**), initiated by a nucleophilic attack of 1-substituted 5-mercapto-1*H*-tetrazoles **4** [15] and other structurally similar enolizable mercapto azaheterocycles [16], leading to the S- and N-insertion products of type **5** and **6**, respectively, were described (Scheme 2). Remarkably, in this study, the influence of the Ar substituent on the chemoselectivity was observed.

A detailed study on the mechanism of these reactions showed that the initially formed S–H insertion products (type **5**) underwent thermal isomerization leading to thermodynamically more stable N–H insertion ones (type **6**) (Scheme 2) and the isomerization process was clearly dependent on the substitution pattern of both substrates, i.e., substituent Ar in the starting cyclopropane **3** and the R–N(1) moiety in the tetrazole derivative **4** [15].

Taking into account the known and widely documented importance of enolizable 5-mercapto-1*H*-tetrazoles as important bioisosters [17–21] we decided to study selected heterocycles of this type in reactions with thiocarbonyl *S*-methanides **1a**–**d**, derived from cycloaliphatic thioketones, which belong to the class of so called ‘sulfur centered 1,3-dipoles’ [6]. Thus, the goal of the present study was the examination of the behavior of the in situ-generated **1** towards enolizable 5-mercapto-1*H*-tetrazoles **4** bearing various aliphatic and aromatic substituents at the N(1) atom. Competition between the expected S–H and N–H insertion processes was of primary interest. In extension of the synthetically oriented study, bioactivity of selected products formed via S–H and N–H insertion reactions should also be tested. This method of modification of the structure of enolizable 5-mercapto-1*H*-tetrazole has not yet been described.

## Results and Discussion

### New precursors **2c,d** of sterically crowded thiocarbonyl *S*-methanides **1c,d**

**Scheme 3:**
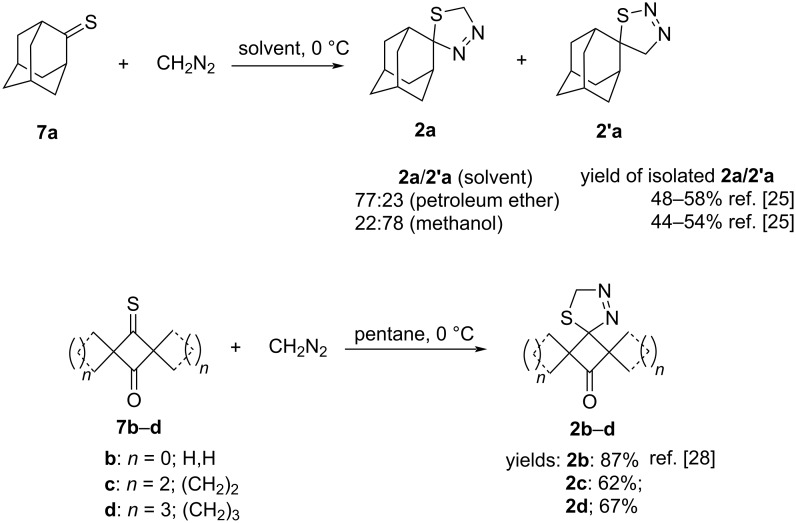
Regioselectivity of [3 + 2] cycloadditions of diazomethane with adamantanethione (**7a**) [22,24–25], and sterically crowded thioketones **7b** [28], as well as **7c**,**d** derived from cyclobutanedione.

Based on the well-established methodology, transient thiocarbonyl *S*-methanides **1** should be generated in situ by thermal decomposition of their precursors, i.e., spiro-1,3,4-thiadiazolines **2** [5–6]. In contrast to adamantanethione (**7a**), which reacts with diazomethane (CH_2_N_2_) yielding a mixture of regioisomeric 1,3,4- and 1,2,3-thiadiazoline **2a** and **2’a**, respectively [22], cyclobutanethione **7b** undergoes the same [3 + 2]-cycloaddition to give crystalline 1,3,4-thiadiazoline **2b** as the sole product (Scheme 3) [23]. In the case of **7a** the ratio of cycloadducts **2a** and **2’a** strongly depends on the type of the solvent used in the experiment and a striking difference observed in the reactions performed in petroleum ether and in methanol is presented in Scheme 3 [22]. This amazing effect was studied in detail and discussed in later publications by R. Huisgen et al. [24–25]. Two dispiro-thioketones **7c** and **7d**, which are also known [26–27], were analogously treated with CH_2_N_2_ at ca. 0 °C and the expected cycloadducts **2c** and **2d** were formed with complete regioselectivity, and subsequently could be isolated in good yields without a remarkable decomposition (Scheme 3).

**Scheme 4:**
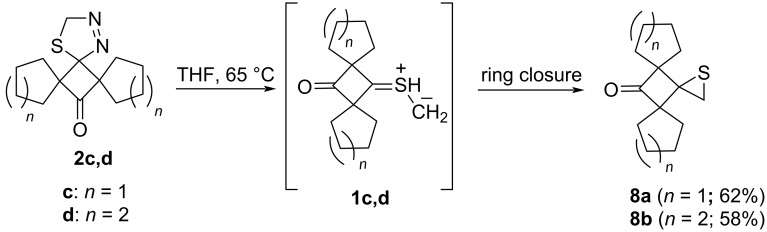
The in situ generation of sterically crowded thiocarbonyl *S*-methanides **1c**,**d** (via a 1,3-dipolar cycloreversion) in absence of any trapping reagent and their electrocyclization (ring closure) leading to thiiranes **8a**,**b**.

Thermal decomposition of **2a** and **2b**, and the behavior of the corresponding thiocarbonyl *S*-methanides **1a** and **1b**, respectively, in situ generated at 45 °C in absence of any trapping reagent, were described in earlier publications [6,10–14,28]. In the present study, the thermal stability of the new dispiro-1,3,4-thiadiazolines **2c** and **2d** was tested in THF solution at a slightly higher temperature (60 °C). In both cases, evolution of nitrogen was completed after ca. 3 h and pure thiiranes **8a** and **8b** were isolated as sole products in 62%, and 58% yield, respectively (Scheme 4). Thus, their formation clearly evidenced formation of the respective thiocarbonyl *S*-methanides **1c**,**d**, respectively, as the expected reactive intermediates after N_2_ elimination from **2c** and **2d**.

### Trapping of transient thiocarbonyl *S*-methanides **1** with enolizable 1-substituted 5-mercapto-1*H*-tetrazoles **4**

Adamantanethione *S*-methanide (**1a**) is considered as a prominent representative of the ‘sulfur-centered 1,3-dipoles’ and its chemistry has been reported in numerous publications [6–7,10–14]. Therefore, it was selected to perform a test trapping-experiment with enolizable 5-mercapto-1-methyl-1*H*-tetrazole (**4a**). The reaction with nearly equimolar amounts of 1,3,4-thiadiazoline **2a** and **4a** was typically carried out at 45 °C in THF solution, and evolution of N_2_ was monitored using a nitrometer connected with the reaction flask. When the evolution of the gas was completed, the solvent was evaporated, and the crude residue was checked by means of ^1^H NMR. Two high-field shifted singlets, attributed to the Me‒S and Me‒N moieties were found at 1.86 and 3.86 ppm, respectively, and they were accompanied by two multiplets, attributed to two CH units of the adamantane ring, found at 2.37‒2.44 and 2.47‒2.54 ppm. To our delight, simple fractional crystallization from hexane with small admixture of CH_2_Cl_2_ led to isolation of a colorless, crystalline material with a narrow melting point of 107‒109 °C (yield 66%). The ^1^H NMR spectrum registered for the purified product perfectly fitted with that one of the crude mixture and thereby confirmed formation of a single product with a single set of the Me‒S and Me‒N groups located at the above reported shifts.

**Scheme 5:**
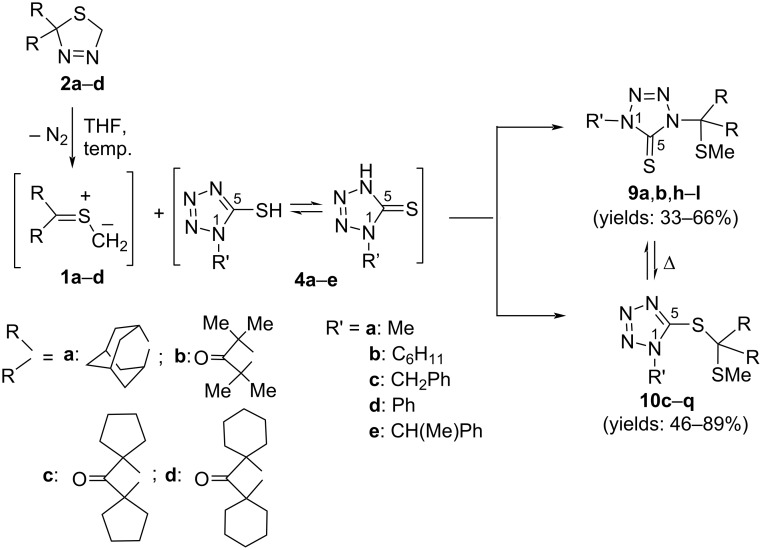
Reactions of the in situ-generated thiocarbonyl *S*-methanides **1** (from 1,3,4-thiadiazolines **2**) with enolizable 5-mercapto-1*H*-tetrazoles **4**, leading to the N- or S-insertion products **9** and **10**, respectively.

In the ^13^C NMR spectrum their signals were found in typical regions at 33.5 (N*C*H_3_) and 10.3 (S*C*H_3_) ppm, respectively. A characteristic signal of the –N*C*S− atom of the thioaminale moiety, located in the Ad-skeleton was found at 81.2 ppm. Finally, the signal located at 162.9 ppm was attributed to the C=S unit (thiourea type) and the chemical shift corresponded in this case very well with the respective data found for the N-insertion products of the reaction of **4a** with dimethyl 2-phenylcyclopropane-1,1-dicarboxylate [11]. In addition, elemental analysis confirmed the molecular formula C_17_H_20_N_4_S_2_ corresponding to an anticipated 1:1 insertion product. Based on all these data, the isolated product was identified as thioaminal **9a** formed with complete chemoselectivity from the in situ-generated **1a** and **4a** (Scheme 5, Table 1).

**Table 1:** Products of the reactions between thiocarbonyl *S*-methanides **1a**‒**d** and 5-mercapto-1*H*-tetrazoles **4a**–**e**.

Precursor **2**	5-Mercapto- 1*H*-tetrazole **4**	Thioaminals **9** (yield %)^a^	Dithioacetals **10** (yield %)^a^

**a**	**a**	**a** (66)	**a** ^b^
**a**	**b**	**b** (52)	**b** ^b^
**b**	**a**	**c** ^b^	**c** (84)
**b**	**b**	**d** ^b^	**d** (76)
**b**	**c**	**e** ^b^	**e** (72)
**b**	**d**	**f** ^b^	**f** (82)
**b**	**e**	**g** ^b^	**g** (46)
**c**	**a**	**h** (36)	**h** (47)
**c**	**b**	**i** (48)	**i** (49)
**c**	**c**	**j** (46)	**j** (29)
**c**	**d**	**k** (33)	**k** (63)
**c**	**e**	**l** (45)	**l** (50)
**d**	**a**	**m** ^b^	**m** (46)
**d**	**b**	**n** ^b^	**n** (72)
**d**	**c**	**o** ^b^	**o** (52)
**d**	**d**	**p** ^b^	**p** (89)
**d**	**e**	**q** ^b^	**q** (77)

^a^Calculated for isolated compounds **9** and **10**; ^b^not found in the crude reaction mixture.

Notably, the same chemoselectivity was observed in reactions of **1a** with 5-mercaptotetrazole **4b**. However, due to a lower reactivity observed for **4c**,**d** towards **1a**, the experiments with these derivatives were unsuccessful and formation of undesired side products was observed in both cases.

Unexpectedly, a different chemoselectivity was observed in reactions with **1b**. For example, thermal decomposition of **2b** performed in THF solution at 45 °C in the presence of **4a** (molar ratio 1.1:1.0) led to the exclusive formation of the S-insertion product, i.e., dithioacetal **10c**, which was isolated after chromatographic workup as a crystalline compound (mp 136‒138 °C) in high yield of 86%. The ^13^C NMR spectrum registered for this compound revealed a shift of the S‒*C*‒S atom, incorporated into the cyclobutanone ring, at 74.8 ppm. On the other hand, the thioaminal functionality with N‒*C*‒S moiety located within the tetrazole ring, shows a signal at 151.3 ppm. All products of type **10** in this series were obtained as crystalline materials in satisfactory to high yields (46‒86%) (Scheme 5, Table 1).

In contrast to **1a** and **1b**, trapping reactions of the sterically overcrowded *S*-methanide **1c** with 5-mercaptotetrazoles **4** occurred with no remarkable selectivity, and in all cases formation of insertion products of both types, i. e., thioaminals **9** and dithioacetales **10**, was observed (see Table 1). Chromatographic separation enabled the isolation of pure compounds, and the more stable dithioacetals **10** formed the more polar fraction. The less polar fraction contained thioaminals **9**, which underwent a slow isomerization in CDCl_3_ solution. Notably, in the case of products **9k** and **10k**, bearing a Ph group at N(1), after a successful chromatographic separation, the less stable **9k** underwent isomerization in CDCl_3_ solution (at rt) and no registration of NMR spectra of this product, without contamination with **10k**, was feasible.

**Figure 1:**
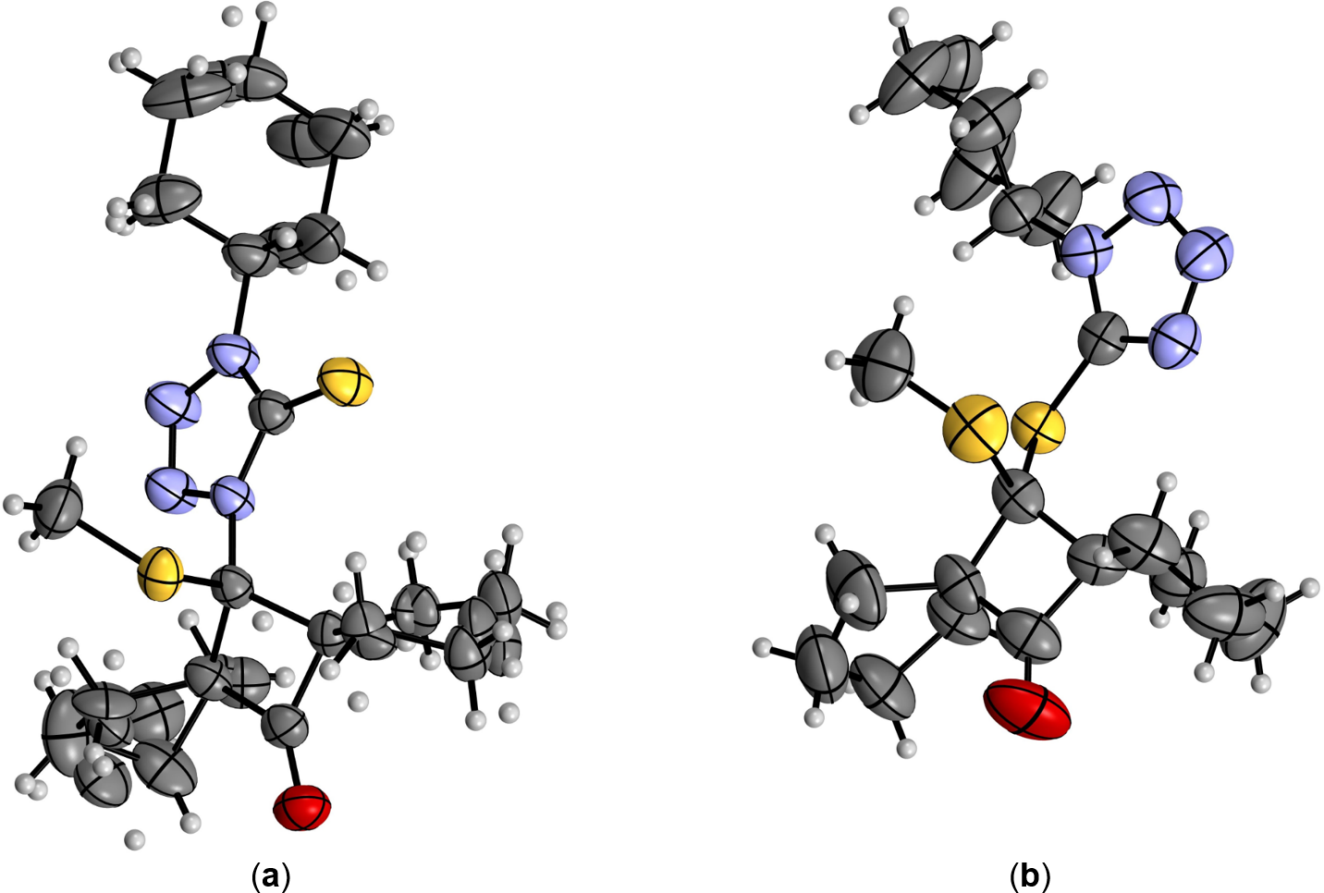
(a) Molecular structure of the N-insertion product (thioaminal) **9i**. Atoms are represented by thermal ellipsoids (50%). For graphics with atoms labelling see Figure S22(a) (Supporting Information File 1); (b) molecular structure of the S-insertion product (dithioacetal) **10i**. Atoms are represented by thermal ellipsoids (50%). For graphics with atoms labelling see Figure S22(b) in Supporting Information File 1.

Thioaminal **9i** and dithioacetal **10i** derived from 1-cyclohexyl-5-mercapto-4*H*-tetrazole (**4b**) could be isolated as stable, crystalline products and the postulated structures were unambiguously confirmed by the single crystal X-ray diffraction analysis (Figure 1a and Figure 1b, respectively).

Single crystals of **9i** and **10i** were obtained by slow evaporation from a hexane/CH_2_Cl_2_ solution and subsequently used for crystallographic X-ray measurements. In the case of **9i**, the molecule is strongly disordered in the outer carbon ring regions. However, the quality of the X-ray measurements and structure determination meets all high standards for reliable data. The same applies to the crystal data of **10i**, even though its crystal structure contains four independent molecules per unit cell. These molecules exhibit slight differences in their geometry, ensuring that no additional symmetry elements were omitted during structure refinement.

The obtained data undoubtedly confirmed the anticipated structures and their experimental characterizations. Thioaminal **9i** and dithioacetal **10i** differ significantly in the substitution of the tetrazole ring. This crucial difference in the molecular structures is best reflected in the length of the respective C–S bonds. In **9i**, this bond should be considered as a standard thiocarbonyl group, whereas in **10i** it corresponds more closely to a C–S single bond. Consequently, in **9i**, the C(20)–S(21) bond (formally a double bond) has a length of 1.650(13) Å. For comparison, this distance in other tetrazole-thiones has been reported as 1.669(3) Å [29] and 1.719(6) Å [30]. Thus, the C=S bond in **9i** appears slightly shorter (more localized) than in comparable examples. On the other hand, the C(20)–S(21) bond length in **9i** is typical for a C=S double bond. Conversely, in **10i** the corresponding C1(A–D)–S16(A–D) bond length (with A–D indicating the four independent molecules in the unit cell) ranges from 1.823 to 1.831(4) Å. This distance is characteristic of a formal C–S single bond [31] (and references therein).

Finally, trapping reactions (toluene, 60 °C) performed with **2d**, functionalized with two spiro-cyclohexyl rings, and 5-mercaptotetrazoles **4a**‒**e** led selectively to dithioacetals **10m**‒**q**, which could be separated chromatographically as crystalline materials in good yields (46‒89%). Isomeric thioaminals of type **9** were not observed in the ^1^H NMR spectra registered for crude reaction mixtures (Table 1).

**Scheme 6:**
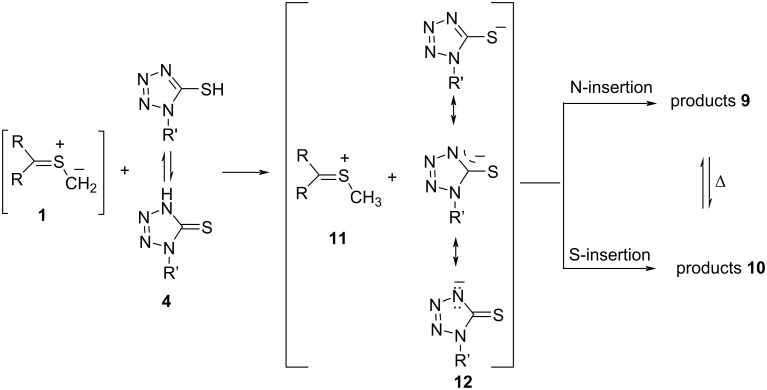
Stepwise mechanism of the competitive N- and S-insertion reactions between the in situ-generated thiocarbonyl *S*-methanides **1** and 1-substituted 5-mercapto-1*H*-tetrazoles **4**.

The mechanism of the studied reactions deserves a brief comment and should help to clarify whether the formation of **9** and **10** results from the ambident reactivity of 5-mercapto-1*H*-tetrazoles, suggested in some earlier publications [15], or/and from secondary processes, like an intramolecular rearrangement. Taking into account the postulated basicity (and nucleophilicity) of thiocarbonyl *S*-methanides **1** derived from cycloaliphatic thioketones [6], the initiating step of these processes can be presented as protonation of the terminal S‒CH_2_ position, leading to the formation of the sulfonium cation **11** and the delocalized heterocyclic anion **12** (Scheme 6). In the next step, competitive addition of both intermediate species yields either thioaminals **9** or dithioacetals **10**. However, a slow isomerization of the thermodynamically less stable **9** into the more stable **10** was observed in some cases (derived from **1c**) in the CDCl_3_ solution at room temperature. Therefore, it seems likely that the first step of the reaction is the N–H insertion process.

**Scheme 7:**
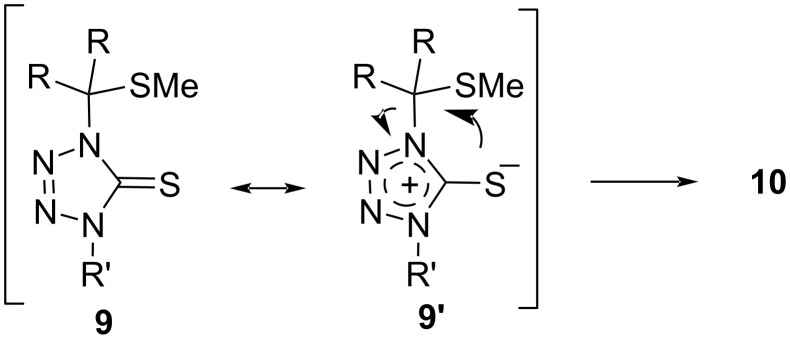
Mechanism of the isomerization of initially formed thioaminals **9** to dithioacetals **10**.

The compositions of the obtained crude mixtures suggest that the final addition step depends on both steric factors in **11** and the electronic structure of the reactive anion **12**. In general, growing steric hindrance prefers the S-attack and, very likely, hinders rearrangement leading to thioaminals **9**. This hypothesis is supported by the fact that no formation of products **9** is observed with the sterically most hindered dispiro-cyclohexyl-substituted thiocarbonyl *S*-methanide **2d**. Notably, in some cases spontaneous isomerization of the generally less stable thioaminals **9** in CDCl_3_ solution at room temperature was also observed. It seems likely that this isomerization follows an intramolecular pathway, which is accelerated in the zwitterionic form **9’** (Scheme 7).

Striking differences in the structure of products obtained under identical conditions (THF, 45 °C) from **1a** (only thioaminals **9**) and from **1b** (only dithioacetals **10**), deserves also a brief comment. Notably, there were no substantial differences in reactivity of thiocarbonyl *S*-methanides **1a** and **1b** reported in earlier studies on their [3 + 2]-cycloadditions [6,10–11] or insertion reactions [12–14]. Therefore, a likely interpretation for the observed difference in the present study can be based on the assumption that the initial products are thioaminals **9**, which under the reaction conditions or later on, during the storage in CDCl_3_ solution, undergo an intramolecular rearrangement presented in Scheme 7. Moreover, the differences observed in the structures of products obtained in reactions with structurally similar cyclobutanone derivatives **1b**, **1c** and **1d** suggest that the type of the spiro-substituent may play an important role in the rearrangement process. Whereas thioaminals **9** obtained in reactions with **1b** and **1d** tend to easily undergo the rearrangement leading to the corresponding dithioacetals **10**, the analogous process in the series of products derived from **1c** is slower (and reversible?), and therefore, corresponding products of both N- and S-insertion are side by side observed as components of the crude reaction mixtures and their ratio was always ca. 55:45. In the latter case, chromatographic separation of the isomeric products was a feasible operation.

### Cytotoxicity of selected thioaminals **9** and dithioacetals **10** in cancer and non-cancer cells

Cytotoxicity investigations represent a pivotal component in the realm of pharmaceutical development and contemporary medicine. In vitro assays constitute a rapid method for evaluating the influence of particular chemical compounds on cell lines. The most widely recognised method for assessing the impact of specific chemical compounds on specific cell types is the MTT (3-(4,5-dimethylthiazol-2-yl)-2,5-diphenyltetrazolium bromide) assay. The reduction of the tetrazolium structure in the MTT dye results in the formation of a coloured formazan that can be detected by spectrophotometry.

Cytotoxic properties of the studied compounds were assessed on non-cancer as well as cancer cell lines. Cytotoxicity was established by measurement of 50% inhibition of cell growth by MTT assay and expressed as CC_50_ parameter (50% cytotoxic concentration) [32]. All results are gathered in Table 2.

**Table 2:** The results of cytotoxicity (CC_50_) obtained for investigated compounds in concentrations ranging from 0.1 to 1000 µM.

No.	Compound	Non-cancer cells CC_50_ [uM]				Cancer cells CC_50_ [uM]			
		MRC5	Vero	LLCMK2	NCTC clone 929	HepG2	A549	HeLa	T98G

1	**9a**	>1000	965.04 ± 123.59	>1000	>1000	>1000	>1000	6.35 ± 1.75	>1000
2	**9h**	>1000	418.71 ± 71.83	>1000	>1000	>1000	>1000	2.11 ± 0.53	936.01 ± 127.99
3	**9i**	389.401 ± 26.62	951.17 ± 78.25	>1000	>1000	>1000	>1000	74.12 ± 15.20	>1000
4	**9j**	>1000	156.63 ± 15.82	989.12 ± 24.33	213.22 ± 59.01	>1000	>1000	71.86 ± 2.66	976.27 ± 47.46
5	**10h**	>1000	>1000	>1000	>1000	>1000	>1000	46.20 ± 8.88	>1000
6	**10i**	>1000	42.53 ± 4.92	67.06 ± 8.03	284.61 ± 58.96	>1000	>1000	9.26 ± 3.14	>1000
7	**10l**	>1000	474.97 ± 73.80	645.69 ± 29.81	>1000	>1000	>1000	215.31 ± 27.51	>1000
8	**10o**	997.518 ± 117.91	48.02 ± 8.30	90.01 ± 6.90	6.85 ± 1.34	>1000	>1000	35.68 ± 4.33	387.92 ± 70.59
9	**10q**	>1000	172.99 ± 20.92	317.76 ± 31.27	>1000	>1000	>1000	47.48 ± 5.68	>1000

In general, the tested compounds did not demonstrate high levels of toxicity towards the investigated non-cancer cell lines. Among them, Vero cells showed the highest sensitivity, however, it should be noted that the observed CC_50_ values can be described as medium. The exception is compound **10o**, which was highly toxic towards NCTC clone 929 (CC_50_ = 6.85 ± 1.34 µM). The MRC-5 line exhibited the lowest level of sensitivity.

The cancer cell lines HepG2, A549 and T98G were found to be insensitive to the investigated compounds (CC_50_ > 1000 µM for HepG2, A549 and CC_50_ > 300 µM for T98G), whereas HeLa cells demonstrated increased sensitivity. The most active compounds **9a**, **9h** and **10i**, revealed their toxicity with CC_50_ < 10 µM (6.35 ± 1.75 µM; 2.11 ± 0.53 µM and 9.26 ± 3.14 µM, respectively). In the same cell line, the remaining compounds demonstrated cytotoxic activity in the range of 35.68‒74.12 µM (**10o**, **10h**, **10q**, **9j**, and **9i**), while the least active compound **10l** showed a level of toxicity an order of magnitude lower (CC_50_ = 215.31 ± 27.51 µM). The results obtained in this study demonstrate that the compounds exhibit selective activity towards HeLa cells.

## Conclusion

The presented study showed that the sterically crowded thioketones **7c**,**d** smoothly undergo the anticipated [3 + 2]-cycloaddition with diazomethane and 1,3,4-thiadiazolines **2c** and **2d**, respectively, were formed with complete regioselectivity in high yields. Upon heating to 65 °C in toluene solution, in analogy to the well-known compounds **2a** and **2b**, they extruded N*_2_* and the in situ-generated reactive thiocarbonyl *S*-methanides **1c** and **1d**, in absence of any trapping reagent, underwent 1,3-dipolar electrocyclization yielding the corresponding, bulky substituted, thiiranes **8a** and **8b**. However, generation of thiocarbonyl *S*-methanides **1** in the presence of enolizable 1-substituted 5-mercapto-1*H*-tetrazoles **4** led to their efficient trapping and depending on the structure of **1** and the substituent located at N-1 in tetrazole **4**, either thioaminals **9** or dithioacetals **10** were observed as products of the N- or S-insertion reaction, respectively. However, in some cases, formation of mixtures of products **9** and **10** were obtained and these results can be explained by ambident reactivity of the enolizable mercapto heterocycles **4**. Notably, in some instances, depending on the substitution pattern, thermodynamically less stable thioaminals **9** underwent thermal isomerization in CDCl_3_ solution yielding the corresponding dithioacetals **10**. Comparison of the results obtained with dispiro-substituted thiocarbonyl *S*-methanides **4c** (less hindered) and **4d** (more hindered) suggests that the steric hindrance can play an important role in the trapping process.

It is worth of emphasizing that thioaminals [33] and dithioacetals [34] constitute important groups of building blocks, which are of interest not only for pharmaceutically oriented studies [35] but also for polymer chemistry [36] and the crop protection industry [37] as well. Therefore, in extension of a typically synthetic work, a preliminary study on biological activity of the hitherto unreported 1*H*-tetrazole derivatives such as thioaminals **9** and dithioacetals **10** was carried out and it demonstrated that some of them act as potent cytotoxic agents against certain cancer cells.

In addition, the newly synthesized, sterically crowded 1,3,4-thiadiazolines **2c**,**d** can be considered as attractive precursors of the corresponding bulky substituted thiiranes **8** and ethylenes derived from the corresponding cyclobutanones, which are easily accessible via desulfurization reaction of the thiiranes described in the present study (via the Barton–Kellogg reaction [38–39]). This type of ethylenic compounds is of interest, e.g., for structural, coordination and materials chemistry, as well [39–43]. Moreover, the presented study opens new perspectives for the development of studies focused on the exploration of sterically over-crowded thiocarbonyl *S*-methanides **1c**,**d** in 1,3-dipolar cycloadditions with a plethora of various dipolarophiles (C=C, C≡C, C=N, C=O, C=S, N=N, etc.).

## Experimental

### General information

Commercial chemicals and solvents were used as received. If not stated otherwise, products were purified by filtration through short silica gel plugs (200–400 mesh) by using freshly distilled solvents as eluents or by recrystallization. Melting points were determined in capillaries with an Aldrich Melt-Temp II and they are uncorrected. NMR spectra were taken with a Bruker AVIII spectrometer (^1^H NMR (600 MHz); ^13^C NMR (151 MHz); chemical shifts are relative to residual undeuterated solvent peaks (CDCl_3_: ^1^H NMR δ = 7.25, ^13^C NMR δ = 77.00). Elemental analyses were obtained with a Vario EL III (Elementar Analysensysteme GmbH) instrument.

### Starting materials

Adamantanethione (**7a**) and 3-thioxo-2,2,4,4-tetramethylcyclobutanone (**7b**) as well as the corresponding spiro-1,3,4-thiadiazolines **2a** and **2b** derived therefrom were obtained following published procedures [6–10,17–18]. Two sterically crowded dispiro-thioketones **7c** (12-thioxo-dispiro[4.1.4.1]dodecan-6-one) and **7d** (14-thioxo-dispiro[5.1.5.1]tetradecan-7-one) were prepared according to published procedures [23,26]. 1-Substituted 5-mercapto-1*H*-tetrazoles **4a**‒**e** were used as commercial reagents (**4a**, **4d**) or they were synthetized following published procedures (for synthesis of **4b**,**c**, and **4e**, see ref. [15]). The solution of diazomethane (CH_2_N_2_) in pentane was prepared without distillation and determination of the concentration following a procedure described in an earlier publication [28].

**Preparation of 2c as a new precursor of thiocarbonyl *S*-methanide 1c:** To a magnetically stirred solution of the thioketone **7c** (833.0 mg, 4 mmol) in 5 mL pentane, placed in a water/ice bath, dry solution of diazomethane in pentane was added dropwise until the red color of starting **7c** vanished. The colorless solution was cooled down in a dry ice container, and after few hours of cooling a colorless solid was separated. The crystalline material was filtered off and dried in the air at rt.

**10-Thia-7,8-diazatrispiro[4.0.4^6^.0.4^11^.1^5^]hexadec-7-en-16-one (2c):** Yield: 910.0 mg (91%); mp 53‒55 °C (dec.); ^1^H NMR (CDCl_3_) δ 1.32–1.39 (m, 2H), 1.39–1.48 (m, 2H), 1.53–1.60 (m, 2H), 1.60–1.71 (m, 4H) 1.89–2.01 (m, 4H), 2.08–2.15 (m, 2H), 5.77 (s, 2H, S–C*H*_2_–N); ^13^C NMR (CDCl_3_) δ 25.4, 25.6, 30.3, 36.2, 75.2, 82.9 (S–*C*H_2_–N), 114.9, 217.3 (C=O); Anal. calcd for C_13_H_18_N_2_OS (250.36): C, 62.36; H, 7.25; N, 11.19; S, 12.81; found: C, 62.36; H, 7.43; N, 10.97; S, 12.85.

**Thermal decomposition of dispiro-1,3,4-thiadiazoline 2c:** A magnetically stirred solution of **2c** (125.0 mg, 0.5 mmol) in 1 mL THF was heated in an oil bath at 65 °C and the evolution of nitrogen gas was controlled with a nitrometer (gas burette) connected with the flask. After ca. 3 h evolution of nitrogen was complete (ca. 13 mL N_2_ was collected in the nitrometer). The solvent was evaporated in vacuo and the residue was examined by ^1^H NMR spectroscopy. A characteristic singlet of the S–CH_2_ fragment was found in the region of 2.62 ppm. The pure product **8a** was isolated by preparative layer chromatography.

**1-Thiatrispiro[2.0.4^4^.1.4^10^.0^3^]tetradecan-9-one (8a):** Yield: 68 mg (62%); colorless, viscous oil; ^1^H NMR (CDCl_3_) δ 1.42–1.72 (m, 10H), 1.79–1.86 (m, 2H), 1.90–1.97 (m, 2H), 2.01–2.08 (m, 2H) 2.62 (s, 2H, S–C*H*_2_–C); ^13^C NMR (CDCl_3_) δ 25.7, 26.1, 26.5 (S–*C*H_2_), 33.4, 37.2, 61.8, 70.1, 219.9 (*C*=O); Anal. calcd for C_13_H_18_OS (222.34): C, 70.22; H, 8.16; S, 14.42; found: C, 70.20; H, 8.22; S, 14.34.

**Reactions of the in situ-generated thiocarbonyl *S*-methanides 1a**‒**d with 5-mercapto-1-methyl-1*H*-tetrazole (4a):** A magnetically stirred solution of 0.50 mmol (58 mg) **4a** and 0.55 mmol of the corresponding precursor **2a**–**d** (see Table 1) in 1 mL of THF was heated at 45 °C (for **2a** and **2b**) or in 1 mL of toluene at 65 °C (for **2c** and **2d**). The evolution of nitrogen was controlled using a nitrometer (gas burette) connected with the reaction flask. In all cases the reaction was completed after ca. 3 h. After this time, the solvent was evaporated, and the residue was analyzed by ^1^H NMR. Depending on the composition of the crude product, this material was either separated by preparative layer chromatography on the plates coated with silica gel (CH_2_Cl_2_ as the mobile phase) (separation of **9h** and **10h**) or crystallized from hexane/CH_2_Cl_2_ mixture (**9a** and **10m**) or from MeOH (**10c**). Analytically pure samples of products isolated after chromatography were prepared after recrystallization from hexane/CH_2_Cl_2_ mixture.

**1-Methyl-4-[2-(methylthio)adamantan-2-yl]-1,4-dihydro-1*H*-tetrazole-5-thione (9a):** Yield 97 mg (66%), colorless crystals; mp 107‒109 °C (hexane/CH_2_Cl_2_); ^1^H NMR (CDCl_3_) δ 1.62‒1.94 (m, 10H, Ad-skeleton), 1.86 (s, 3H, SMe), 2.37‒2.44 (m, 1H), 2.47‒2.54 (m, 1H), 2.98‒3.03 (m, 1H), 3.86 (s, 3H, NMe), 4.34‒4.38 (m, 1H); ^13^C NMR (CDCl_3_) δ 10.3 (S*C*H_3_), 26.3, 26.8, 31.6, 32.7, 33.0, 33.2, 33.5 (N*C*H_3_), 34.5, 37.8, 81.2 (N‒*C*‒S), 162.9 (*C*=S); Anal. calcd for C_13_H_20_N_4_S_2_ (296.45): C, 52.67; H, 6.80; N, 18.90; S, 21.63; found: C, 52.63; H, 6.92; N, 18.81; S, 21.74.

**2,2,4,4-Tetramethyl-3-[(1-methyl-1*H*-tetrazol-5-yl)thio]-3-(methylthio)cyclobutan-1-one (10c):** Yield 120 mg (84%); colorless crystals; mp 136‒138 °C (MeOH); ^1^H NMR (CDCl_3_) δ 1.50 (s, 6H, 2Me), 1.66 (s, 6H, 2Me), 2.06 (s, 3H, SMe), 4.03 (s, 3H, NMe); ^13^C NMR (CDCl_3_) δ 15.8 (S*C*H_3_). 20.8 (2Me), 23.9 (2Me), 33.9 (N*C*H_3_), 68.6, 74.8 (S‒*C*‒S), 151.3 (N=*C*−S), 216.8 (*C*=O); Anal. calcd for C_11_H_18_N_4_OS_2_ (286.42): C, 46.13; H, 6.33; N, 19.56; S, 22.39; found: C, 46.29; H, 6.33; N, 19.62; S, 22.36.

**12-(4-Methyl-5-thioxo-4,5-dihydro-1*H*-tetrazol-1-yl)-12-(methylthio)dispiro-[4.1.4^7^.1^5^]dodecan-6-one (9h):** Isolated as the less polar fraction by PLC (SiO_2_, CH_2_Cl_2_). Yield: 61 mg (36%); colorless crystals; mp 127‒129 °C (hexane/CH_2_Cl_2_); ^1^H NMR (CDCl_3_) δ 1.67‒1.76 (m, 4H), 1.80 (s, 3H, SMe), 1.85‒1.96 (m, 6H), 2.05‒2.14 (br.m, 2H), 2.27‒2.35 (br.m, 2H), 2.43‒2.58 (br.m, 2H), 3.90 (s, 3H, NMe); ^13^C NMR (CDCl_3_) δ 13.6 (S*C*H_3_), 26.1, 26.5, 31.6 (br), 34.8, 35.6 (br), 74.7 (S‒*C*‒S), 164.5 (*C*=S), 215.2 (*C*=O); Anal. calcd for C_15_H_22_N_4_OS_2_ (338.49): C, 53.22; H, 6.55; N, 16.55; S, 18.94; found: C, 53.19; H, 6.29; N, 16.63; S, 18.94.

**12-[(1-Methyl-1*H*-tetrazol-5-yl)thio]-12-(methylthio)dispiro[4.1.4^7^.1^5^]dodecan-6-one (10h):** Isolated as the more polar fraction by PLC (SiO_2_, CH_2_Cl_2_): Yield: 80 mg (47%); colorless crystals; mp 109‒111 °C (hexane/CH_2_Cl_2_); ^1^H NMR (CDCl_3_) δ 1.71‒1.83 (m, 8H), 1.96‒2.03 (m, 2H), 2.05‒2.12 (m, 2H), 2.07 (s, 3H, SMe), 2.22‒2.34 (m, 4H), 4.03 (s, 3H, NMe); ^13^C NMR (CDCl_3_) δ 15.4 (S*C*H_3_), 25.4, 25.8, 32.7, 34.0, 35.7, 72.1, 78.2 (S−*C*−S), 151.0 (N=*C*−S), 216.6 (*C*=O); Anal. calcd for C_15_H_22_N_4_OS_2_ (338.49): C, 53.22; H, 6.55; N, 16.55; S, 18.94; found: C, 53.22; H, 6.41; N, 16.65; S, 19.05.

**14-[(1-Methyl-1*H*-tetrazol-5-yl)thio]-14-(methylthio)dispiro[5.1.5^8^.1^6^]tetradecan-7-one (10m):** Yield 85 mg (46%); colorless crystals; mp 191‒193 °C (hexane/CH_2_Cl_2_); ^1^H NMR (CDCl_3_) δ 1.13‒1.23 (m, 2H), 1.64‒1.73 (m, 10H), 1.74‒1.83 (m, 2H), 1.95‒2.04 (m, 2H), 2.02 (s, 3H, SMe), 2.34‒2.42 (m, 4H), 4.02 (s, 3H, NMe); ^13^C NMR (CDCl_3_) δ 15.3 (S*C*H_3_), 23.5, 23.6, 25.3, 31.1, 33.9, 34.0 (6 signals for 10*C*H_2_), 70.8 (2*C*_q_), 75.9 (S‒*C*‒S), 151.3 (N=*C*‒S), 215.7 (*C*=O); Anal calcd for C_17_H_26_N_4_OS_2_ (366.54): C, 55.70; H, 7.15; N, 15.28; S, 17.50; found: C, 55.55; H, 7.04; N, 15.15; S, 17.68.

## Supporting Information

CCDC-2420216 and CCDC-2420217 contain the supplementary crystallographic data for this paper. These data can be obtained free of charge from the Cambridge Crystallographic Data Centre via http://www.ccdc.cam.ac.uk/structures.

File 1 General information and experimental data of all isolated products, procedure for determination of biological activity, details of the crystal structure determination, and copies of ^1^H and ^13^C NMR spectra for all products.

## Data Availability

The data that supports the findings of this study is available from the corresponding author upon reasonable request.
