# Supplementary material for: Ambident reactivity of enolizable 5-mercapto-1H-tetrazoles in trapping reactions with in situ-generated thiocarbonyl S-methanides derived from sterically crowded cycloaliphatic thioketones
Source: Beilstein J Org Chem. 2025 Jul 23;21:1508–19. doi: 10.3762/bjoc.21.113 (PMC12302482; doi:10.3762/bjoc.21.113)
Supplement: File 1 — General information and experimental data of all isolated products, procedure for determination of biological activity, details of the crystal structure determination, and copies of 1H and 13C NMR spectra for all products. [file Beilstein_J_Org_Chem-21-1508-s001.pdf]

BEILSTEIN JOURNAL OF ORGANIC CHEMISTRY

Supporting Information

for

Ambident reactivity of enolizable 5-mercapto-1*H*-tetrazoles in trapping reactions with in situ-generated thiocarbonyl *S*-methanides derived from sterically crowded cycloaliphatic thioketones

Grzegorz Mlostoń, Małgorzata Celeda, Marcin Palusiak, Heinz Heimgartner, Marta Denel-Bobrowska and Agnieszka B. Olejniczak

Beilstein J. Org. Chem. **2025,** *21,* 1508–1519. doi:10.3762/bjoc.21.113

General information and experimental data of all isolated products, procedure for determination of biological activity, details of the crystal structure determination, and copies of ^{1}H and ^{13}C NMR spectra for all products

License and Terms: This is a supporting information file under the terms of the Creative Commons Attribution License (https://creativecommons.org/licenses/by/4.0). Please note that the reuse, redistribution and reproduction in particular requires that the author(s) and source are credited and that individual graphics may be subject to special legal provisions.
The license is subject to the *Beilstein Journal of Organic Chemistry* terms and conditions: (https://www.beilstein-journals.org/bjoc/terms)

Contents:

Section 1: *General information* S3

Section 2: *Thioaminals* **9b**,**c**,**e-t** *and dithioacetals* **10b,c**,**e-t** S4

Section 3: *^{1}H and ^{13}C NMR spectra registered for thioaminals* **9** *and dithioacetals* **10** S17

Section 4: *X-Ray structure determination of thioaminal* ***9i*** *and dithioacetal* ***10i***; *Table S1* S43

Section 5: *List of references* S46

Section 1: *General information* (see also in Experimental)

1.1. *Reagents and solvents*: unless stated otherwise, used as commercially available with reagent grade and did not require further purification.

1.2. *NMR spectroscopy*: NMR spectra were recorded with a Bruker AVIII 600 (^{1}H NMR [600 MHz];^{13}C NMR [151 MHz]) or with a Varian Gemini 2000BB 200 MHz (^{19}F NMR [188 MHz]) instruments. Chemical shifts are reported relative to solvent residual peaks (^{1}H NMR, δ = 7.25 ppm [$CDCl_3$]; ^{13}C NMR, δ = 77.0 ppm [$CDCl_3$]).

1.3. *Optical rotations*: determined with an Anton Paar MCP 500 polarimeter at the temperatures indicated.

1.4. *Flash chromatography*: products were purified by flash column chromatography (CC) on silica gel (230–400 mesh, Merck).

1.5. *Preparative thin-layer chromatography (PLC)* was carried out using 20x20 cm glass plates coated with silica (60 PF_{254}, Merck). In all cases separation of products was achieved using dichloromethane/ethanol (98:2) mixture.

1.6. *Melting points* were determined in capillaries with a MEL-TEMP apparatus (Aldrich) and are uncorrected.

1.7. *Elemental analyses* were obtained with a Vario EL III (Elementar Analysensysteme GmbH) instrument.

1.8. *Preparation of starting materials and general procedures*: see also in Experimental (see the main manuscript)

1.9. *In vitro cytotoxic activity*: non-cancer cell lines: Vero (Cercopithecus aethiops normal kidney cells); LLCMK2 (Macaca mulatta normal kidney cells), MRC-5 (Human lung normal fibroblasts), NCTC clone 929 (CCL-1, Mus musculus normal subcutaneous connective tissue cells). Cancer cell lines: HeLa (Human cervix adenocarcinoma cells), T98G (Human glioblastoma multiforme cells), A549 (Human lung carcinoma cells), HepG2 (Human hepatocellular carcinoma). Cell lines were purchased from American Type Culture Collection (ATCC, Manassas, Virginia, USA). All tested compounds were dissolved in DMSO (dimethylsulfoxide, Sigma-Aldrich, Darmstadt, Germany) and then suspended in Minimum Essential Medium (MEM; Sigma-Aldrich, Darmstadt, Germany) supplemented with 10% heat-inactivated fetal bovine serum (FBS; Sigma-Aldrich Darmstadt, Germany) and 1% penicillin/streptomycin mixture (10 000 units/mL penicillin G with 10 mg/mL streptomycin (Sigma-Aldrich Darmstadt, Germany)).

Investigated cells were propagated in Minimum Essential Medium supplemented with 10% heat-inactivated fetal bovine serum, 2 mM L-glutamine and 1% penicillin/streptomycin mixture. After reaching 80–90% confluency, cells were harvested with trypsin (Life Technologies, Warsaw, Poland) and seeded into 96-well microplates at 2 x 10^4 cells/well. After overnight incubation at 37 °C in a humidified atmosphere containing 5% CO_2, the culture medium was replaced with a 100 μL freshly prepared solution of tested compounds diluted with medium supplemented with 10% FBS, and antibiotics to obtain compounds concentrations in the range from 0.1 to 1000 μM. The cytotoxicity was evaluated by the MTT assay. All experiments were carried out in triplicate, in two independent experiments. Cells exposed on investigated compounds and unexposed cells (control group) were incubated at 37 °C for 48 h in a humidified atmosphere containing 5% CO_2 [1]. After the incubation, cells were treated for 2 h with 3-(4,5-dimethylthiazol-2-yl)-2,5-diphenyltetrazolium bromide dye solution (MTT, Sigma-Aldrich Darmstadt, Germany) and lysed with solvent solution (100 μL) containing: DMF (Sigma-Aldrich Darmstadt, Germany) (45 mL), SDS (Sigma-Aldrich Darmstadt, Germany) (13.5 g) and distilled water (55 mL). After overnight incubation at 37 °C, optical density at 550 nm and with a reference wavelength of 670 nm was measured on a microplate spectrophotometer Varioskan Lux (Thermo Fisher Scientific, Waltham, Massachusetts, USA). The cytotoxic concentration (CC_{50}) was defined as the concentration required to reduce cell viability by 50% compared to untreated controls and was calculated by linear regression analysis of the dose-response curves obtained from the data.

Section 2**: Thioaminals **9** and dithioacetals **10

***Reactions of the in situ-generated thiocarbonyl S-methanides* 1a–d *with 5-mercapto-1H-tetrazoles* 4b–e – general procedure:** A magnetically stirred solution of 0.50 mmol of the corresponding tetrazole derivative **4** and 0.55 mmol of the corresponding thiocarbonyl ylide precursor **2** (see Table 1) in 1 mL of THF was heated at 45 °C (for **2a** and **2b**) or in 1 mL toluene at 65 °C (for **2c** and **2d**). The evolution of nitrogen was controlled using a nitrometer (gas burette) connected with the reaction flask. In all cases the reaction was completed after ca. 3 h. After this time, the solvent was evaporated, and the residue was analyzed by ^{1}H NMR. Depending on the composition of the crude product, this material was either separated by preparative layer chromatography on the plates coated with silica gel or crystallized from an appropriate solvent (hexane/CH_2Cl_2 mixture; no MeOH can be used as a solvent). If necessary,

the products isolated after chromatography were purified by recrystallization from corresponding solvents (see below).

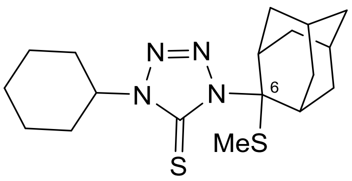

9b

*1-Cyclohexyl-4-(2-(methylthio)(adamantan-2-yl)-1,4-dihydro-*5*H-tetrazole-5-thione* (**9b**): yield 110 mg (52%), colorless crystals, m.p. 123–125 °C (hexane/CH_2Cl_2).

^{1}H NMR ($CDCl_3$): δ 1.21–1.34 (*m*, 1H), 1.40–1.53 (*m*, 2H), 1.59–1.68 (*m*, 1H), 1.69–1.95 (*m*, 14H). 1.82 (*s*, 3H, SMe), 2.05–2.12 (*m*, 2H), 2.37–2.43 (*m*, 1H), 2.50–2.56 (*m*, 1H), 2.98–3.04 (*m*, 1H), 4.39-4.44 (*m*, 1H), 4.71–4.79 (*m*, 1H).
^{13}C NMR ($CDCl_3$): δ 10.3 (S*C*H_3), 25.1, 25.2, 26.4, 26.9, 30.9, 31.2, 32.0, 33.0, 33.2, 33.3, 33.5, 33.6, 33.7, 37.8, 56.8, 81.1 (N−*C*−S), 161.2 (C=S).
EA for $C_{18}H_{28}N_4S_2$ (364.57): calc. C 59.30, H 7.74, N 15.37, S 17.59; found C 59.25, H 7.67, N 15.35, S 17.72.

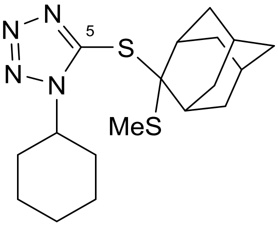

10b

3-((1-Cyclohexyl-5-(2-(methylthio)adamantan-2-yl)thio)-1H-tetrazole (**10b**): this product was not detected in the crude reaction mixture (by ^{1}H NMR).

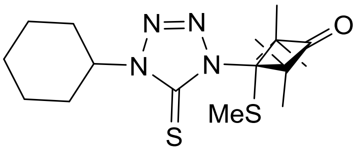

9d

3-(4-Cyclohexyl-5-thioxo-4,5-dihydro-1H-tetrazol-1-yl)-2,2,4,4-tetramethyl-3-(methylthio)-cyclobutan-1-one (**9d**): this product was not detected in the crude reaction mixture..

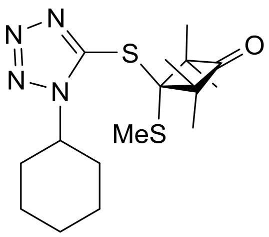

10d

3-((1-Cyclohexyl-1H-tetrazol-5-yl)thio)-2,2,4,4-tetramethyl-3-(methylthio)cyclobutan-1-one (**10d**): yield 134 mg (76%), colorless crystals; m.p. 147–149 °C (MeOH).

^{1}H NMR ($CDCl_3$): δ 1.29–1.38 (*m*, 1H), 1.40–1.49 (*m*, 2H), 1.51 (*s*, 6H, 2Me), 1.67 (*s*, 6H, 2Me), 1.74–1.80 (*m*, 1H), 1.94–2.01 (*m*, 6H), 2.04 (*s*, 3H, SMe), 4.27–4.36 (*m*, 1H).
^{13}C NMR ($CDCl_3$): δ 15.8 (S*C*H_3). 20.7 (2Me), 24.0 (2Me), 24.8 (CH_2), 25.3 (2CH_2), 32.9, 58.4, 68.6, 74.6 (S−*C*−S), 149.8 (N=*C*−S), 217.1 (*C*=O).
EA for $C_{16}H_{26}N_4OS_2$ (354.53): calc. C 54.20, H 7.39, N 15.80, S 18.09; found C 54.11, H 7.38, N 15.66, S 18.05.

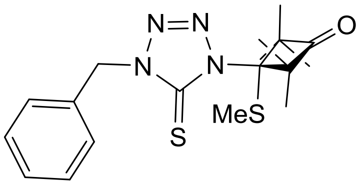

9e

3-(4-Benzyl-5-thioxo-4,5-dihydro-1H-tetrazol-1-yl)-2,2,4,4-tetramethyl-3-(methylthio)-2-cyclobutan-1-one (**9e**): this product was not detected in the crude reaction mixture.

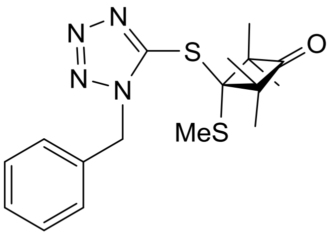

10e

3-((1-Benzyl-1H-tetrazol-5-yl)thio)-2,2,4,4-tetramethyl-3-(methylthio)cyclobutan-1-one (**10e**): yield 130 mg (72%), colorless crystals; m.p. 123–125 °C (diisopropyl ether/CH_2Cl_2).

^{1}H NMR ($CDCl_3$): δ 1.48 (s, 6H, 2Me), 1.61 (s, 6H, 2Me), 1.81 (SMe), 5.51 (s, 2H, CH_2), 7.25–7.29 and 7.31–7.38 (2*m*, 5H, C_6H_5).

^{13}C NMR ($CDCl_3$): δ 15.5 (S*C*H_3). 20.7 (2Me), 23.8 (2Me), 51.2, 68.6, 74.8 (S–*C*–S) 127.8 (2H*C*$_{ar}$), 128.9 (H*C*$_{ar}$), 129.1 (2H*C*$_{ar}$), 133.2 (*C*$_{ar}$), 151.2 (N=*C*−S), 216.9 (*C*=O).
EA for $C_{17}H_{22}N_4OS_2$ (362.51): calc. C 56.32, H 6.12, N 15.45, S 17.69; found C 56.37, H 6.04, N 15.53, S 17.86.

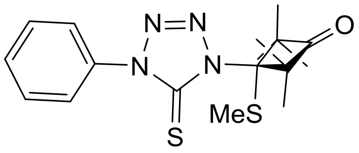

9f

3-(4-Phenyl-5-thioxo-4,5-dihydro-1H-tetrazol-1-yl)-2,2,4,4-tetramethyl-3-(methylthio)-2-cyclobutan-1-one (**9f**): this product was not detected in the crude reaction mixture.

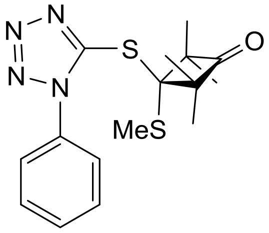

10f

2,2,4,4-Tetramethyl-3-(methylthio)-3-((1-phenyl-1H-tetrazol-5-yl)thio)cyclobutan-1-one (**10f**): yield 142 mg (82%), colorless crystals; m.p. 128–130 °C (MeOH).

^{1}H NMR ($CDCl_3$): δ 1.47 (s, 6H, 2Me), 1.66 (s, 6H, 2Me), 1.77 (*s*, 3H, SMe), 7.49–7.64 (*m*, 5H, C_6H_5).
^{13}C NMR ($CDCl_3$): δ 15.7 (S*C*H_3). 20.6 (2Me), 24.0 (2Me), 68.6 (2*C*$_q$), 75.2 (S–*C*–S), 124.6 (2H*C*$_{ar}$), 129.7 (2H*C*$_{ar}$), 130.5 (H*C*$_{ar}$), 133.7 (*C*$_{ar}$), 151.3 (N=*C*−S), 217.0 (*C*=O).
EA for $C_{16}H_{20}N_4OS_2$ (348.48): calc. C 55.14, H 5.78, N 16.08, S 18.40; found C 55.14, H 5.77, N 16.09, S 18.33.

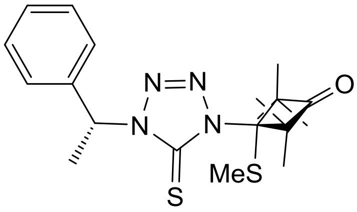

9g

2,2,4,4-Tetramethyl-3-(methylthio)-3-(4-((R)-1-phenylethyl)-5-thioxo-4,5-dihydro-1H-tetrazol-1-yl)-2-cyclobutan-1-one (**9g**): this product was not detected in the crude reaction mixture (by ^{1}H NMR).

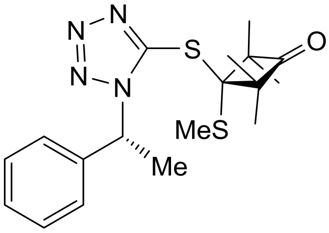

10g

(R)-2,2,4,4-Tetramethyl-3-(methylthio)-3-((1-(1-phenylethyl)-1H-tetrazol-5-yl)thio)cyclobutan-1-one (**10g**): yield 87 mg (46%), colorless crystals; m.p. 117–119 °C (hexane/CH_2Cl_2).

^{1}H NMR ($CDCl_3$): δ 1.45 (*s*, 3H, SMe), 1.50, 1.59, 1.60, 1.73 (4*s*, 12 H, 4Me), 2.035 (d, $^{3}J_{H\text{-}H}$ = 6 Hz, 3H, CH_3), 5.665 (q, $^{3}J_{H\text{-}H}$ = 6 Hz, 1H, *CH*-Me), 7.26–7.36 (*m*, 5H, C_6H_5).
^{13}C NMR ($CDCl_3$): δ 15.3 (S*C*H_3). 20.5, 20.7, 22.0, 23.7, 24.0 (5Me), 58.7, 68.5, 68.6 (2C_q), 74.6 (S–*C*–S), 126.4 (2HC_{ar}), 128.7 (HC_{ar}), 129.0 (2HC_{ar}), 138.9 (C_{ar}), 150.7 (N=*C*–S), 217.1 (*C*=O).
EA for $C_{18}H_{24}N_4OS_2$ (376.54): calc. C 57.41, H 6.42, N 14.88, S 17.03; found C 57.29, H 6.48, N 14.82, S 16.97.

$\alpha^{20}_{CHCl_3} = 18.18$

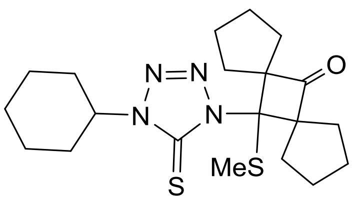

9i

12-(4-Cyclohexyl-5-thioxo-4,5-dihydro-1H-tetrazol-1-yl)-12-(methylthio)dispiro[4.1.4^7.1^5]dodecan-6-one (**9i**): isolated as a less polar fraction, yield: 99 mg (48%), colorless crystals, m.p. 146–148 °C (hexane/CH_2Cl_2).

^{1}H NMR ($CDCl_3$): δ 1.24–1.34 (*m*, 1H), 1.42–1.53 (*m*, 2H), 1.66–1.82 (*m*, 7H), 1.73 (*s*, 3H, SMe), 1.85–1.97 (*m*, 8H), 2.06–2.14 (*m*, 4H), 2.27–2.37 (*m*, 2H), 2.43–2.56 (br. *m*, 2H), 4.66–4.74 (*m*, 1H, N*CH*).

^{13}C NMR ($CDCl_3$): δ 13.5 (S*C*H$_3$). 25.0, 25.1, 26.2, 26.6, 31.1, 31.6 (br), 35.8 (br), 57.5, 74.8 (N−*C*−S), 163.4 (*C*=S), 215.5 (*C*=O).

EA for $C_{20}H_{30}N_4OS_2$ (406.60): calc. C 59.08, H 7.44, N 13.78, S 15.77; found C 59.01, H 7.16, N 13.55, S 15.82.

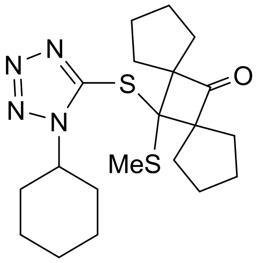

10i

12-((1-Cyclohexyl-1H-tetrazol-5-yl)thio)-12-(methylthio)dispiro[4.1.4^7.1^5]dodecan-6-one (**10i**): isolated as a more polar fraction, yield: 100 mg (49%), colorless crystals, m.p. 121–123 °C (hexane/CH_2Cl_2).

^{1}H NMR ($CDCl_3$): δ 1.28–1.48 (*m*, 4H), 1.71–1.83 (*m*, 9H), 1.92–2.03 (*m*, 8H), 2.04 (*s*, 3H, SMe), 2.05–2.11 (*m*, 2H), 2.24–2.35 (*m*, 4H), 4.27–4.34 (*m*, 1H, N-C*H*).

^{13}C NMR ($CDCl_3$): δ 15.5 (S*C*H$_3$). 24.8, 25,2, 25.4, 25.8, 32.6, 32.8, 35.8, 58.3, 71.7, 78.1 (S−*C*−S), 149.5 (N=*C*–S), 216.0 (*C*=O).

EA for $C_{20}H_{30}N_4OS_2$ (406.60): calc. C 59.08, H 7.44, N 13.78, S 15.77; found C 58.98, H 7.43, N 13.95, S 15.59.

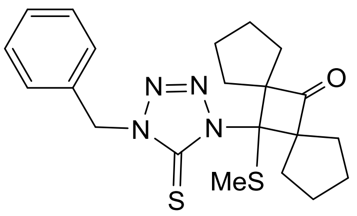

9j

12-(4-Benzyl-5-thioxo-4,5-dihydro-1H-tetrazol-1-yl)-12-(methylthio)dispiro[4.1.4^{7}.1^{5}]dodecan-6-one (**9j**): isolated as a less polar fraction, yield: 95 mg (46%), colorless crystals, m.p. 126–128 °C (hexane/CH_2Cl_2).

^{1}H NMR ($CDCl_3$): δ 1.67–1.75 (*m*, 4H), 1.70 (*s*, 3H, SMe), 1.94–2.05 (*m*, 5H), 2.18–2.29 (*m*, 4H), 5.50 (*s*, 2H, -C*H*$_2$Ph), 7.20–7.39 (*m*, 5H, *H*C$_{ar}$).

^{13}C NMR ($CDCl_3$): δ 13.5 (S*C*H$_3$). 26.1, 26.6, 31.5 (br), 35.7 (br), 51.1, 75.0 (N–*C*–S), 128.6 (2H*C*$_{ar}$), 128.8 (H*C*$_{ar}$), 128.9 (2H*C*$_{ar}$), 133.6 (*C*$_{ar}$), 164.4 (*C*=S), 215.3 (*C*=O).

EA for $C_{21}H_{26}N_4OS_2$ (414.58): calc. C 60.84, H 6.32, N 13.51, S 15.47; found C 60.88, H 6.36, N 13.49, S 15.46.

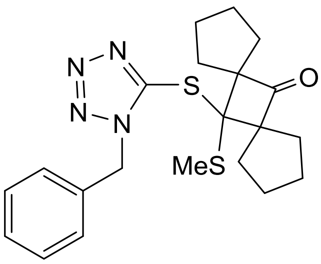

10j

12-((1-Benzyl-1H-tetrazol-5-yl)thio)-12-(methylthio)dispiro[4.1.4^{7}.1^{5}]dodecan-6-one (**10**j); isolated chromatographically, yield: 61 mg (29%), colorless crystals, m.p. 105–107 °C (hexane/CH_2Cl_2).

^{1}H NMR ($CDCl_3$): δ 1.65–1.79 (*m*, 4H), 1.84 (*s*, 3H, SMe), 1.94–2.05 (*m*, 5H), 2.18–2.29 (*m*, 4H), 5.50 (*s*, 2H, -C*H*$_2$Ph), 7.20–7.39 (*m*, 5H, *H*C$_{ar}$).

^{13}C NMR ($CDCl_3$): δ 15.2 (S*C*H$_3$). 25.4, 25.8, 32.7, 35.7, 51.3, 72.0, 78.1 (S–*C*–S), 127.9 (2H*C*$_{ar}$), 128.9 (H*C*$_{ar}$), 129.1 (2H*C*$_{ar}$), 133.1 (*C*$_{ar}$), 150.9 (N=*C*–S), 216.6 (*C*=O).

EA for $C_{21}H_{26}N_4OS_2$ (414.58): calc. C 60.84, H 6.32, N 13.51, S 15.47; found C 60.80, H 6.19, N 13.31, S 15.51.

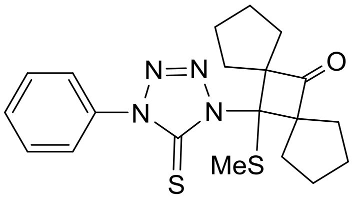

9k

12-(Methylthio)-12-(4-phenyl-5-thioxo-4,5-dihydro-1H-tetrazol-1-yl)dispiro[4.1.4^7.1^5]dodecan-6-one (**9**k). Isolated chromatographically as a less polar fraction, minor fraction, colorless oil, yield: 66 mg (33%). During storage in $CDCl_3$ solution at rt isomerizarion was observed; after 2 h a mixture of ca. 4:1 (**9k**:**10k**) was observed.

^{1}H NMR ($CDCl_3$): δ 1.81 (*s*, SMe).
^{13}C NMR ($CDCl_3$): δ 13.7, 163.4 (*C*=S), 215.3 (*C*=O).

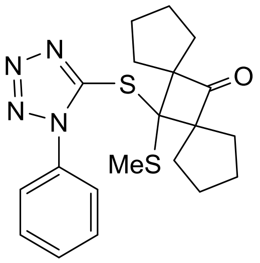

10k

12-(Methylthio)-12-((1-phenyl-1H-tetrazol-5-yl)thio)dispiro[4.1.4^7.1^5]dodecan-6-one (**10k**): isolated chromatographically as a more polar fraction, yield: 126 mg (63 %), colorless crystals, m.p. 128–130 °C (hexane/CH_2Cl_2).

^{1}H NMR ($CDCl_3$): δ 1.70–1.80 (*m*, 8H), 1.82 (*s*, 3H, SMe), 1.95 –2.01 (*m*, 2H), 2.04–2.10 (*m*, 2H), 2.20–2.27 (*m*, 2H), 2.28–2.35 (*m*, 2H), 7.51–7.61 (*m*, 5H, 5HC_{ar}).
^{13}C NMR ($CDCl_3$): δ 15.4 (SCH_3). 25.4, 25.8, 32.5, 35.8 (8CH_2), 72.3 (S−*C*−S), 78.1 (2C_q), 124.5 (2HC_{ar}), 129.7 (2HC_{ar}), 130.4 (HC_{ar}), 133.7 (C_{ar}), 151.0 (N=*C*−S), 216.8 (*C*=O).
EA for $C_{20}H_{24}N_4OS_2$ (400.56): calc. C 59.97, H 6.04, N 13.99, S 16.01; found C 60.03, H 5.98, N 14.02, S 16.13.

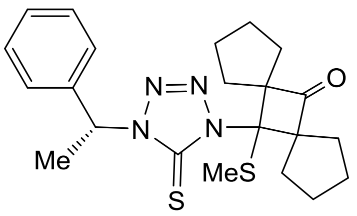

9l

(R)-12-(Methylthio)-12-(4-(1-phenylethyl)-5-thioxo-4,5-dihydro-1H-tetrazol-1-yl)dispiro[4.1.4^{7}.1^{5}]dodecan-6-one (**9l**): isolated chromatographically as a less polar fraction, colorless thick oil, yield: 100 mg (47%).

^{1}H NMR ($CDCl_3$): δ 1.57–1.76 (*m*, 6H), 1.80–1.97 (*m*, 6H), 1.93 (d, $^{3}J_{H,H}$ = 6 Hz, 3H, CH_3), 2.01–2.16 (*m*, 3H), 2.26–2.36 (*m*, 2H), 2.37–2.60 (*m*, 2H), 6.16 (*q*, $^{3}J_{H,H}$ = 6 Hz, 1H, *H*CMe), 7.26–7.38 (*m*, 3H, 3HC_{ar}), 7.41–7.47 (*m*, 2H, 2*H*C_{ar}).
^{13}C NMR ($CDCl_3$): δ 13.4 (SCH_3), 19.9, 25.7, 26.8, 26.1, 26.5 (br), 26.7 (br), 31.5 (br), 33.3, 35.6 (br), 37.2, 56.9, 70.1, 75.0 (N−*C*−S), 126.9 (2HC_{ar}), 128.5 (HC_{ar}), 128.8 (2HC_{ar}), 138.4, 163.8 (*C*=S), 215.3 (*C*=O).
EA for $C_{22}H_{28}N_4OS_2$ (428.61): calc. C 61.65, H 6.58, N 13.07, S 14.96; found C 61.65, H 6.55, N 12.93, S 14.89.

$\alpha^{20}_{CHCl_3}$ = 71.00

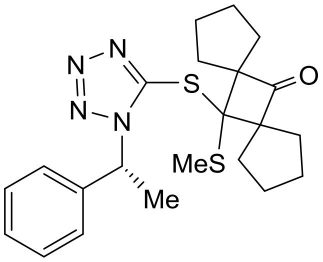

10l

(*R*)-*12-(Methylthio)-12-((1-(1-phenylethyl)-1H-tetrazol-5-yl)thio)dispiro[4.1.4^{7}.1^{5}]dodecan-6-one* (**10l**): colorless crystals, yield: 114 mg (50%), m.p. 127–129 °C (hexane/CH_2Cl_2).

^{1}H NMR ($CDCl_3$): δ 1.68–1.79 (*m*, 8H), 1.78 (*s*, 3H, SMe), 1.92–2.05 (*m*, 4H), 2.035 (*d*, $^{3}J_{H,H}$ = 6 Hz, 3H, -CH-*Me*), 2.12–2.33 (*m*, 4H), 5.675 (*q*, 3JH,H = 6 Hz, -C*H*-Me), 7.26–7.36 (*m*, 5H, 5*H*C_{ar}).

^{13}C NMR (CDCl$_3$): δ 15.1 (S*C*H$_3$). 21.9 (*Me*CH), 25.3, 25.4, 25.7, 25.8 (4CH$_2$), 32.5, 32.7, 35.6, 35.8 (4CH$_2$), 58.7, 71.9, 78.10, 78.13 (S−*C*−S), 126.5 (2H*C*$_{ar}$), 128.7 (H*C*$_{ar}$), 129.0 (2H*C*$_{ar}$), 138.8 (*C*$_{ar}$), 150.5 (N=*C*−S), 216.8 (*C*=O).
EA for $C_{22}H_{28}N_4OS_2$ (428.61): calc. C 61.65, H 6.58, N 13.07, S 14.96; found C 61.59, H 6.61, N 12.92, S 15.00.

$\alpha^{20}_{CHCl_3} = 28.73$

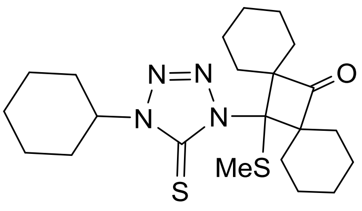

9n

14-(4-Cyclohexyl-5-thioxo-4,5-dihydro-1H-tetrazol-1-yl)-14-(methylthio)dispiro[5.1.5^8.1^6]tetradecan-7-one (**9n**)**;** this product was not found in the crude reaction mixture (by ^{1}H NMR).

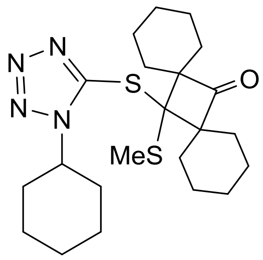

10n

14-((1-Cyclohexyl-1H-tetrazol-5-yl)thio)-14-(methylthio)dispiro[5.1.5^8.1^6]tetradecan-7-one (**10n**): yield 156 mg (72%), colorless crystals, m.p. 170–172 °C (hexane/CH_2Cl_2).

^{1}H NMR (CDCl$_3$): δ 1.14–1.25 (*m*, 2H), 1.28–1.37 (*m*, 1H), 1.39–1.49 (*m*, 2H), 1.63–1.85 (*m*, 15H), 1.92–2.06 (*m*, 6H), 2.00 (*s,* 3H, SMe), 2.35–2.43 (*m*, 4H), 4.27–4.35 (*m*, 1H, N-C*H*).
^{13}C NMR (CDCl$_3$): δ 15.3 (S*C*H$_3$), 23.5, 23.6, 24.8, 25.2, 25.3 (5 signals for 10*C*H$_2$), 31.0, 32.8, 34.0, 58.3 (N-*C*H), 70.8, 75.6 (S–*C*–S), 149.9 (N=*C*–S), 216.0 (*C*=O).
EA for $C_{22}H_{34}N_4OS_2$ (434.66): calc. C 60.79, H 7.88, N 12.89, S 14.75; found C 60.78, H 7.79, N 12.91, S 14.58.

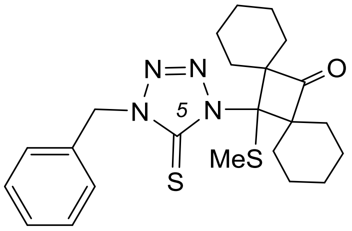

9o

14-(4-Benzyl-5-thioxo-4,5-dihydro-1H-tetrazol-1-yl)-14-(methylthio)dispiro[5.1.5^8.1^6]tetradecan-7-one (**9o**): this product was not found in the crude reaction mixture (by ^{1}H NMR).

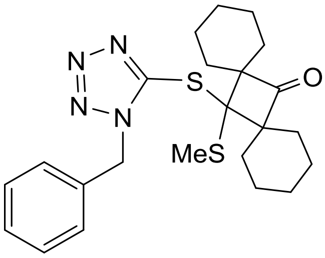

10o

14-((1-Benzyl-1H-tetrazol-5-yl)thio)-14-(methylthio)dispiro[5.1.5^8.1^6]tetradecan-7-one (**10o**). Yield: 115 mg (52 %), m.p. 180–182 °C (hexane/CH_2Cl_2).

^{1}H NMR ($CDCl_3$): δ 1.12–1.23 (*m*, 2H), 1.61–1.83 (*m*, 12H), 1.81 (*s*, 3H, SMe), 1.90–1.99 (*m*, 2H), 2.29–2.41 (*m*, 4H), 5.50 (*s*, 2H, C*H$_2$*Ph), 7.25–7.38 (*m*, 5H, C_6H_5).
^{13}C NMR ($CDCl_3$): δ 15.1 (S*C*H$_3$), 23.5, 23.6, 25.2, 31.0, 33.9 (10*C*H$_2$), 51.2 (Ph*C*H$_2$), 70.8 (2*C*$_q$), 75.9 (S-*C*-S), 127.9 (2H*C*$_{ar}$), , 128.9 (H*C*$_{ar}$), 129.1 (2H*C*$_{ar}$), 133.3 (*C*$_{ar}$), 151.2 (N=*C*–S), 215.8 (*C*=O).
EA for $C_{23}H_{30}N_4OS_2$ (442.64): calc. C 62.41, H 6.83, N 12.66, S 14.49; found C 62.36, H 6.75, N 12.64, S 14.42.

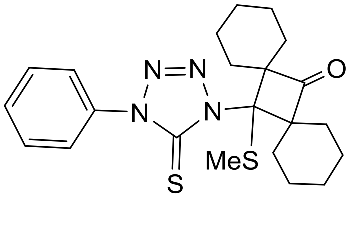

9p

14-(Methylthio)-14-(4-phenyl-5-thioxo-4,5-dihydro-1H-tetrazol-1-yl)dispiro[5.1.5⁸.1⁶]tetradecan-7-one (**9p**). This product was not found in the crude reaction mixture (according to ^{1}H NMR).

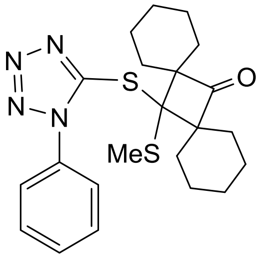

10p

14-(Methylthio)-14-((1-phenyl-1H-tetrazol-5-yl)thio)dispiro[5.1.5⁸.1⁶]tetradecan-7-one (**10p**). Yield: 190 mg (89%), m.p. 186–188 °C (hexane/CH_2Cl_2).

^{1}H NMR ($CDCl_3$): δ 1.12–1.22 (*m*, 2H), 1.61–1.72 (*m*, 10H), 1.74–1.84 (*m*, 2H), 1.76 (*s*, 3H, SMe), 1.97–2.05 (*m*, 2H), 2.33–2.40 (*m*, 4H), 7.51–7.62 (*m*, 5H, C_6H_5).
^{13}C NMR ($CDCl_3$): δ 15.3 (S*C*H_3), 23.5, 23.6, 25.3, 30.9, 34.1 (10*C*H_2), 70.8 (2C_q), 76.3 (S–*C*–S), 124.6 (2HC_{ar}), 129.7 (2HC_{ar}), 130.4 (HC_{ar}), 133.8 (C_{ar}), 151,3 (N=*C*–S), 215.9 (*C*=O).
EA for $C_{22}H_{28}N_4OS_2$ (428.61): calc. C 61.65, H 6.58, N 13.07, S 14.96; found C 61.65, H 6.50, N 13.02, S 15.10.

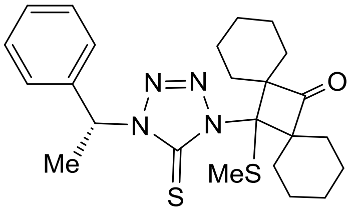

9q

(R)-14-(Methylthio)-14-(4-(1-phenylethyl)-5-thioxo-4,5-dihydro-1H-tetrazol-1-yl)dispiro[5.1.5⁸.1⁶]tetradecan-7-one (**9q**). This product was not found in the crude reaction mixture (by ^{1}H NMR).

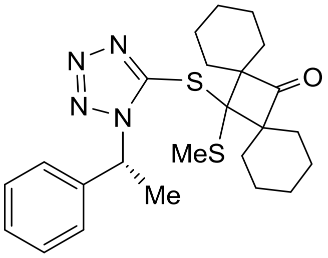

10q

(R)-14-(Methylthio)-14-((1-(1-phenylethyl)-1H-tetrazol-5-yl)thio)dispiro[5.1.5^{8}.1^{6}]tetradecan-7-one (**10q**). Yield: 176 mg (77%), m.p. 149–152 °C (hexane/CH_2Cl_2).

^{1}H NMR ($CDCl_3$): δ 1.13–1.21 (*m*, 2H), 1.40–1.48 (*m*, 2H), 1.59–1.91 (*m*, 22H), 1.73 (*s*, 3H, SMe), 1.96–2.04 (*m*, 1H), 2.025 (*d*, 3H, $^{3}J_{H,H}$ = 6 Hz, -HC*Me*), 2.25–2.35 (*m*, 3H), 2.38–2.44 (*m*, 1H), 5.66 (*q*, 1H, $^{3}J_{H,H}$ = 6 Hz, -*H*CMe), 7.26–7.37 (*m*, 5H, C_6H_5).
^{13}C NMR ($CDCl_3$): δ 14.9 (S*C*H_3), 21.9 (*Me*CH), 22.0, 23.5, 23.6, 23.62, 23.64, 25.2, 25.3, 28.9, 30.8, 31.2, 33.9, 34.0 (10 *C*H_2), 58.6, 70.7, 70.8, 75.8 (S−*C*−S), 126.5 (2HC_{ar}), 128.7 (HC_{ar}), 129.0 (2HC_{ar}), 139.0 (C_{ar}), 150.5 (N=*C*–S), 215.9 (*C*=O).
EA for $C_{24}H_{32}N_4OS_2$ (456.66): calc. C 63.12, H 7.06, N 12.27, S 14.04; found C 63.15, H 7.16, N 12.16, S 13.97.

$\alpha^{20}_{CHCl_3} = 42.12$

Section 3: *^{1}H and ^{13}C NMR spectra registered for thioaminals* **9** *and dithioacetals* **10**

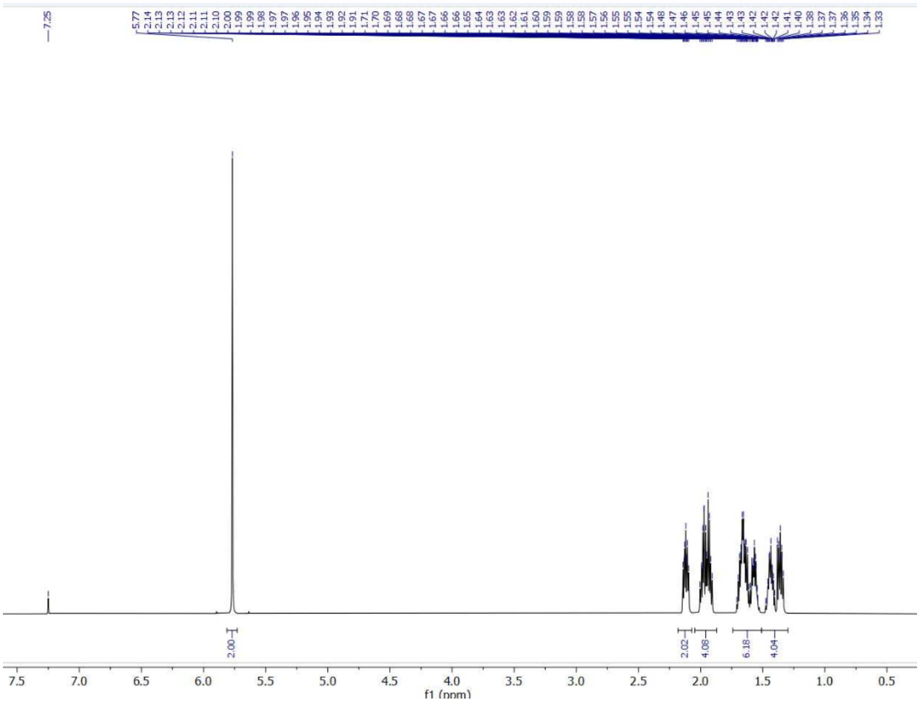

Fig. S1a. The ^{1}H NMR spectrum of **2c**.

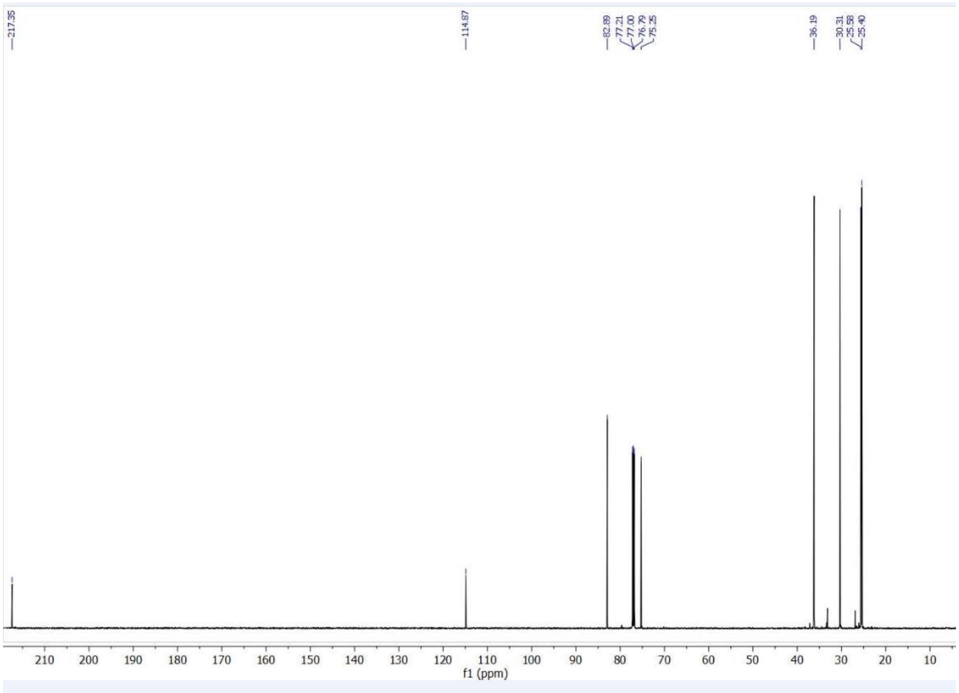

Fig. S1b. The ^{13}C NMR spectrum of **2c.**

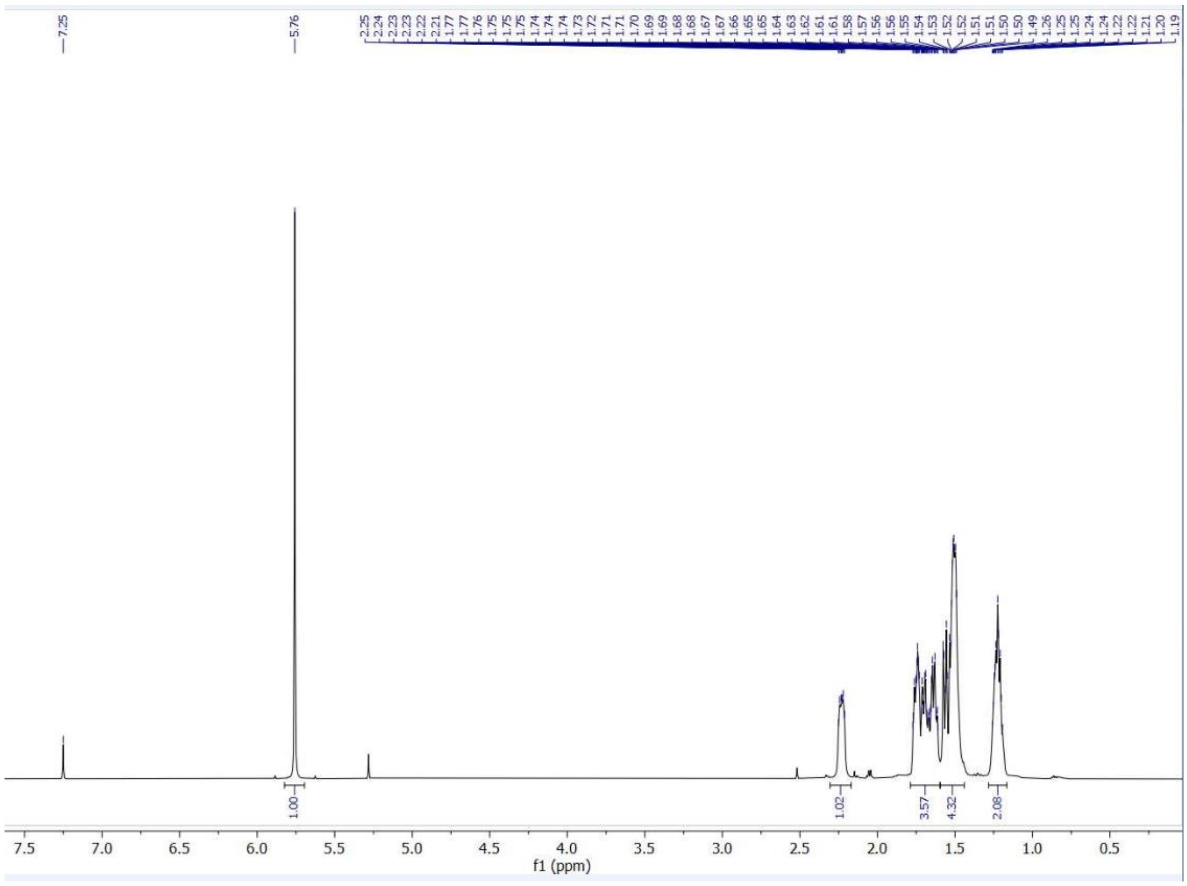

Fig. S2a. The ^{1}H NMR spectrum of **2d**.

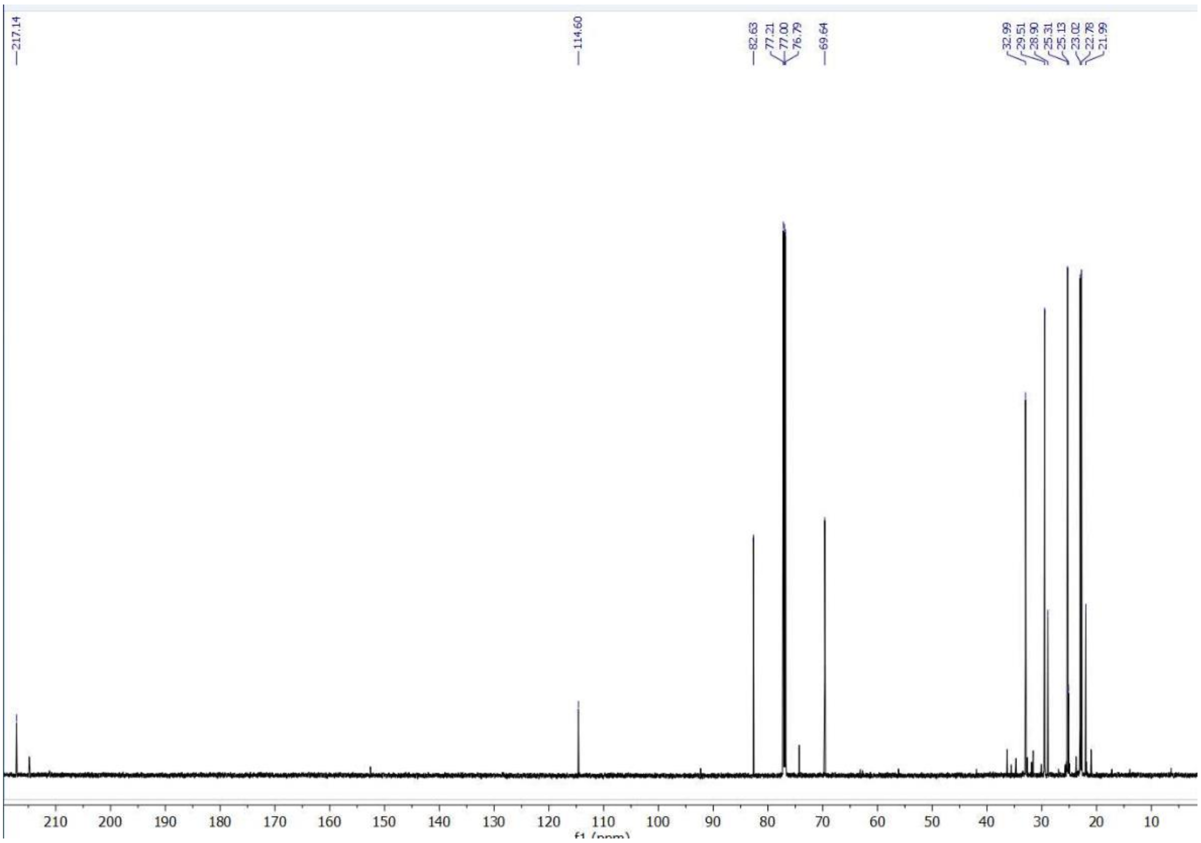

Fig. S2b. The ^{13}C NMR spectrum of **2d.**

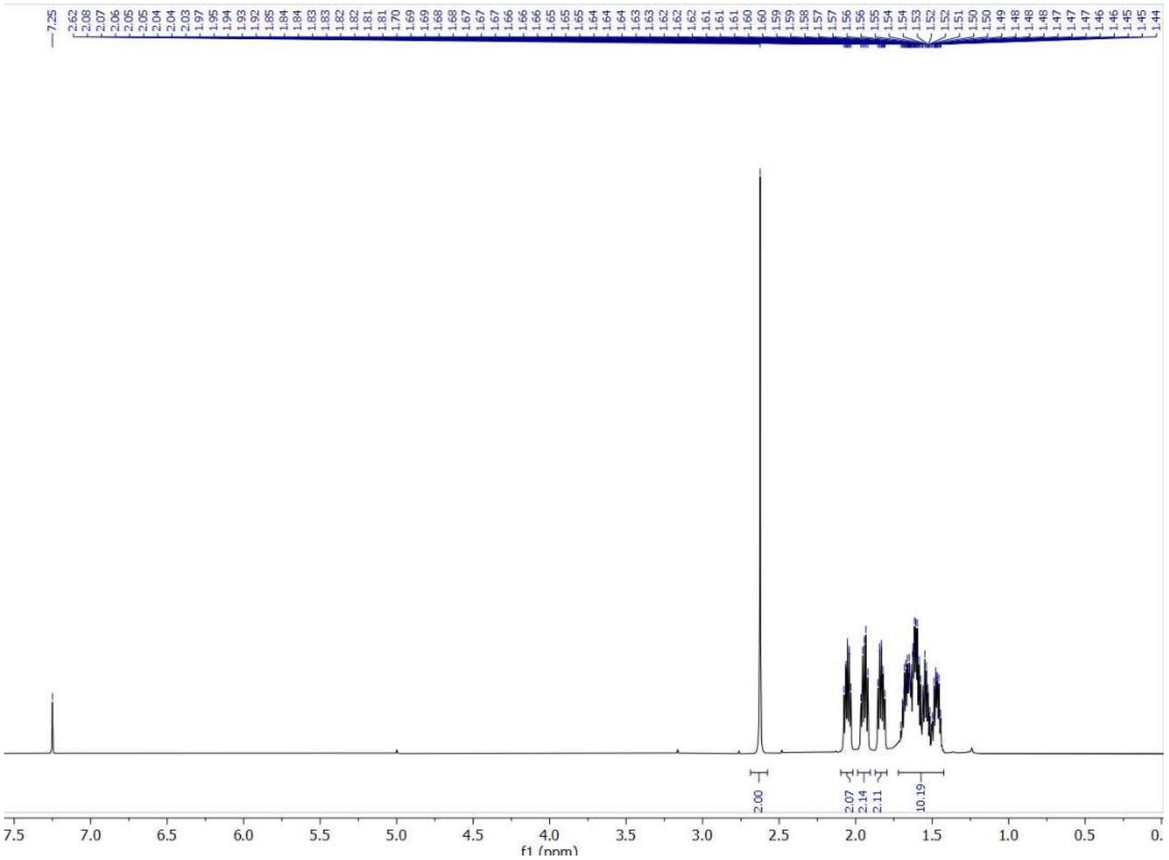

Fig. S3a. The ^{1}H NMR spectrum of **8a.**

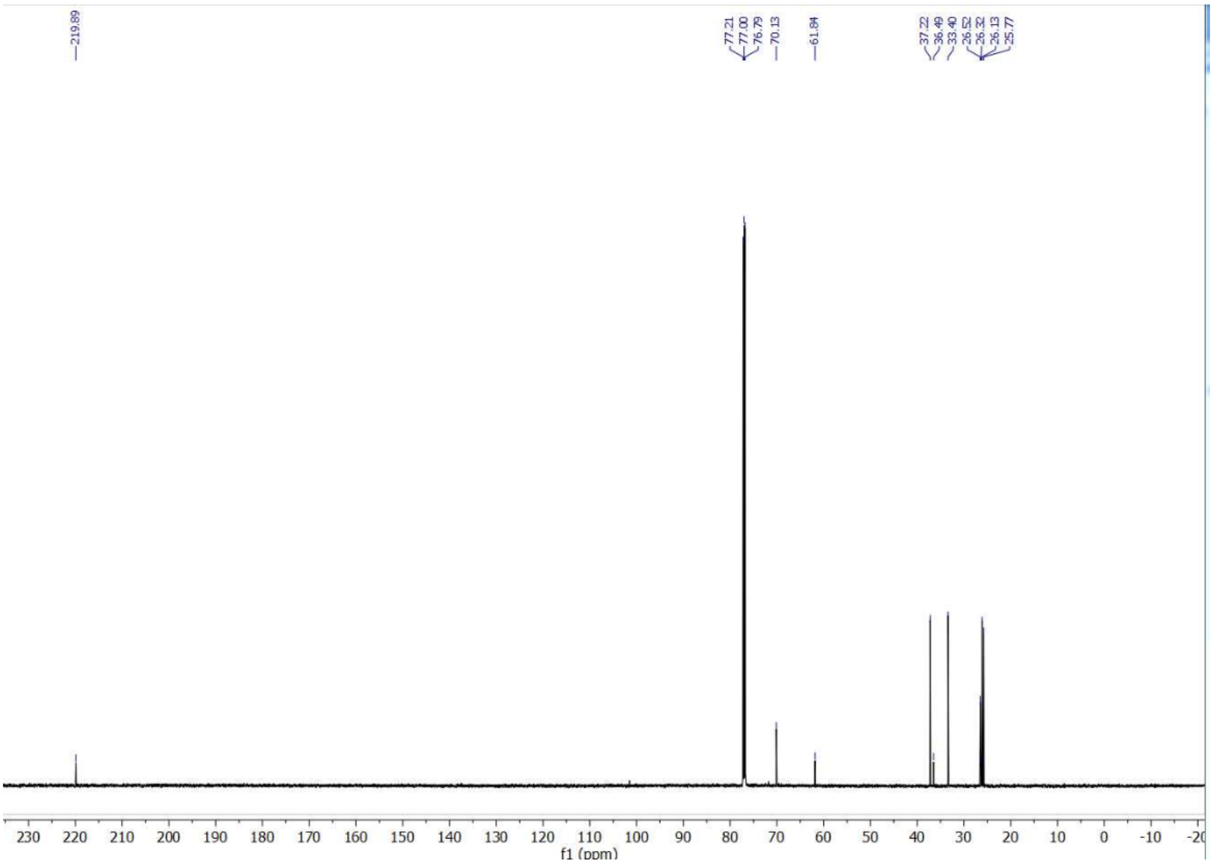

Fig. S3b. The ^{13}C NMR spectrum of **8a**.

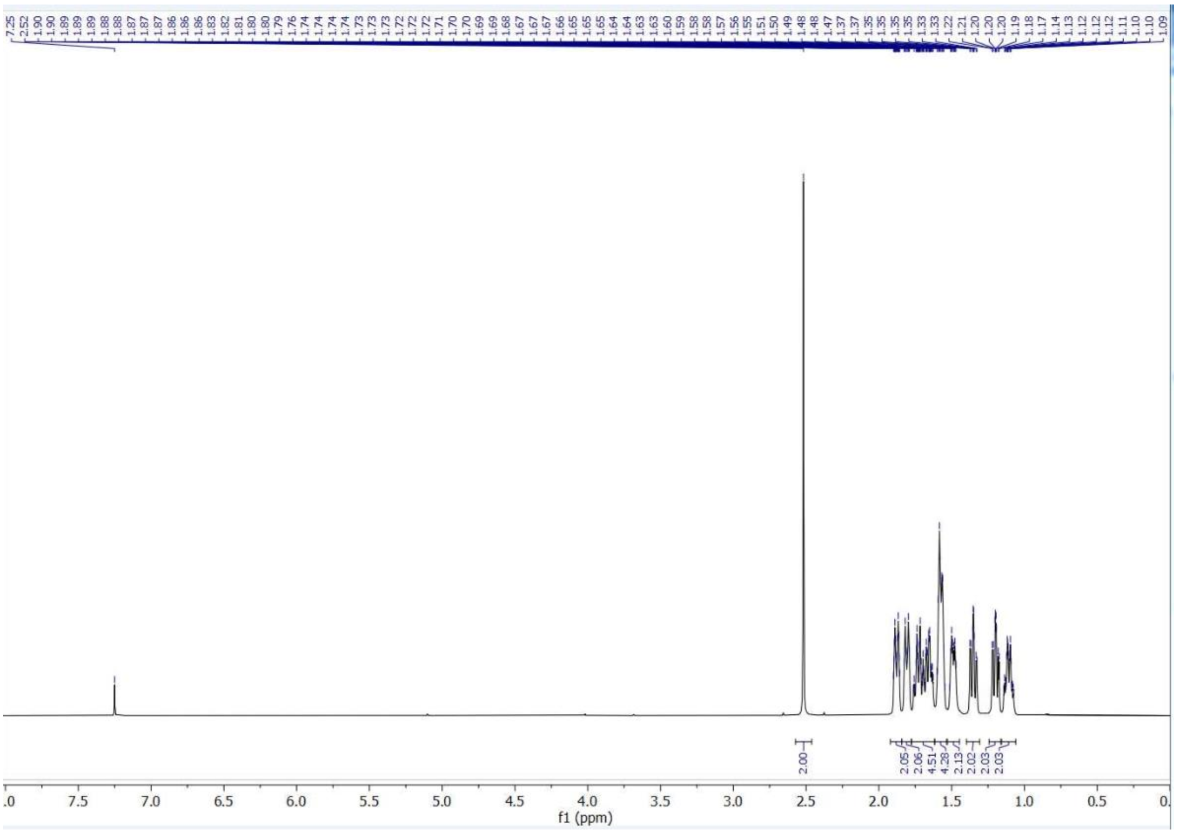

Fig. S4a. The ^{1}H NMR spectrum of **8b**.

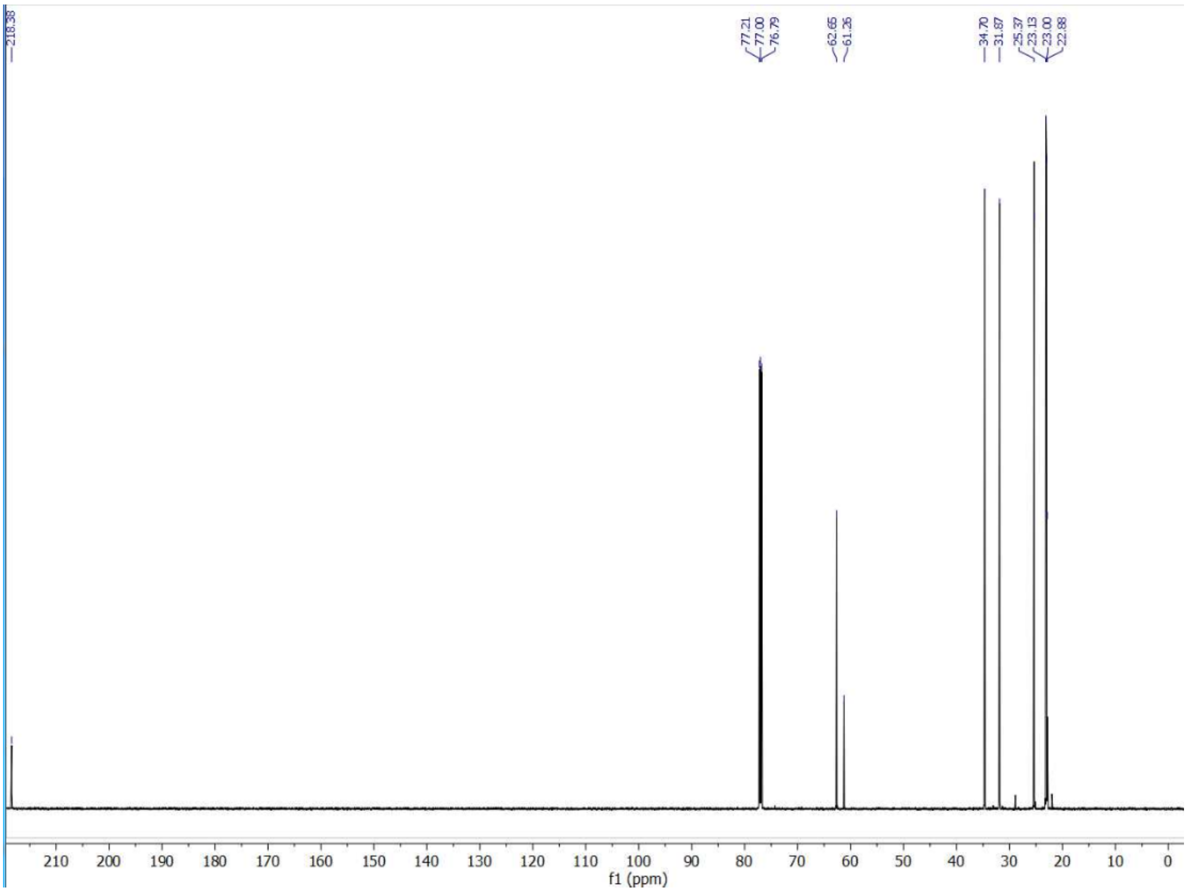

Fig. S4b. The ^{13}C NMR spectrum of **8b**.

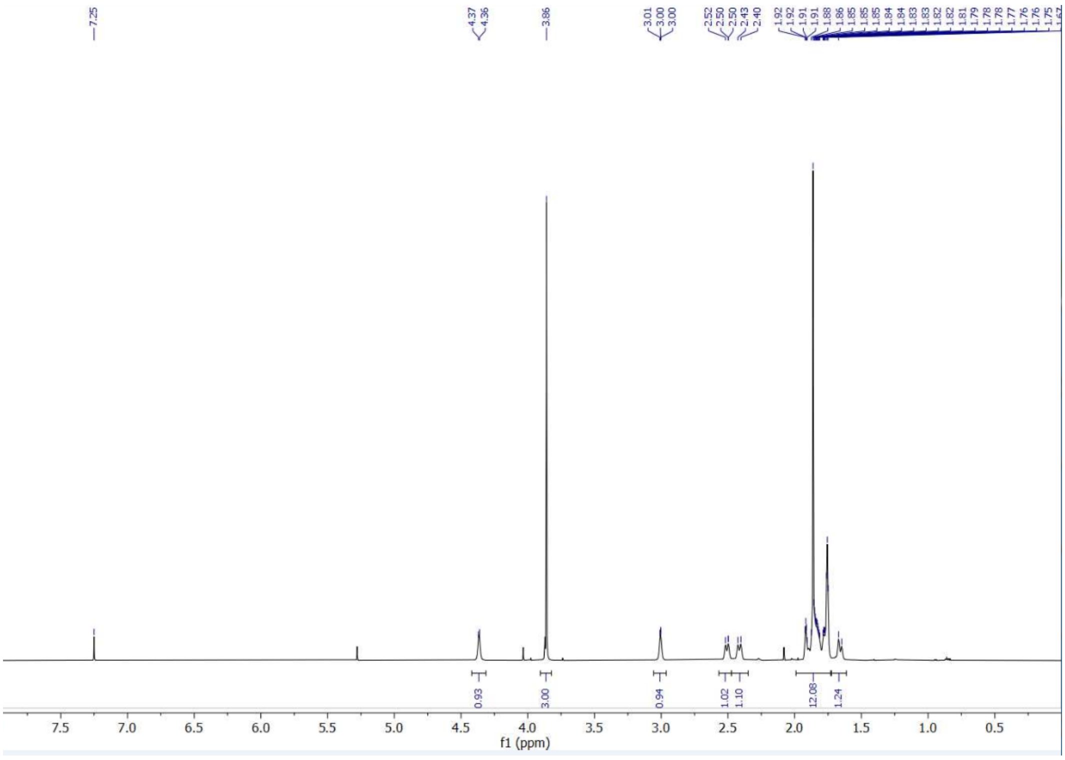

Fig. S5a. The ^{1}H NMR spectrum of **9a**.

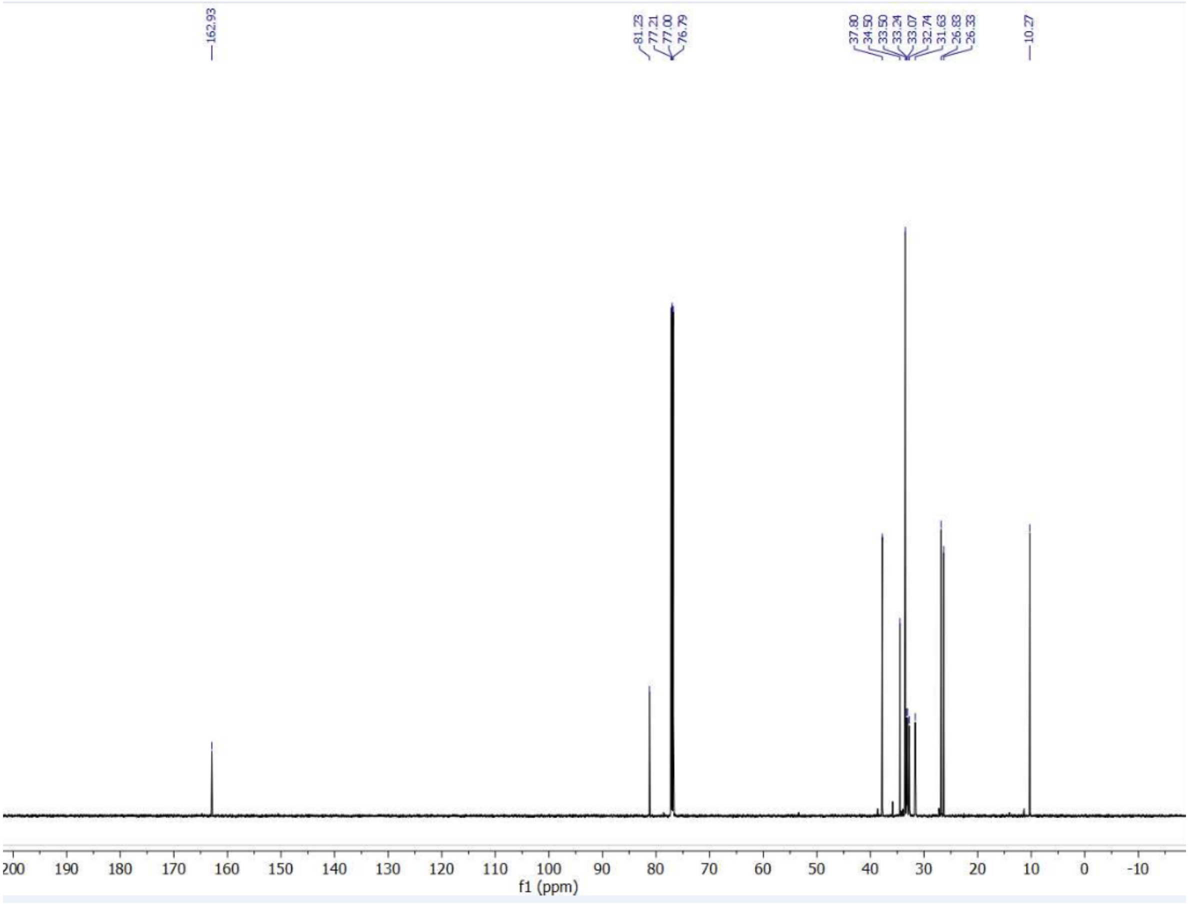

Fig. S5b. The ^{13}C NMR spectrum of **9a**.

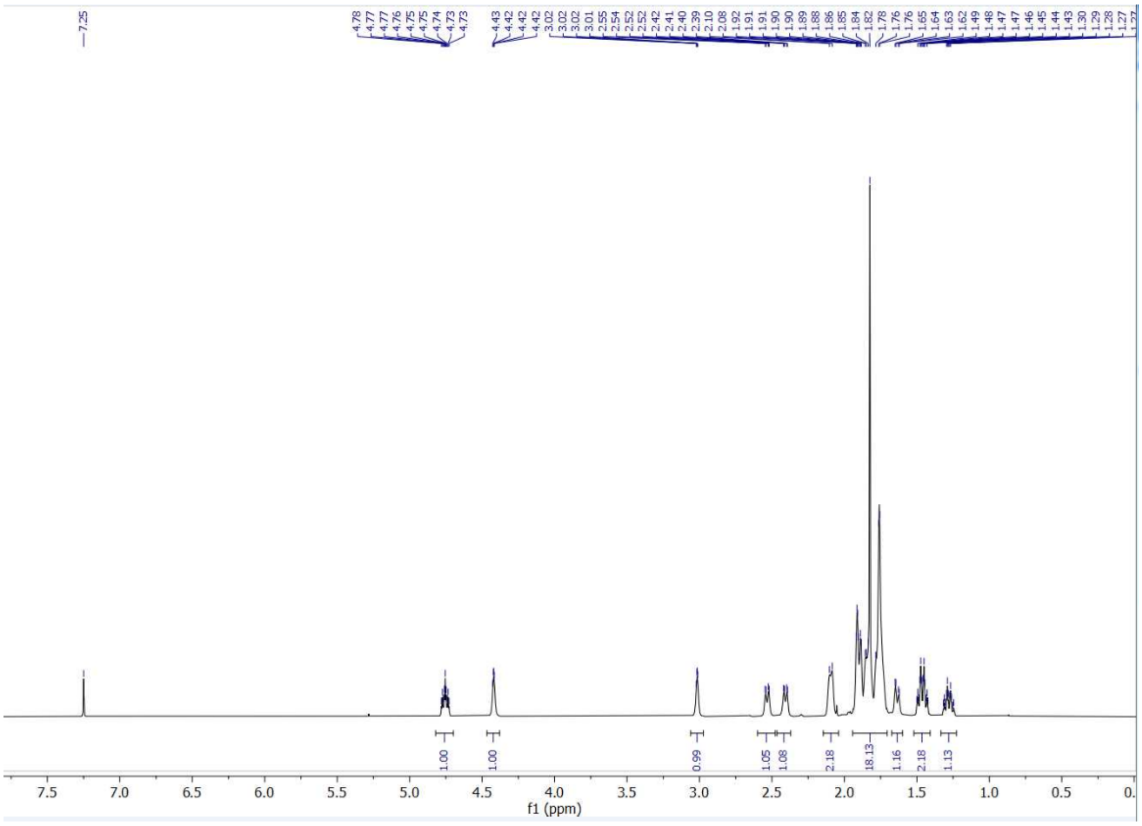

Fig. S6a. The ^{1}H NMR spectrum of **9b**.

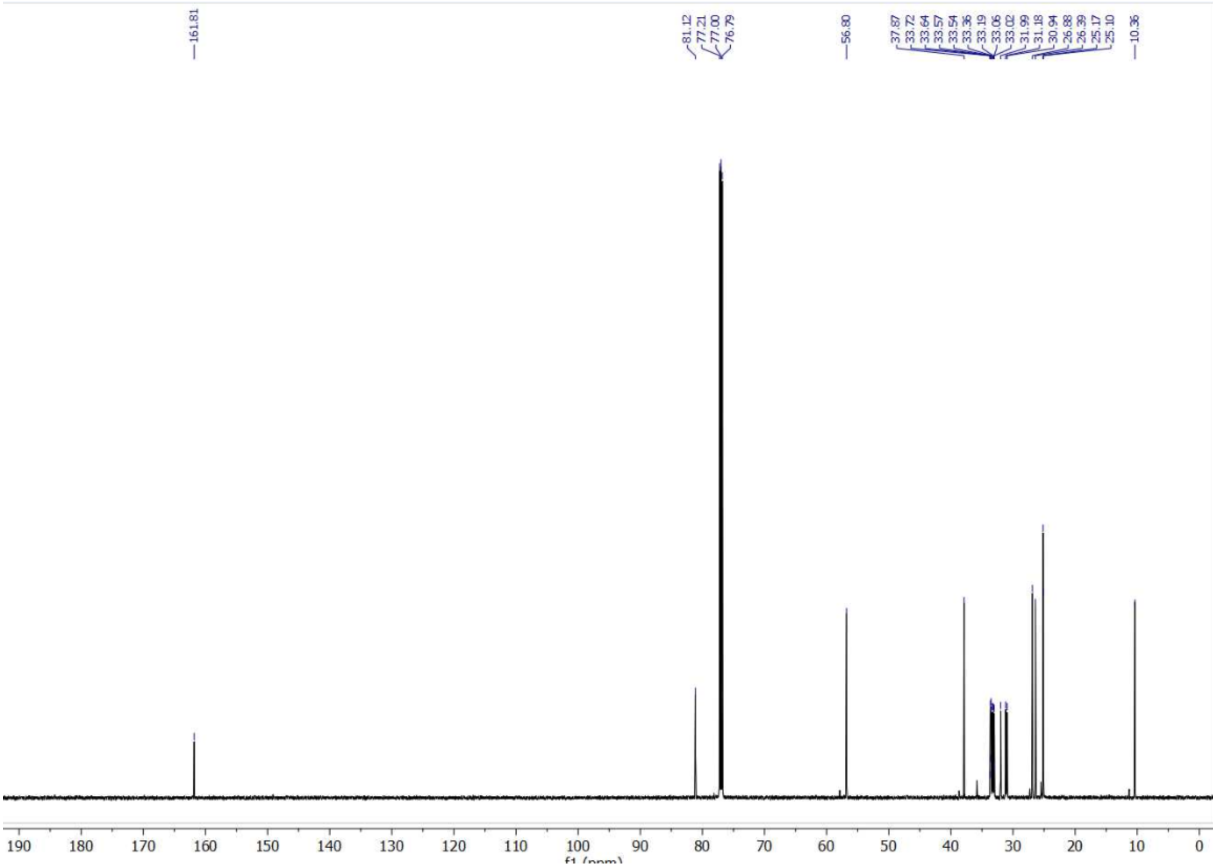

Fig. S6b. The ^{13}C NMR spectrum of **9b**.

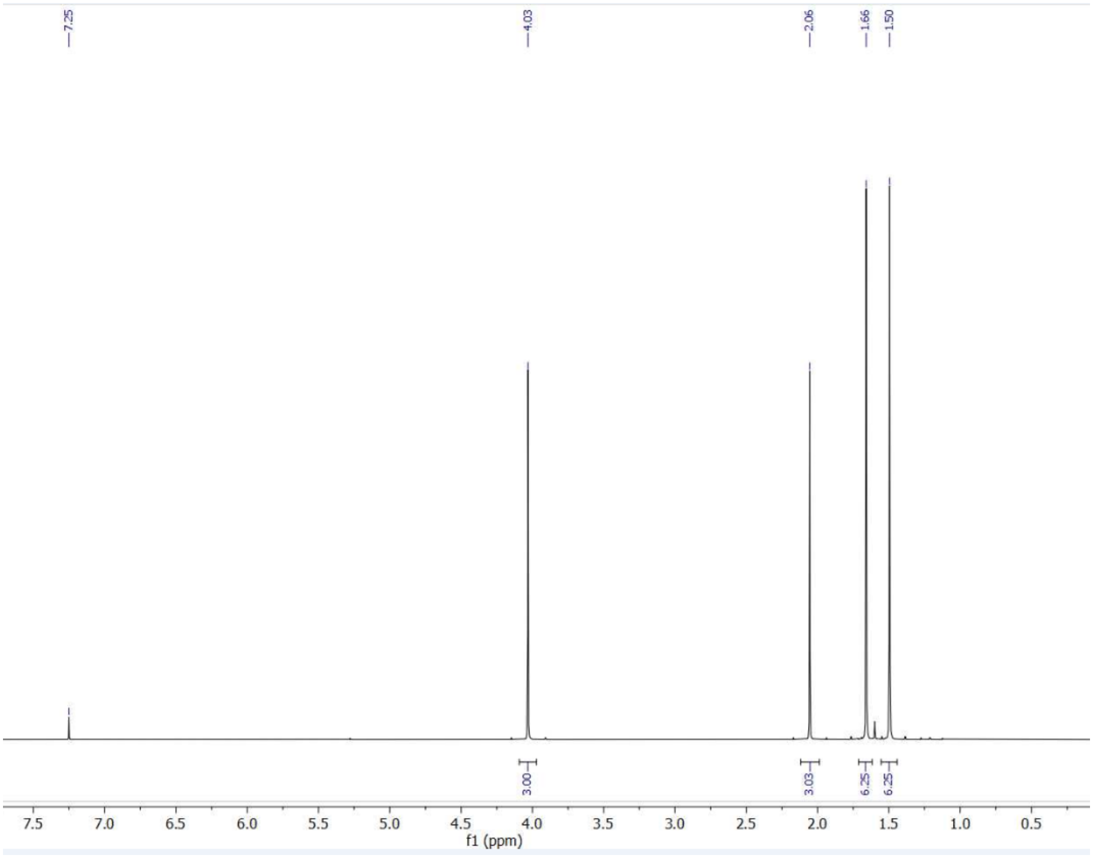

Fig. S7a. The ^{1}H NMR spectrum of **10c**.

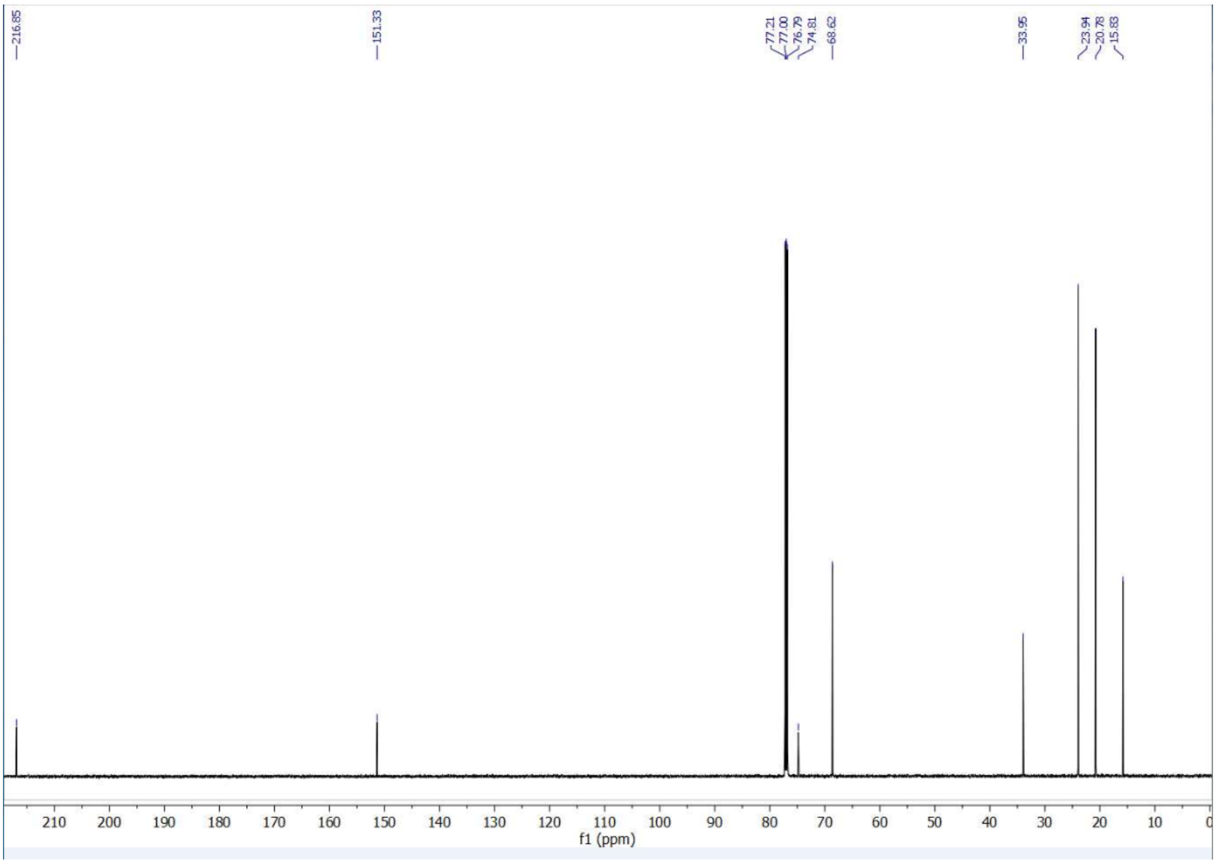

Fig. S7b. The ^{13}C NMR spectrum of **10c**.

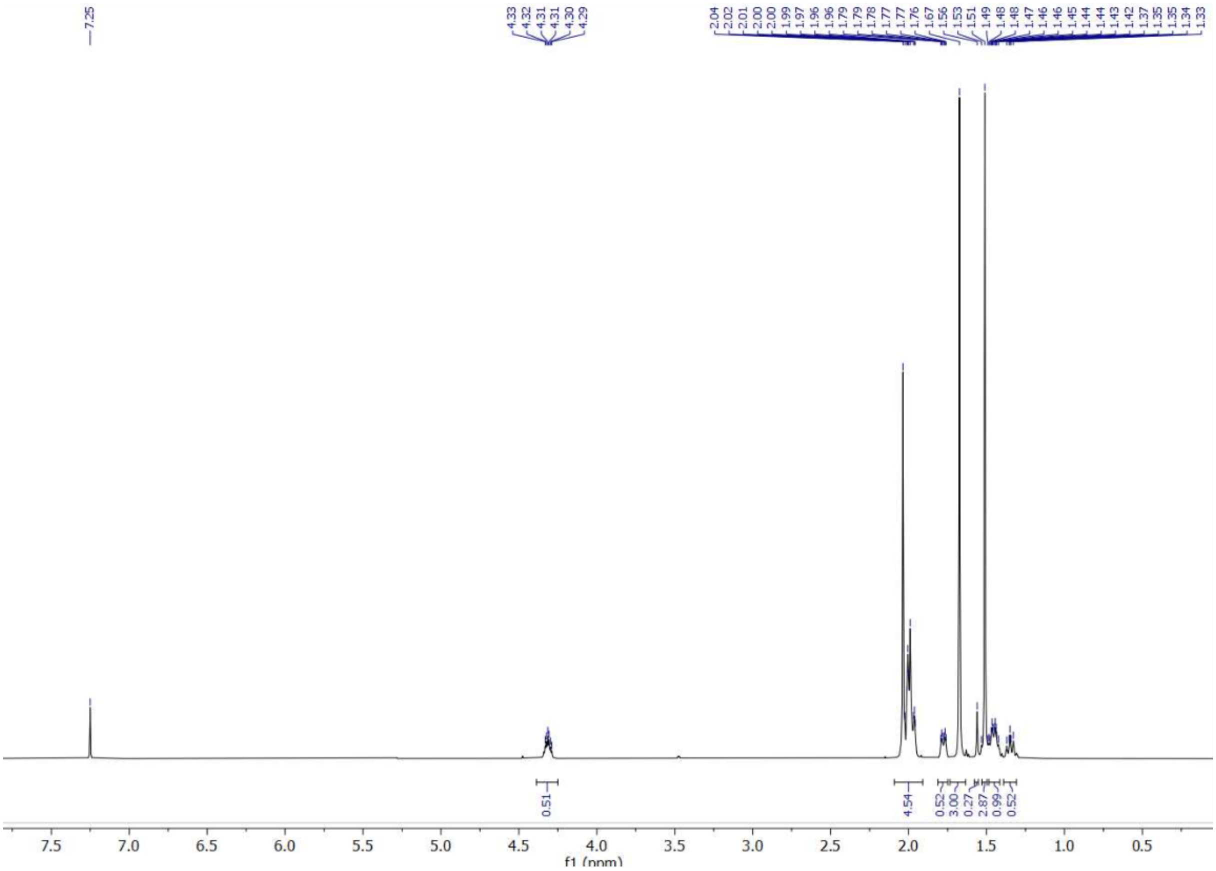

Fig. S8a. The ^{1}H NMR spectrum of **10d**.

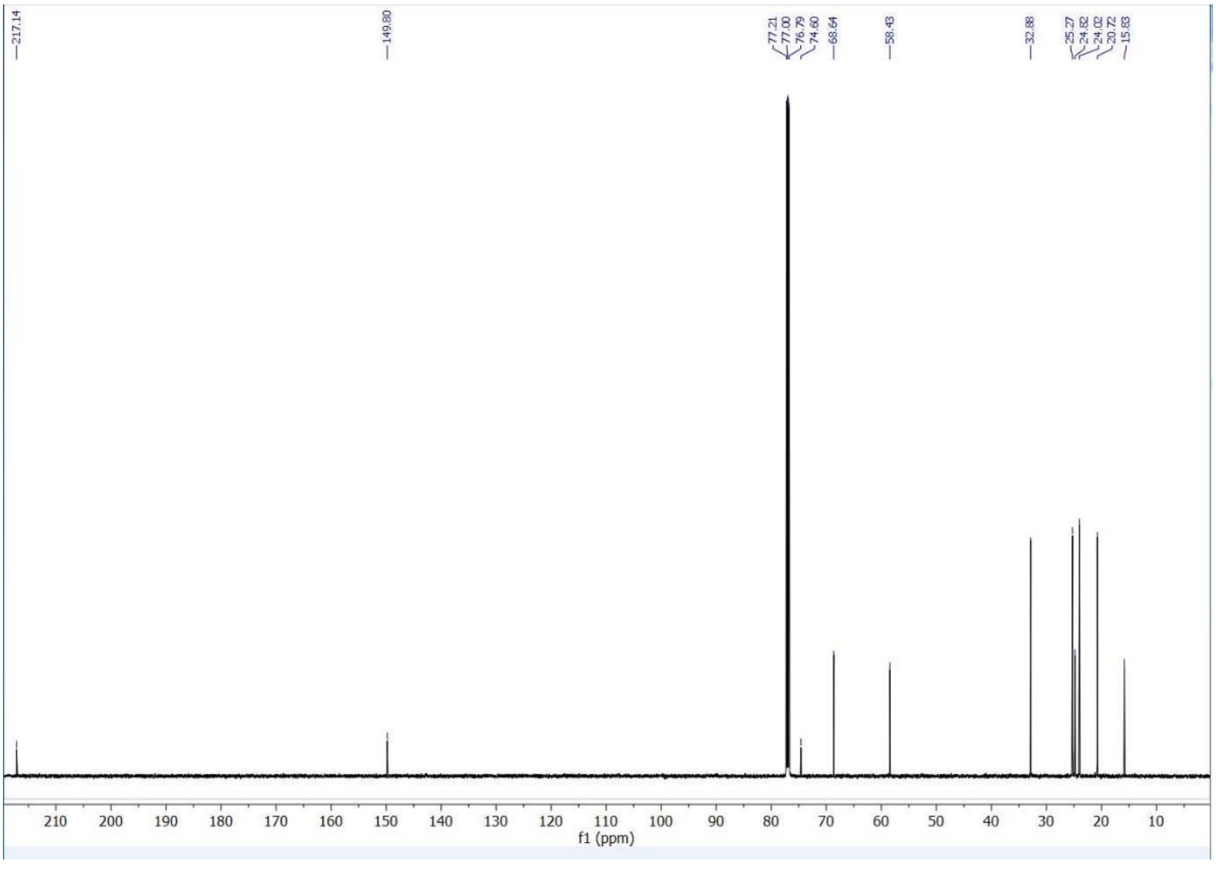

Fig. S8b. The ^{13}C NMR spectrum of **10d**.

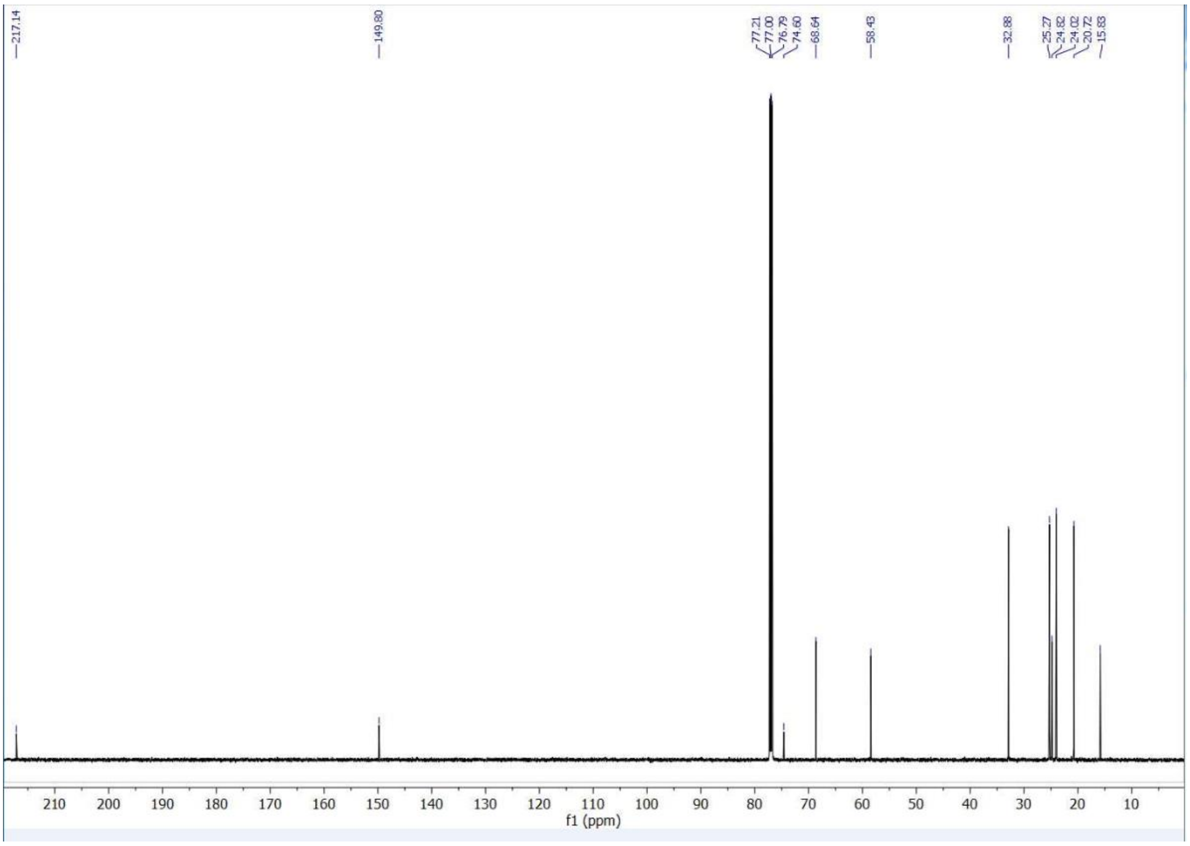

Fig. S9a. The ^{1}H NMR spectrum of **10e**.

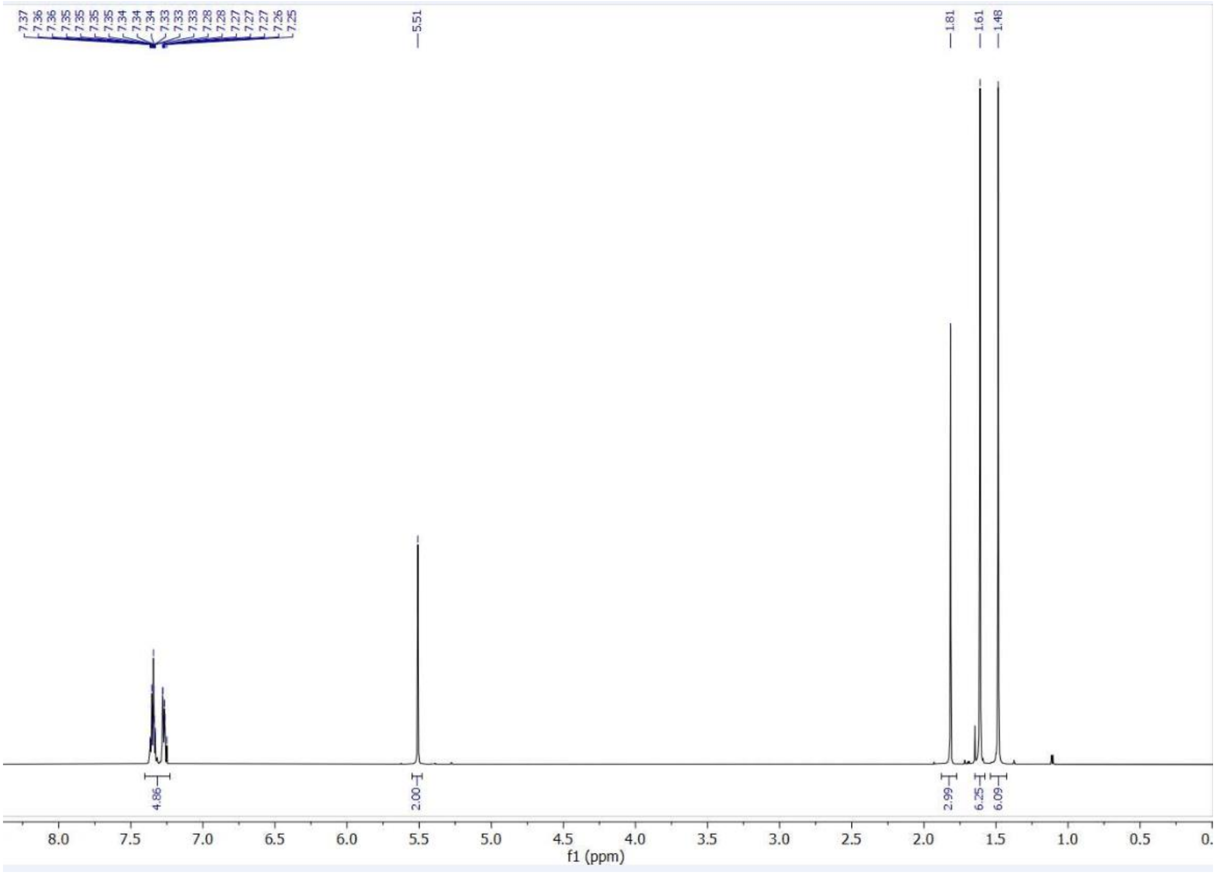

Fig. S9b. The ^{13}C NMR spectrum of **10e**.

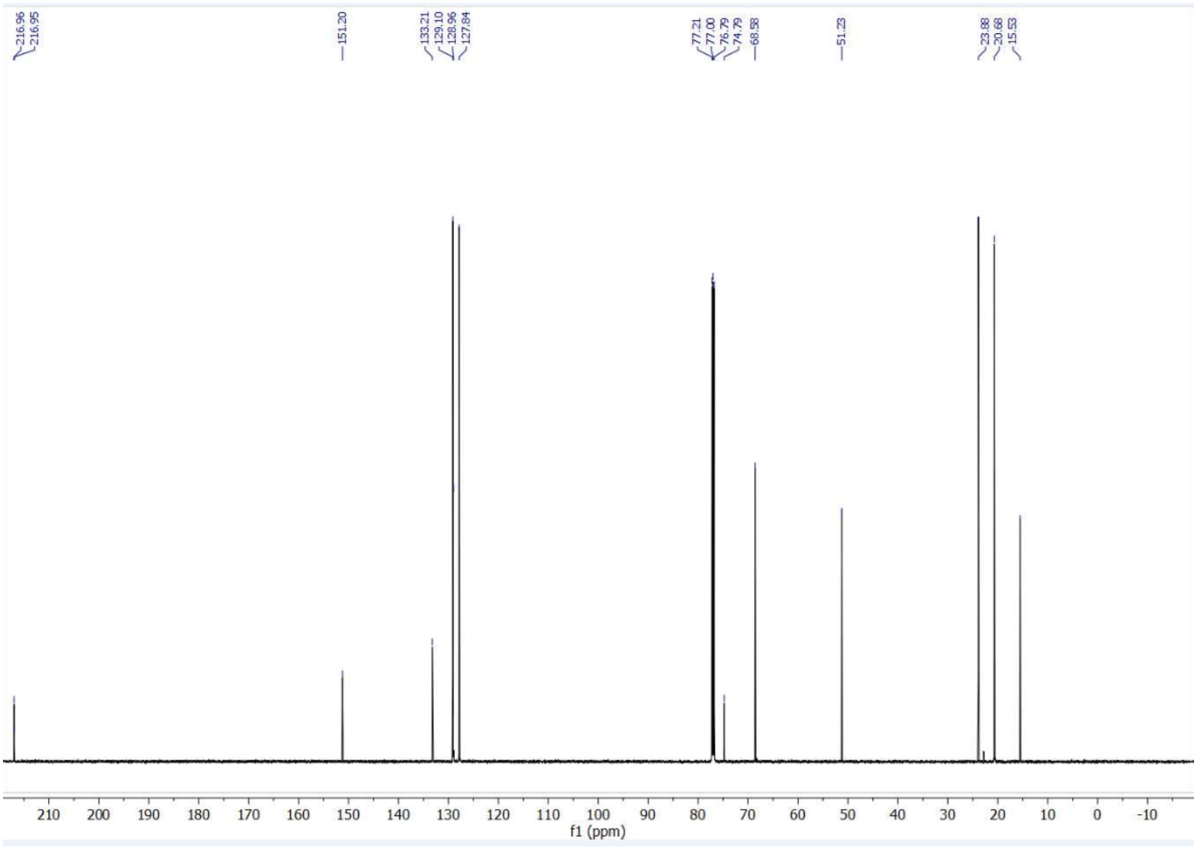

Fig. S10a. The ^{1}H NMR spectrum of **10f**.

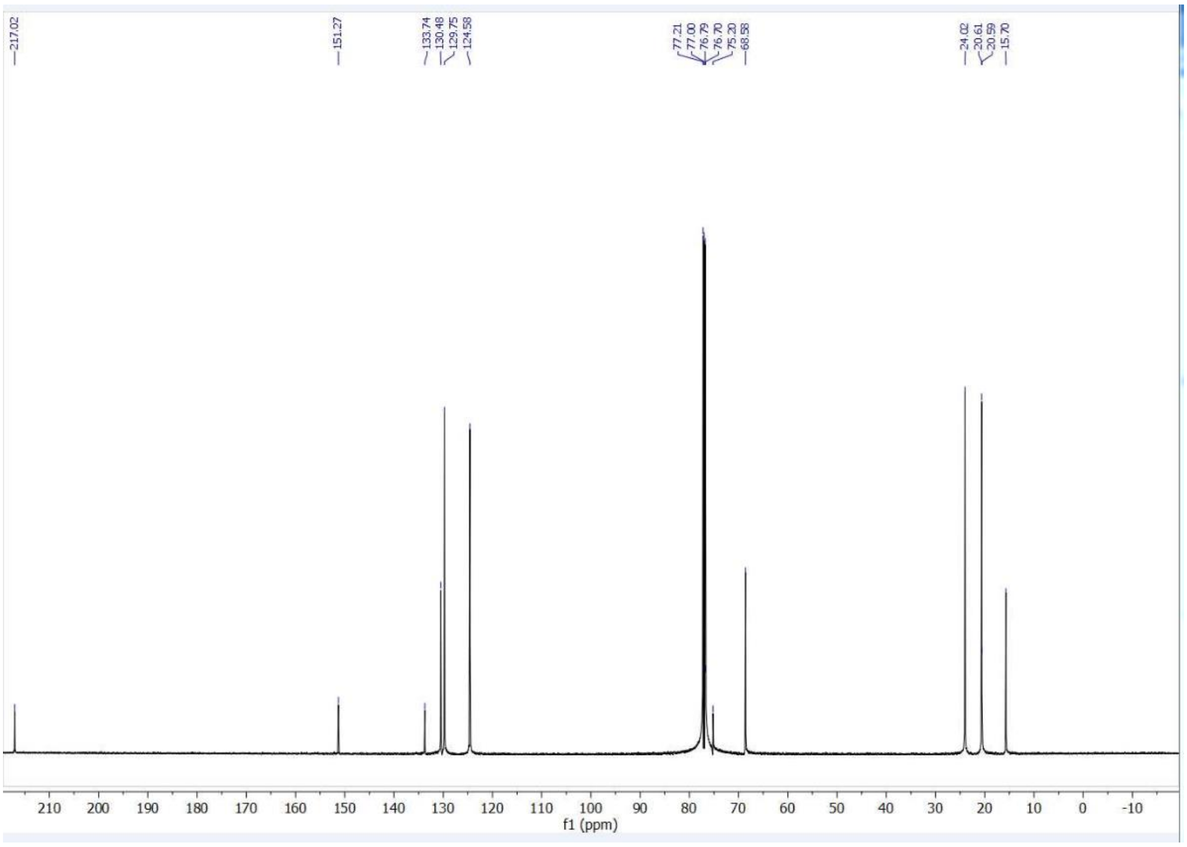

Fig. S10b. The ^{13}C NMR spectrum of **10f**.

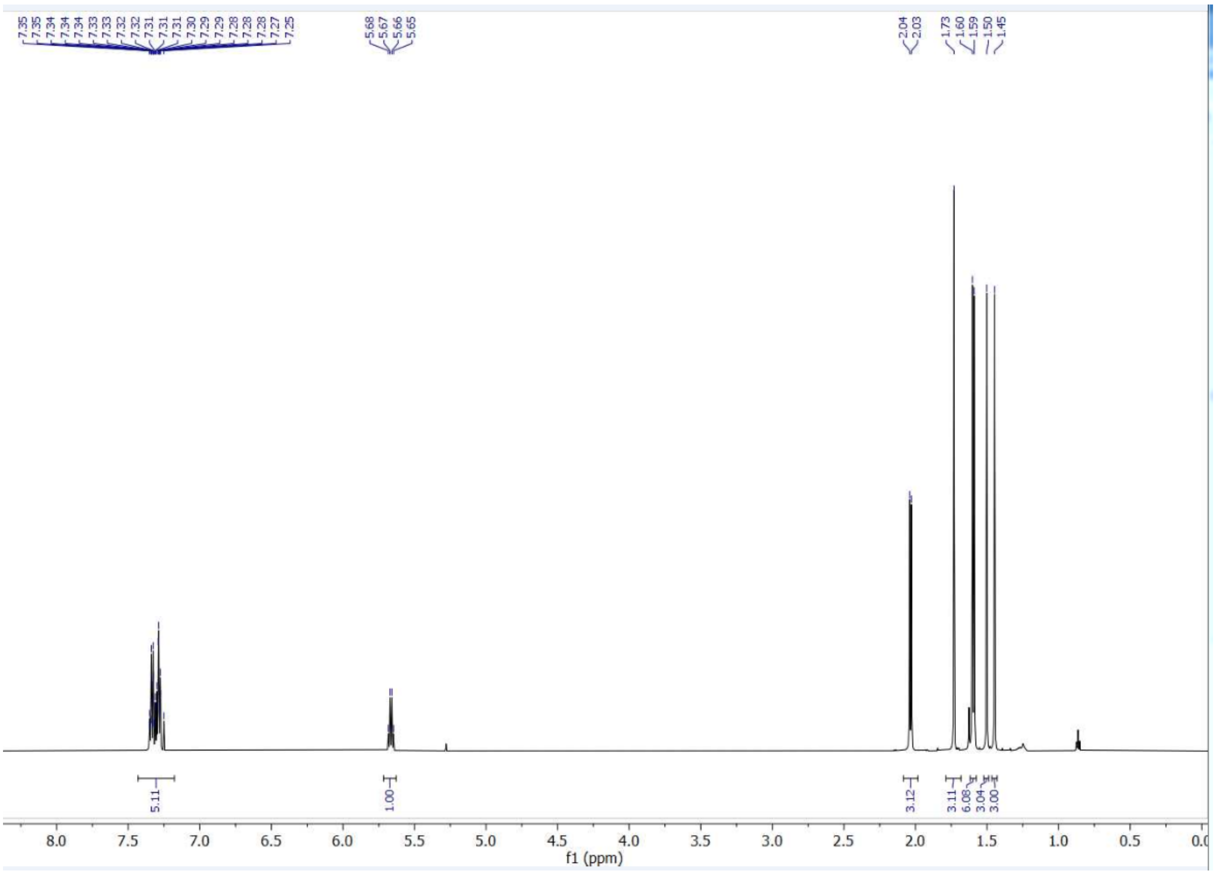

Fig. S11a. The ^{1}H NMR spectrum of **10g**.

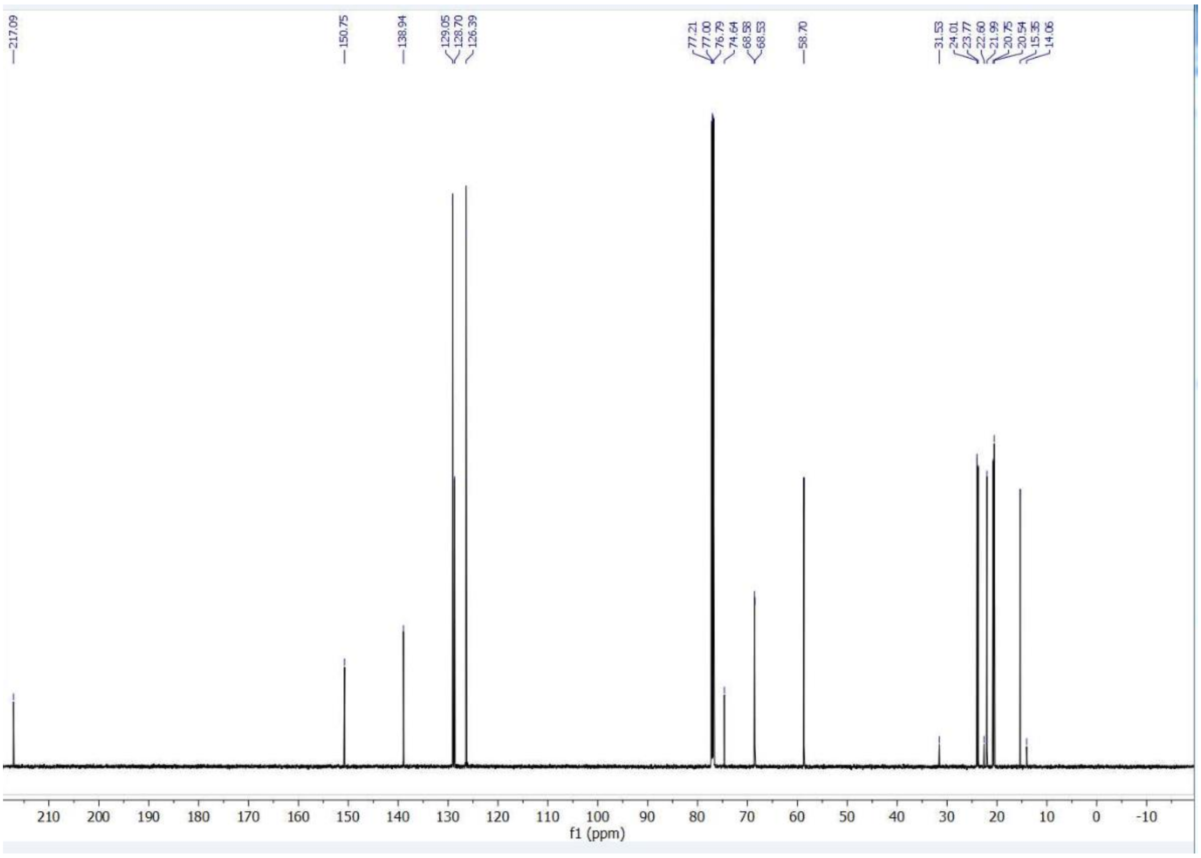

Fig. S11b. The ^{13}C NMR spectrum of **10g**.

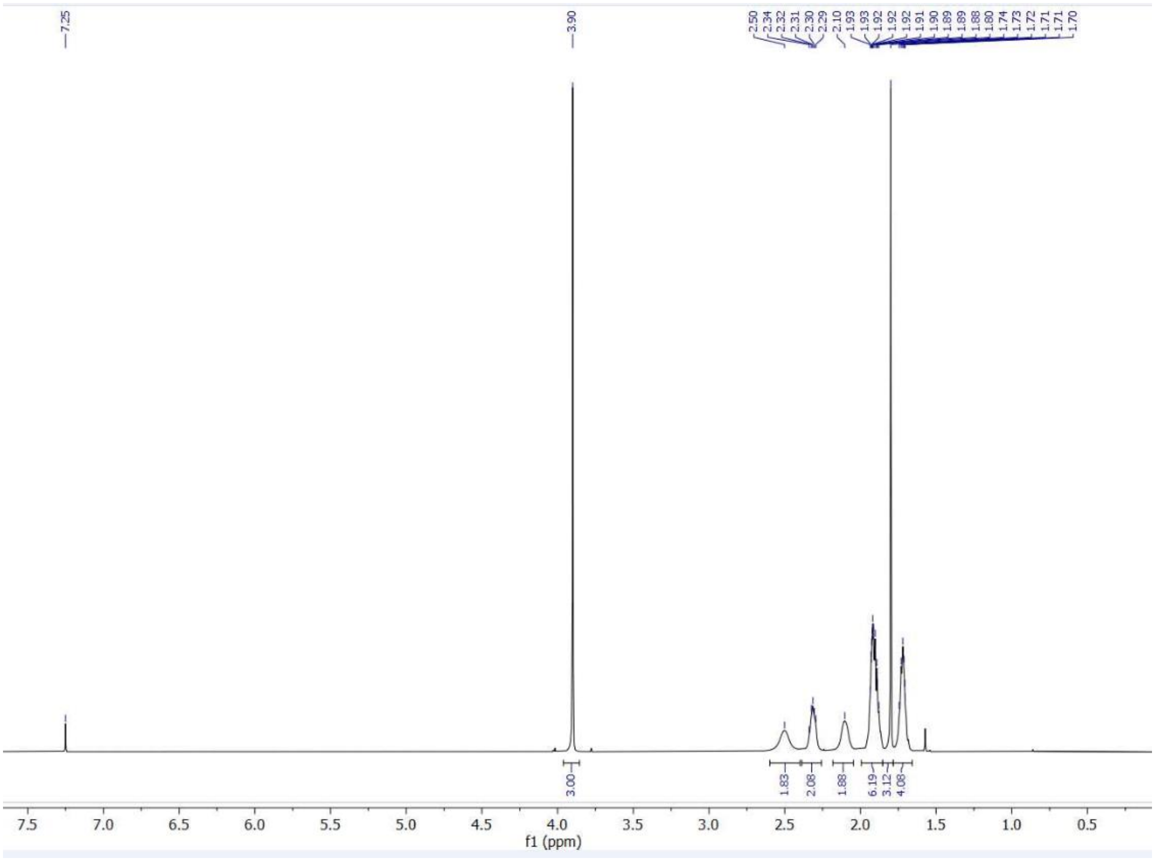

Fig. S12a. The ^{1}H NMR spectrum of **9h**.

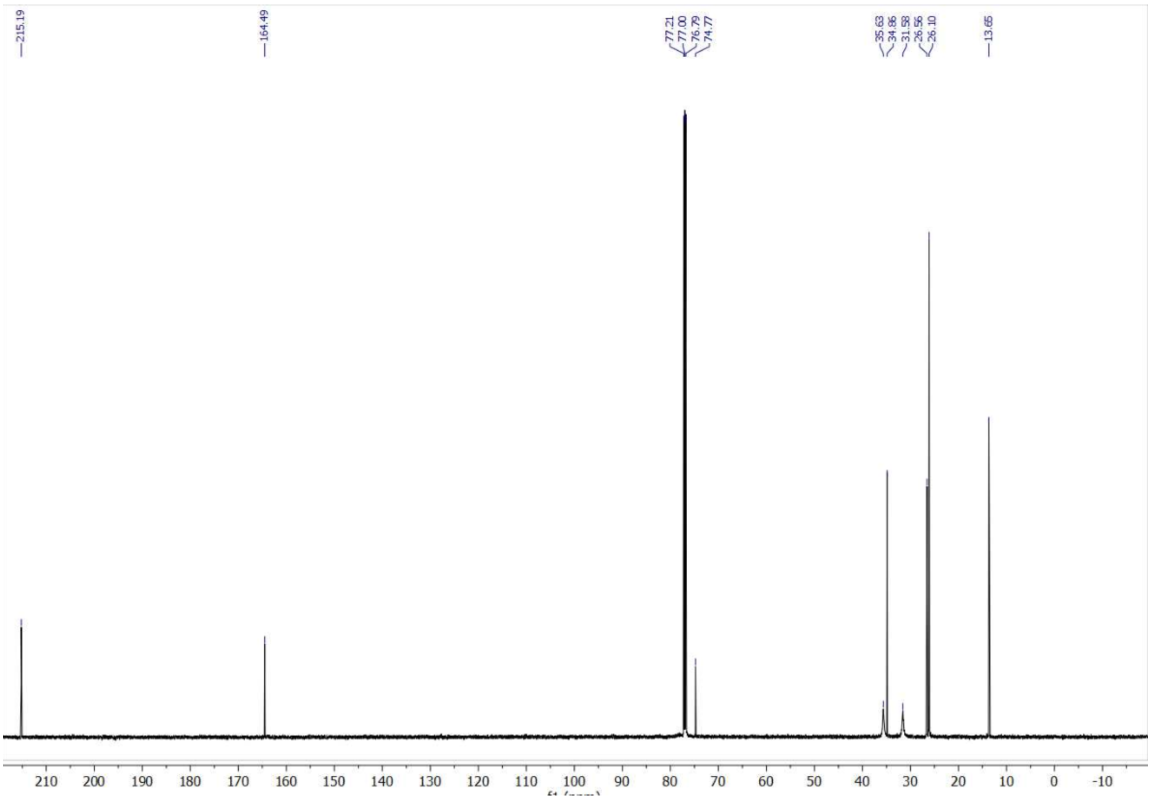

Fig. S12b. The ^{13}C NMR spectrum of **9h**.

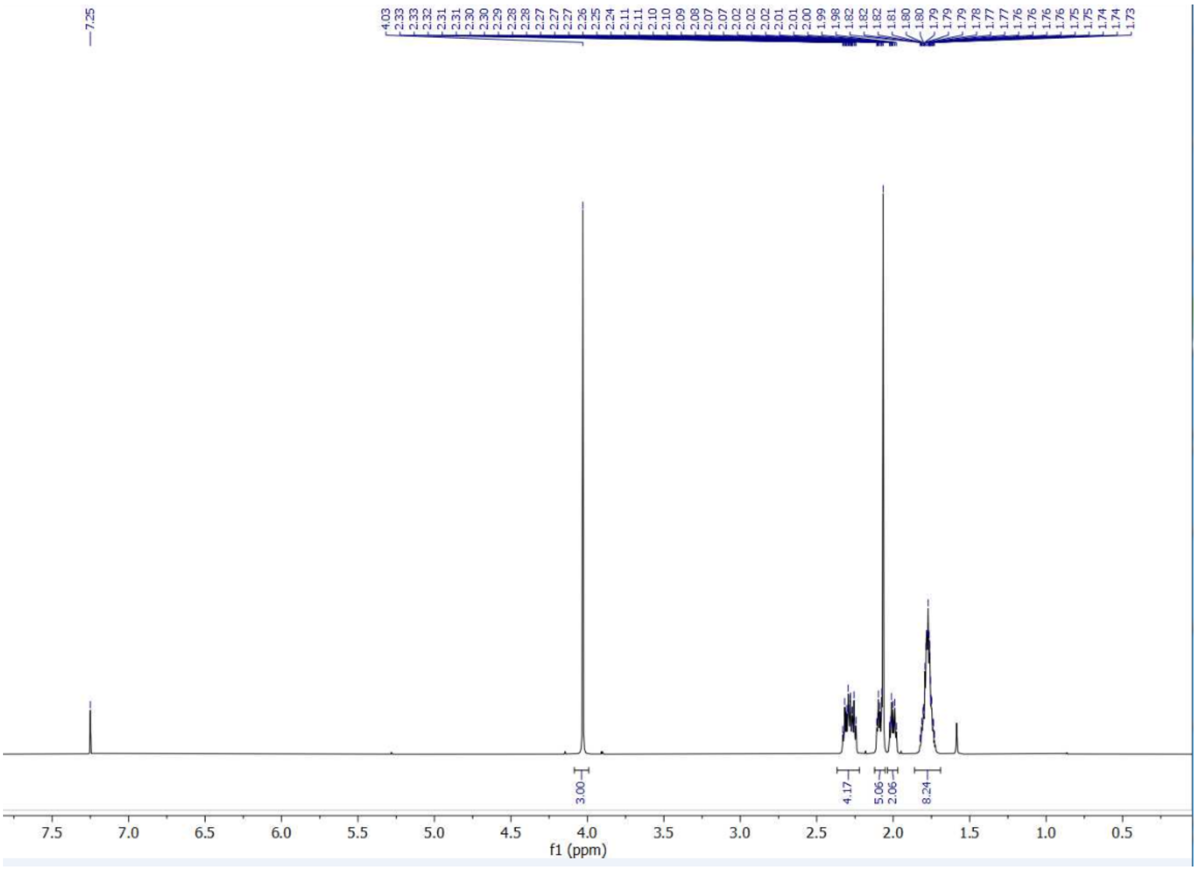

Fig. S13a. The ^{1}H NMR spectrum of **10h**.

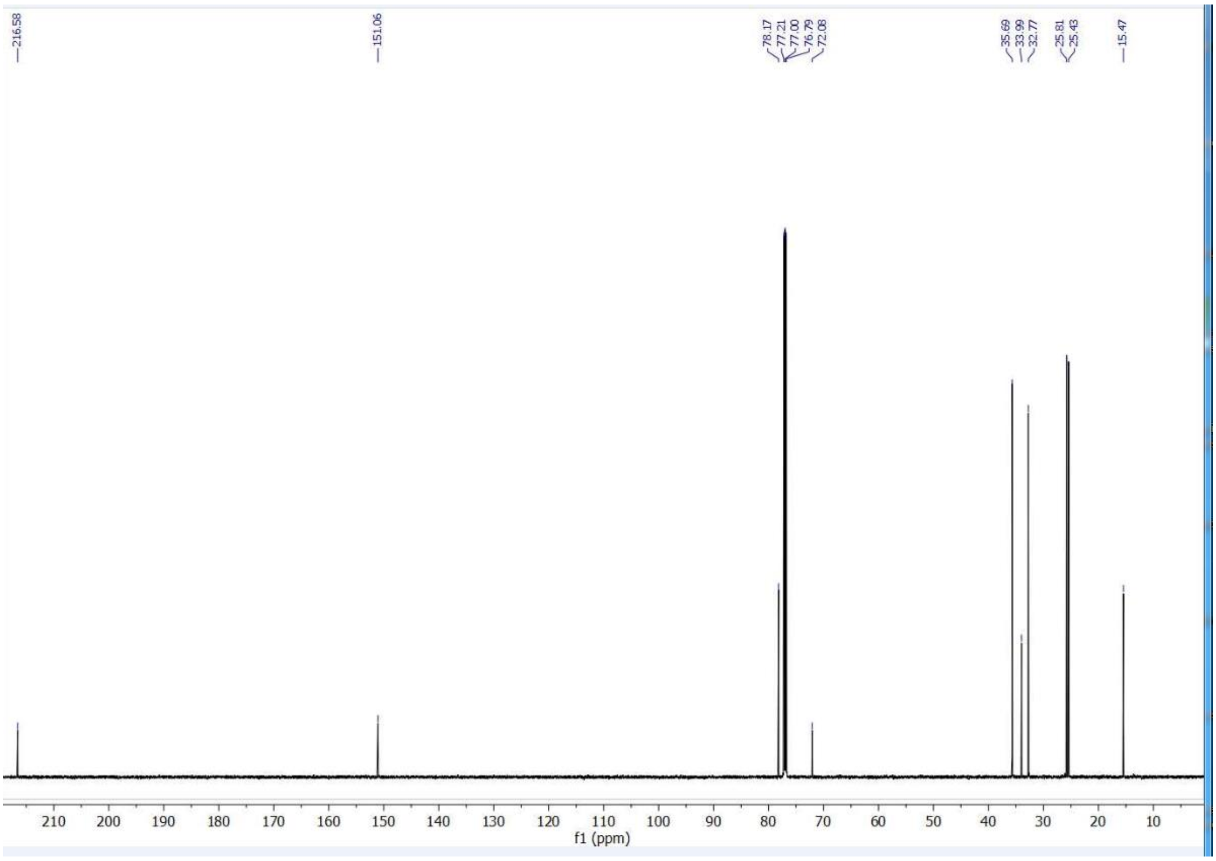

Fig. S13b. The ^{13}C NMR spectrum of **10h**.

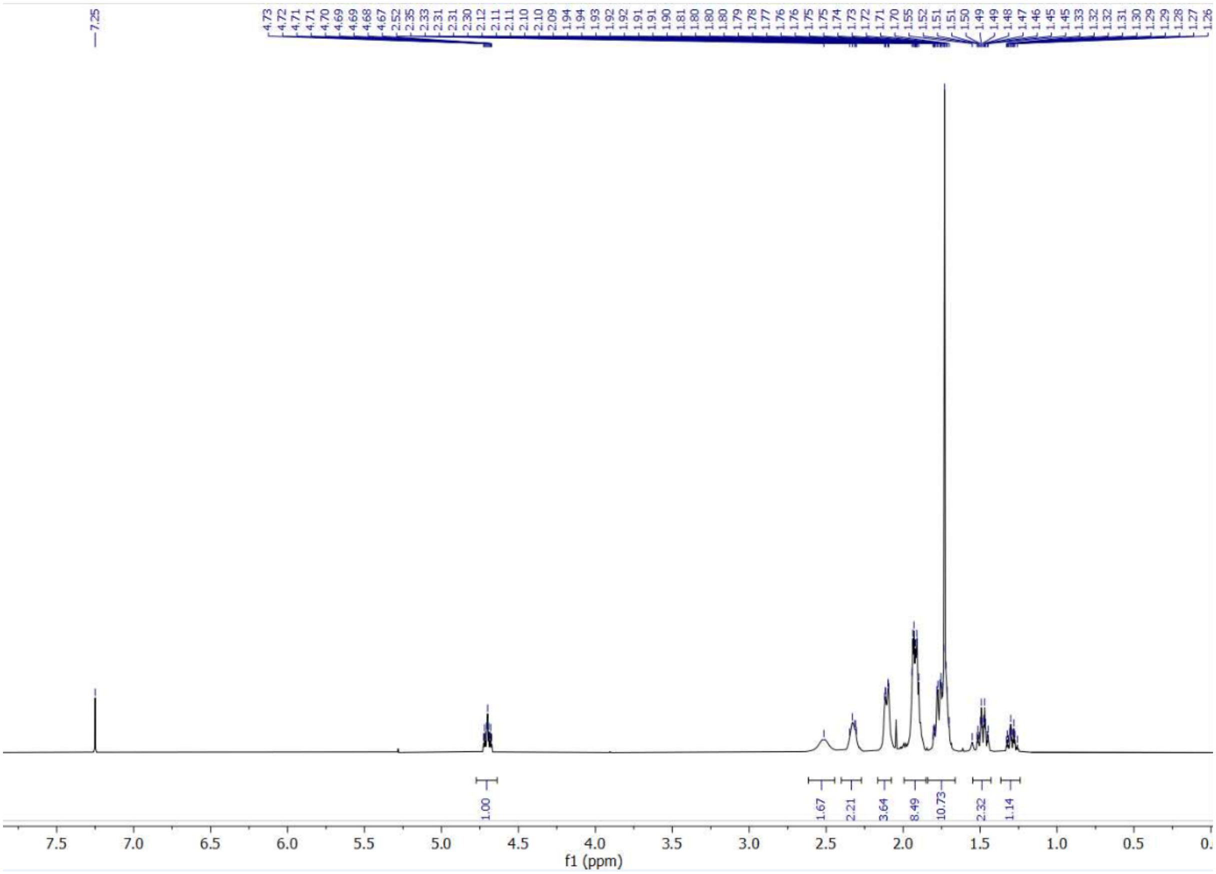

Fig. S14a. The ^{1}H NMR spectrum of **9i**.

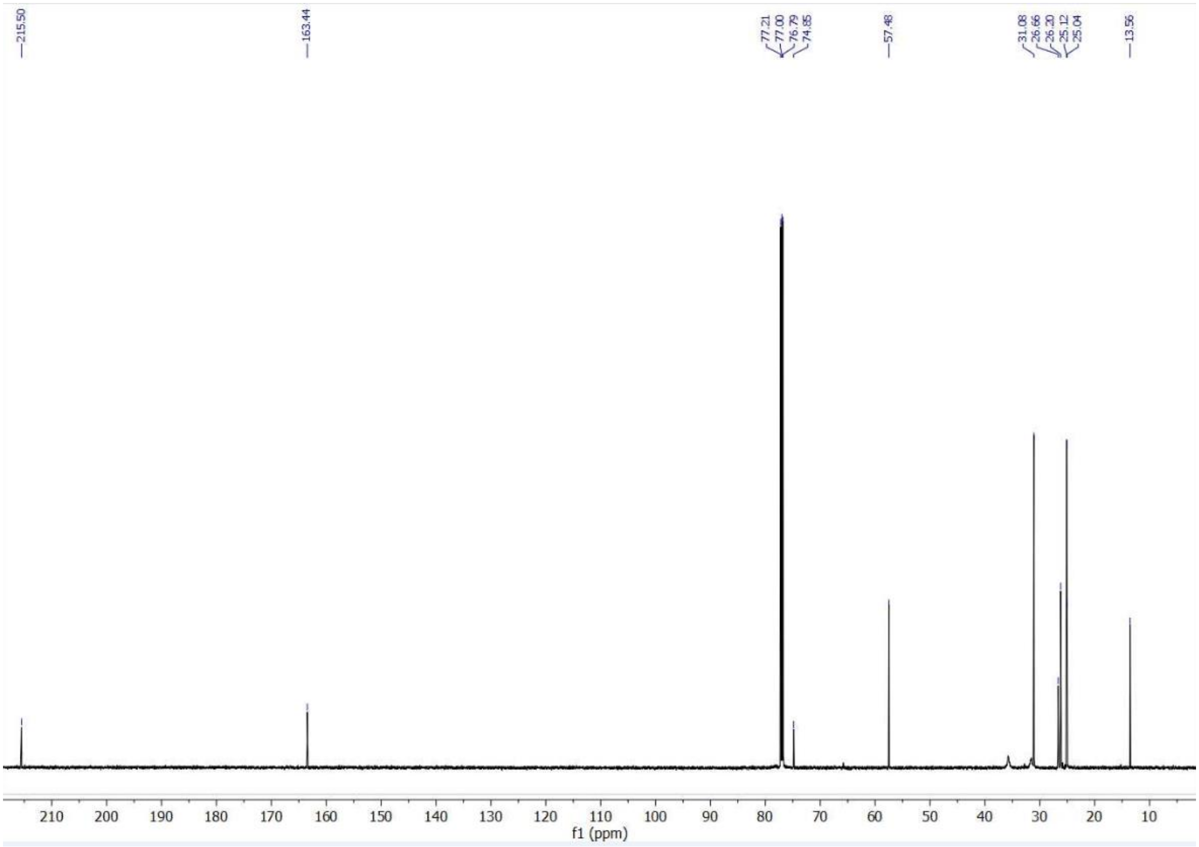

Fig. S14b. The ^{13}C NMR spectrum of **9i**.

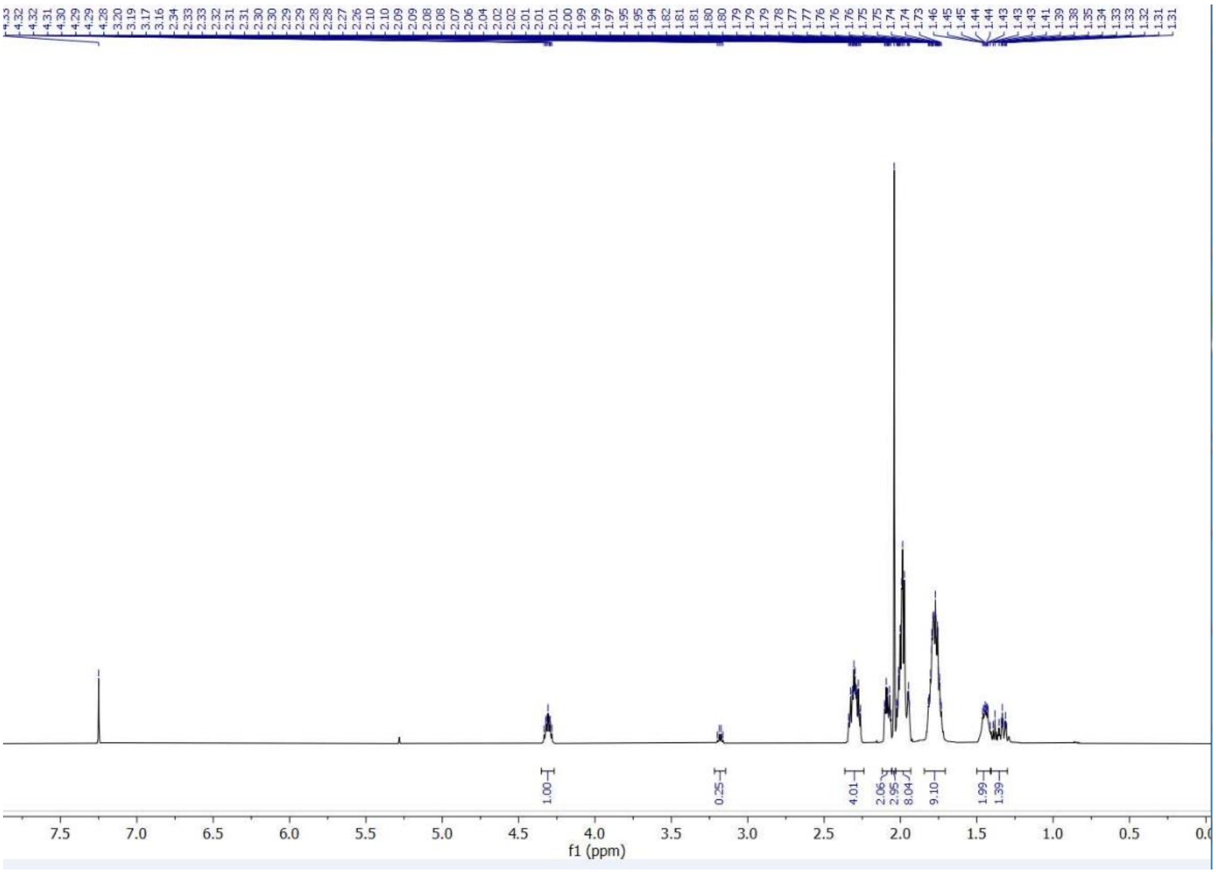

Fig. S15a. The ^{1}H NMR spectrum of **10i**.

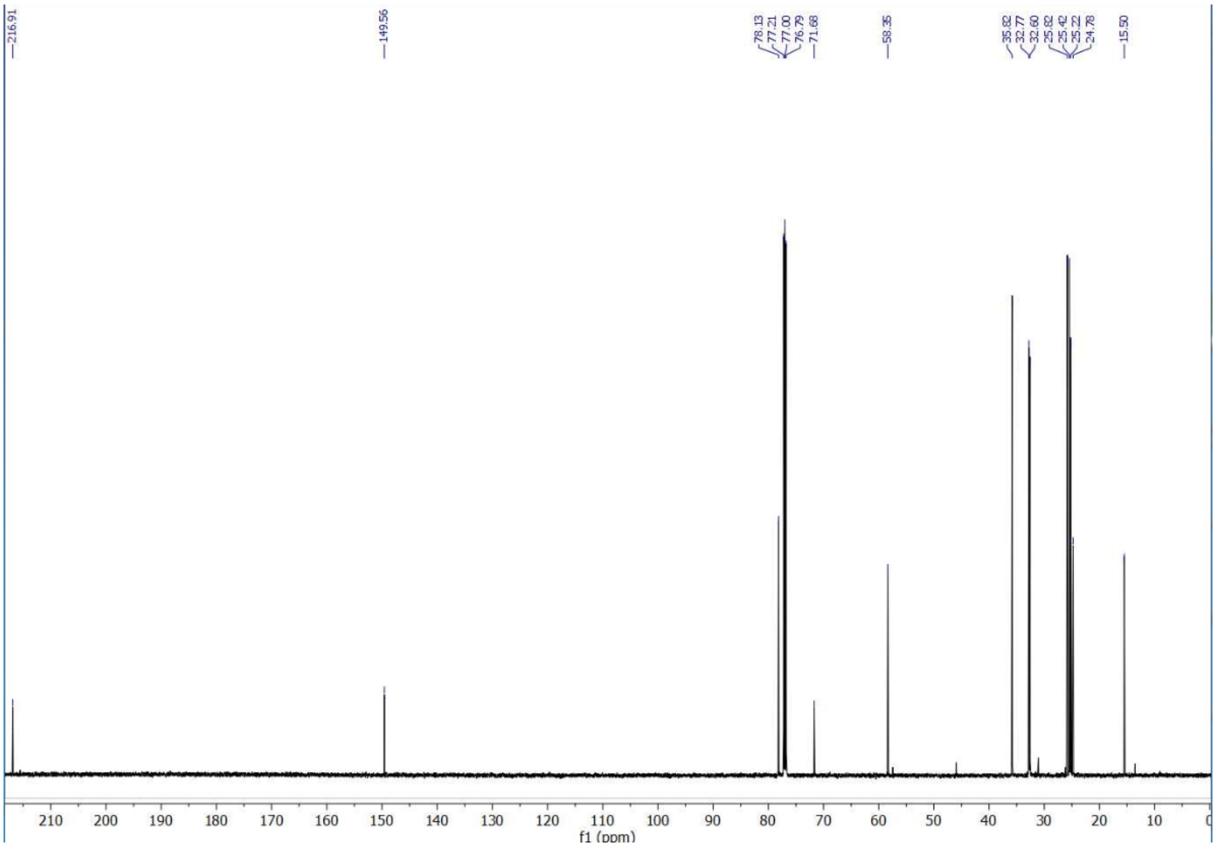

Fig. S15b. The ^{13}C NMR spectrum of **10i**.

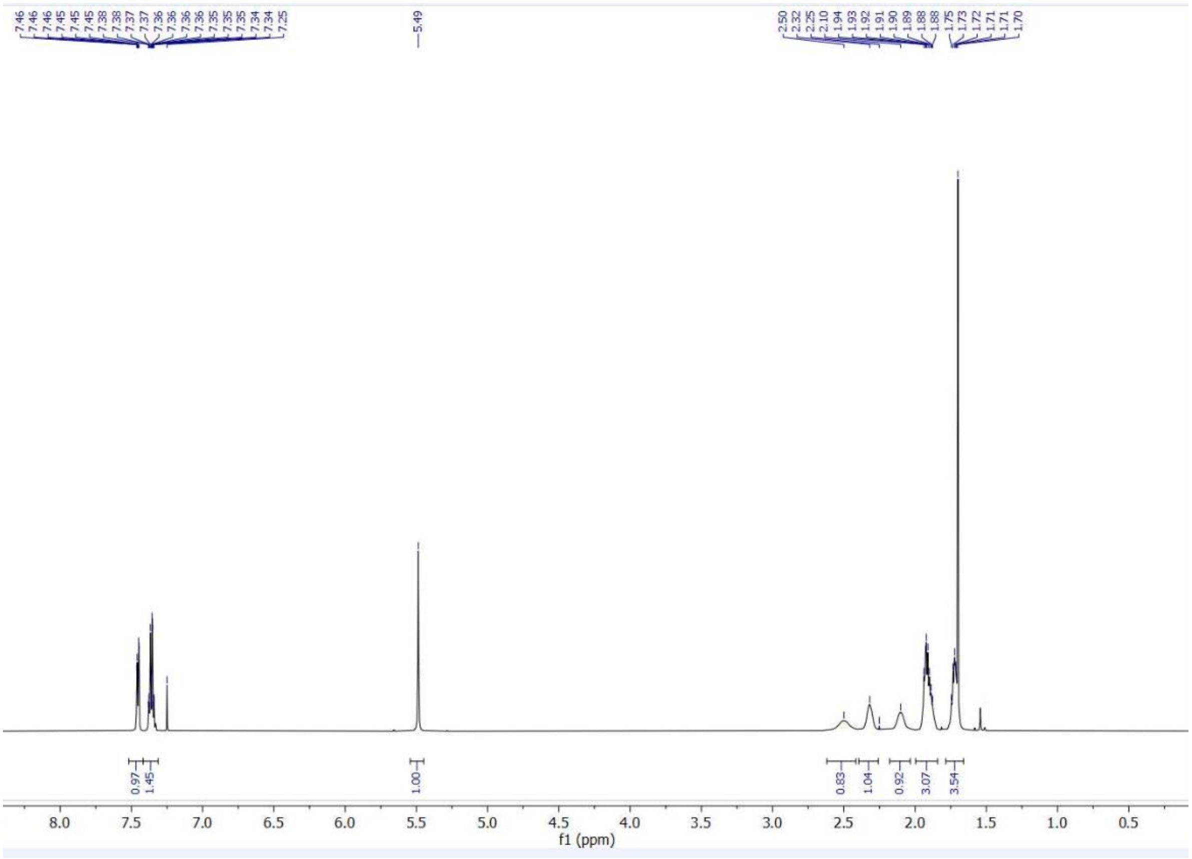

Fig. S16a. The ^{1}H NMR spectrum of **9j**.

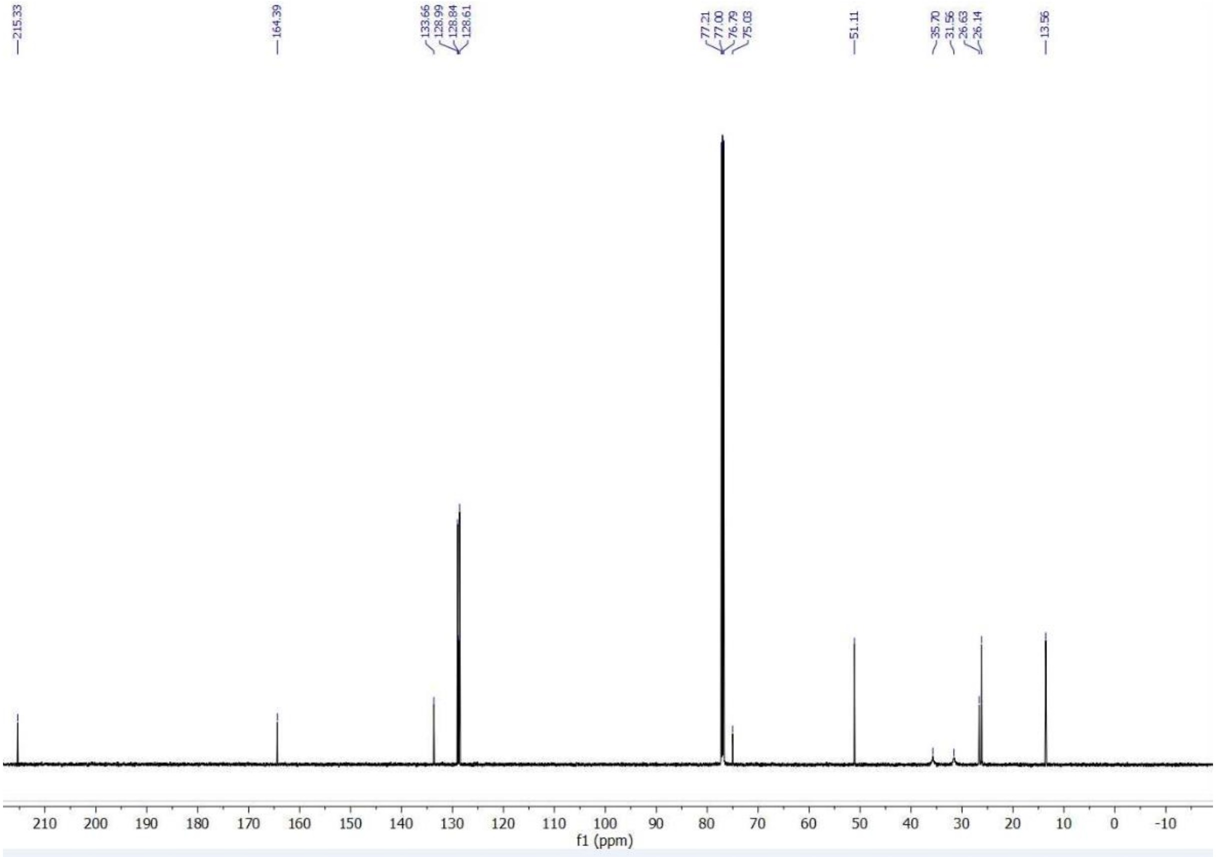

Fig. S16b. The ^{13}C NMR spectrum of **9j**.

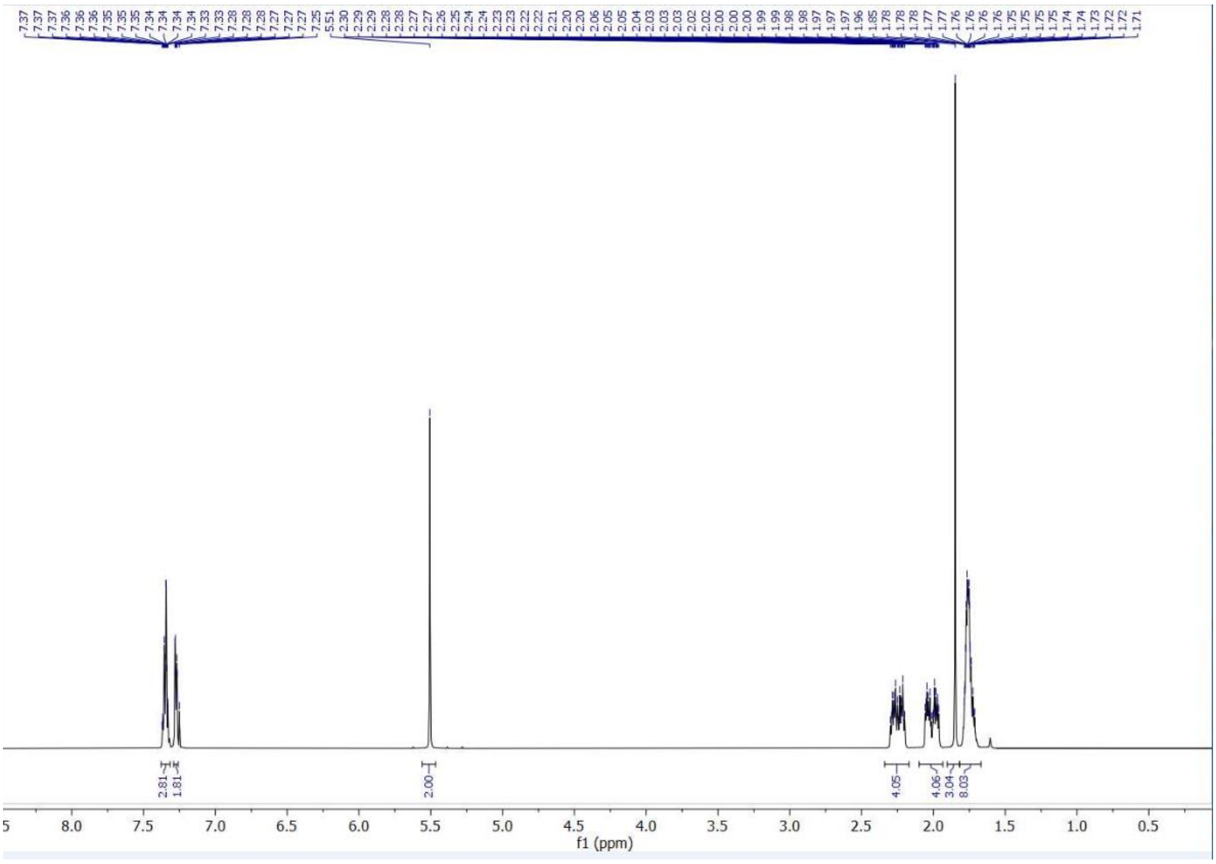

Fig. S17a. The ^{1}H NMR spectrum of **10j**.

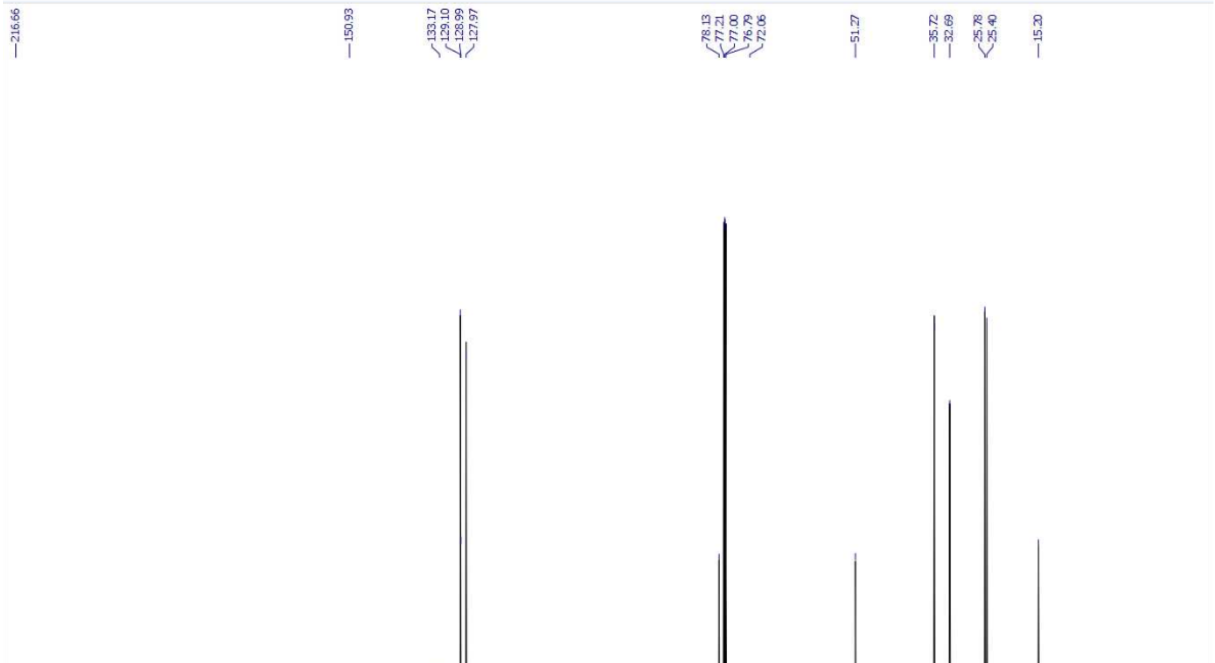

Fig. S17b. The ^{13}C NMR spectrum of **10j**.

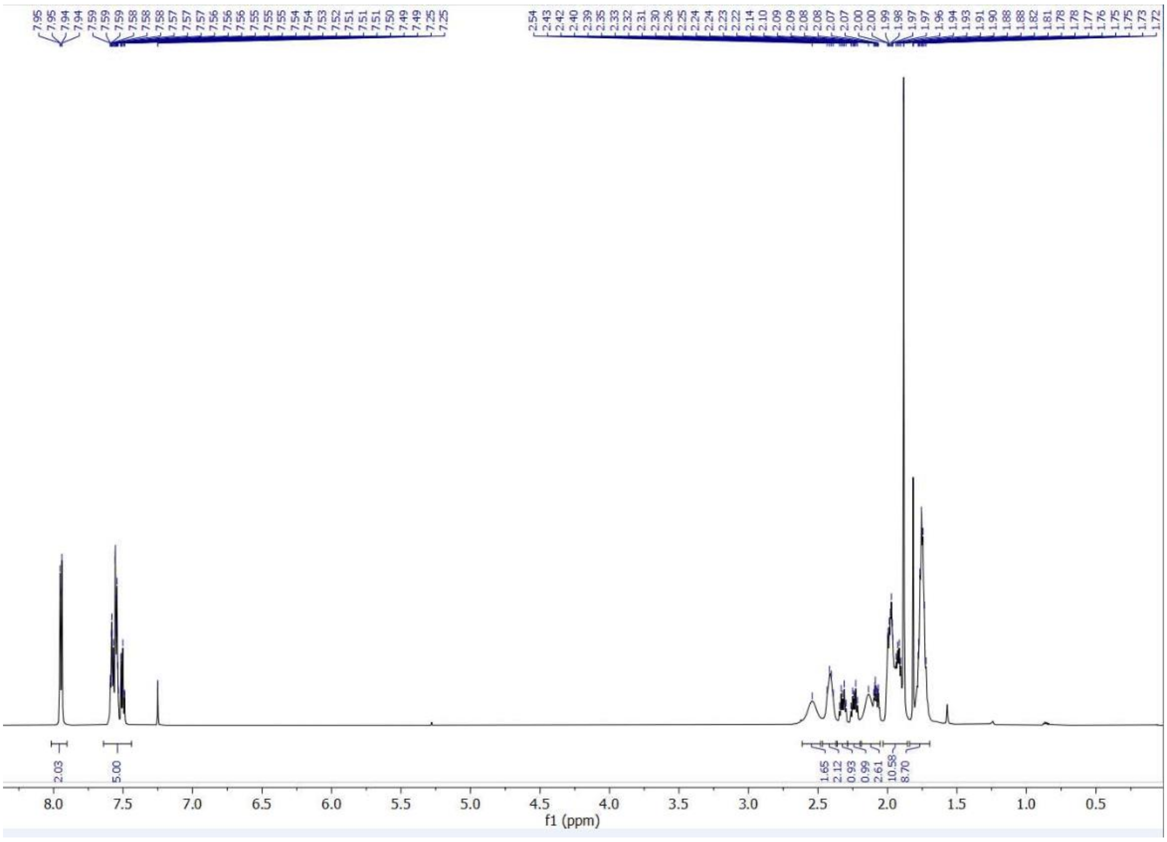

Fig. S18a. The ^{1}H NMR spectrum of **9k** (major) (mixture of isomers **9k** and **10k**).

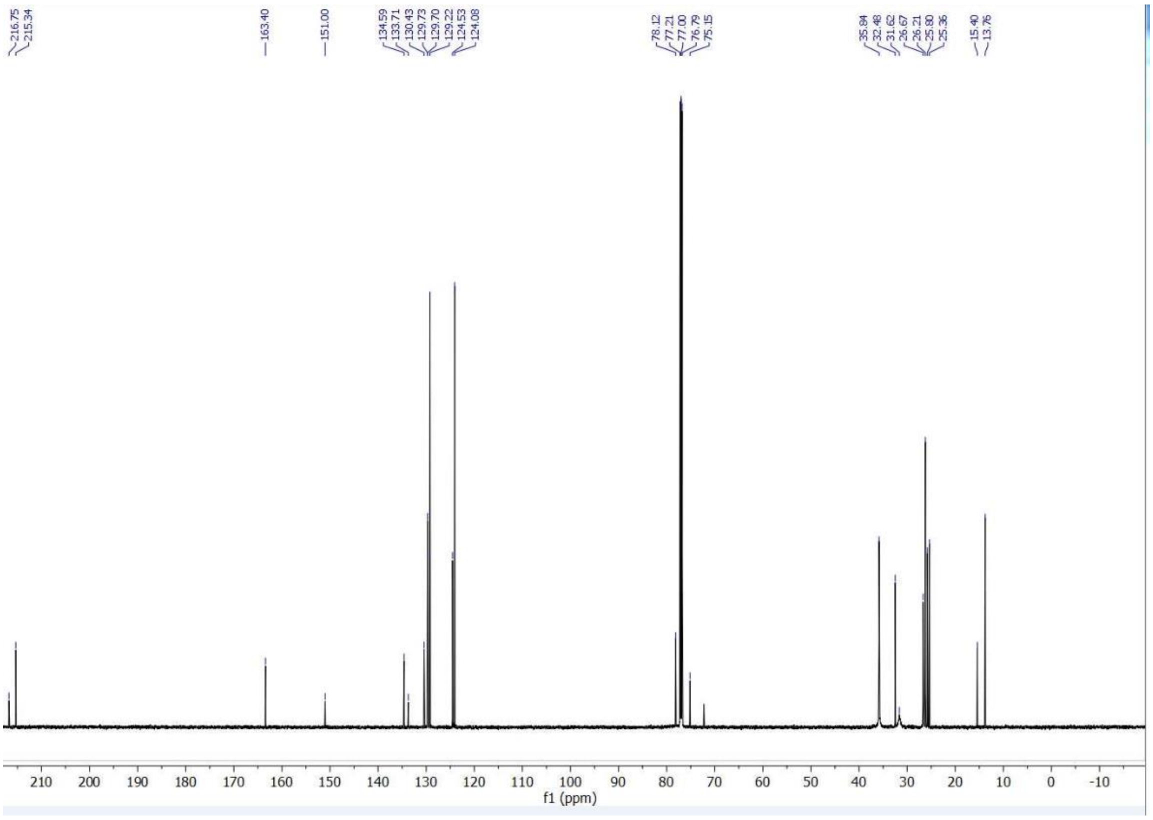

Fig. S18b. The ^{13}C NMR spectrum of **9k** (mixture of isomers **9k** and **10k**).

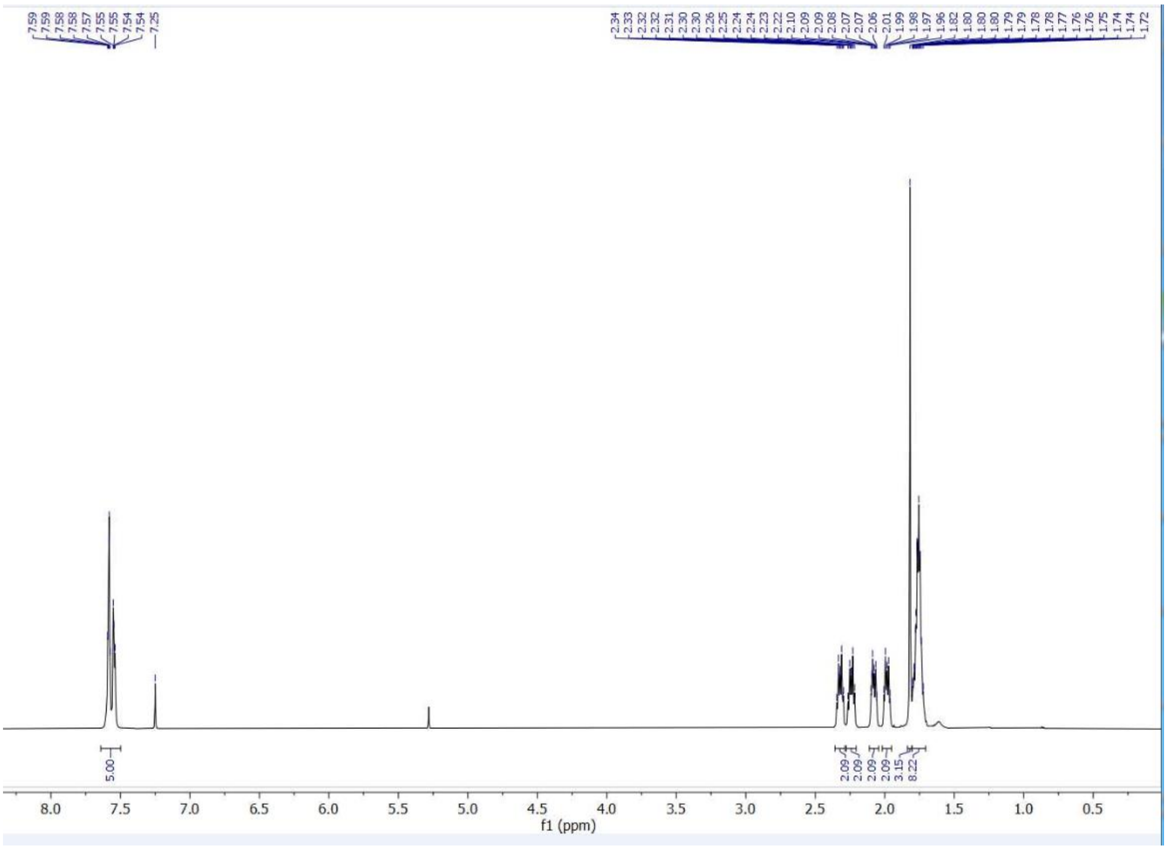

Fig. S19a. The ^{1}H NMR spectrum of **10k**.

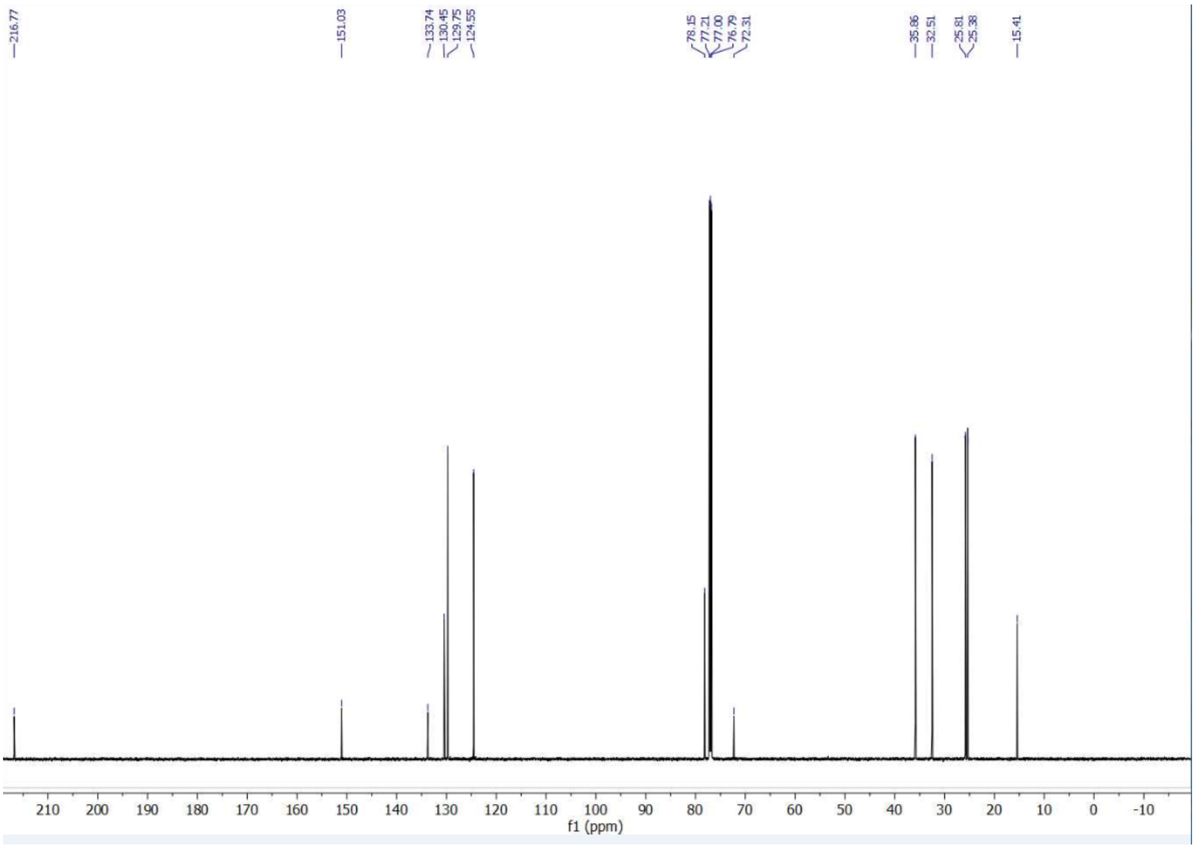

Fig. S19b. The ^{13}C NMR spectrum of **10k**.

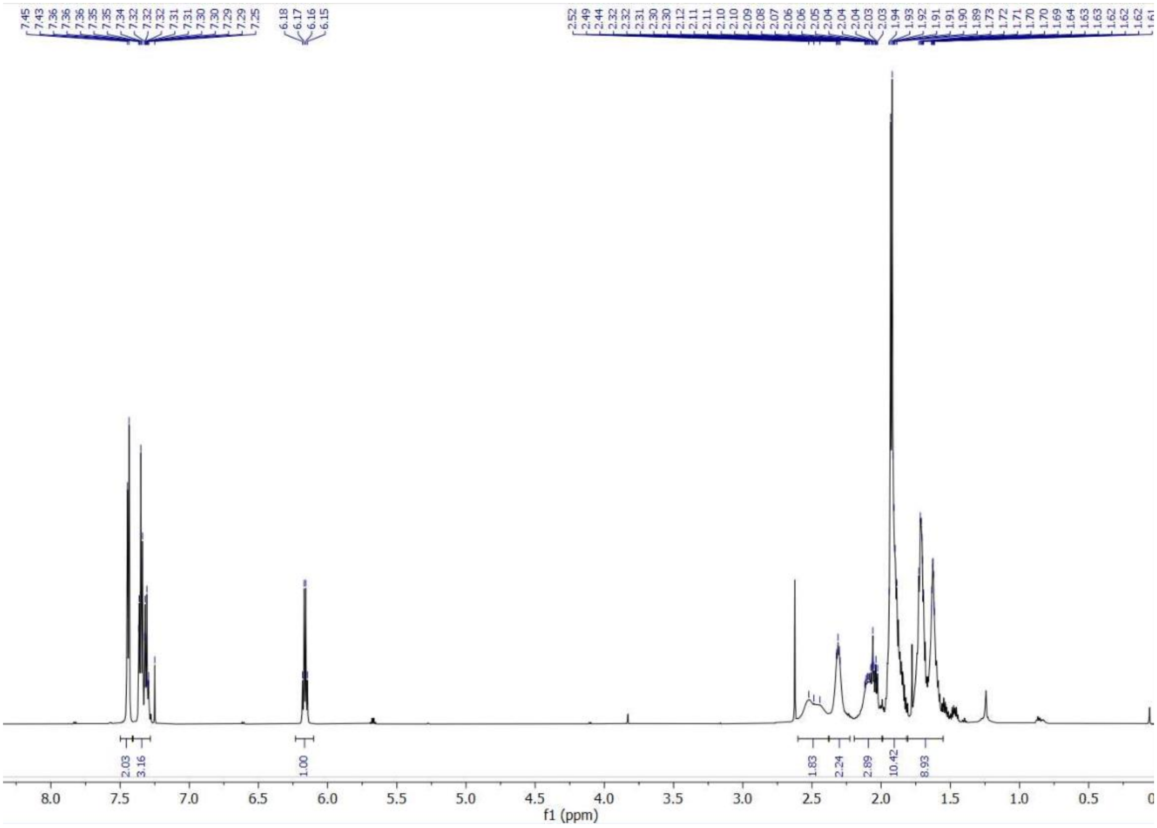

Fig. S20a. The ^{1}H NMR spectrum of **9l**.

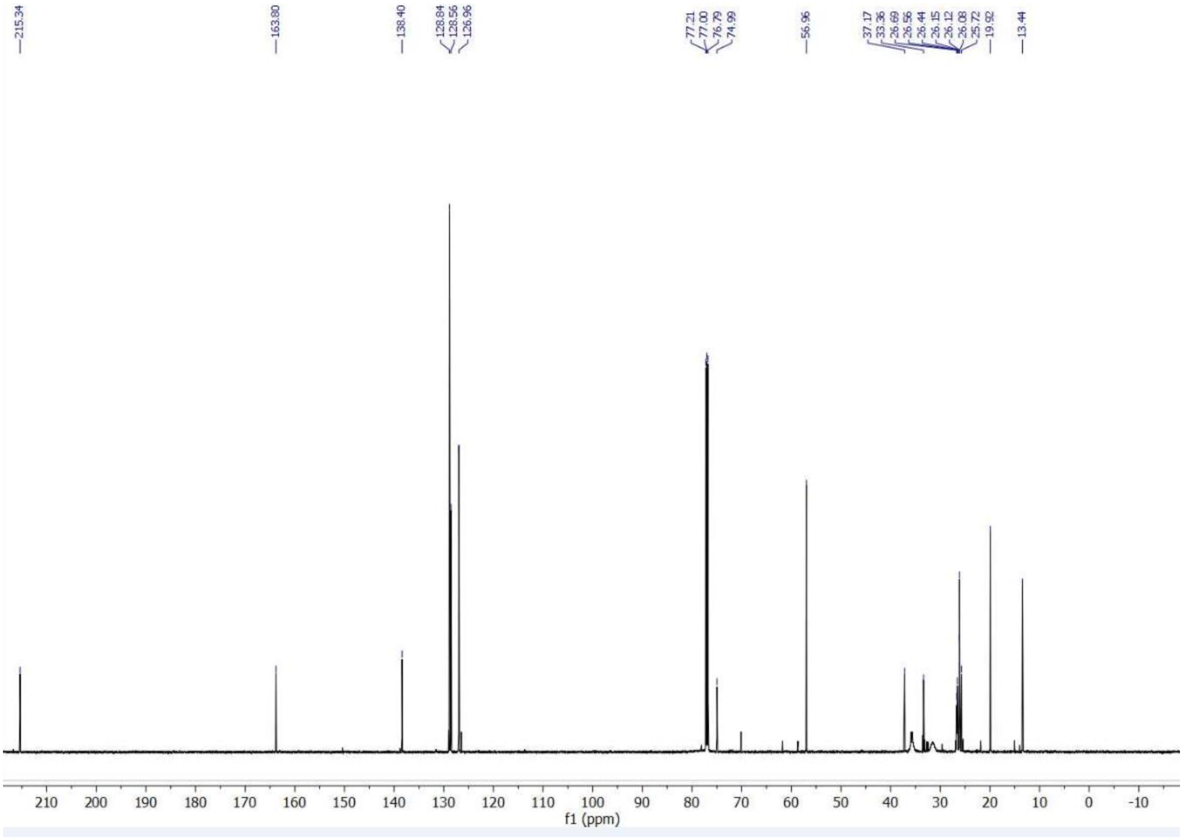

Fig. S20b. The ^{13}C NMR spectrum of **9l**.

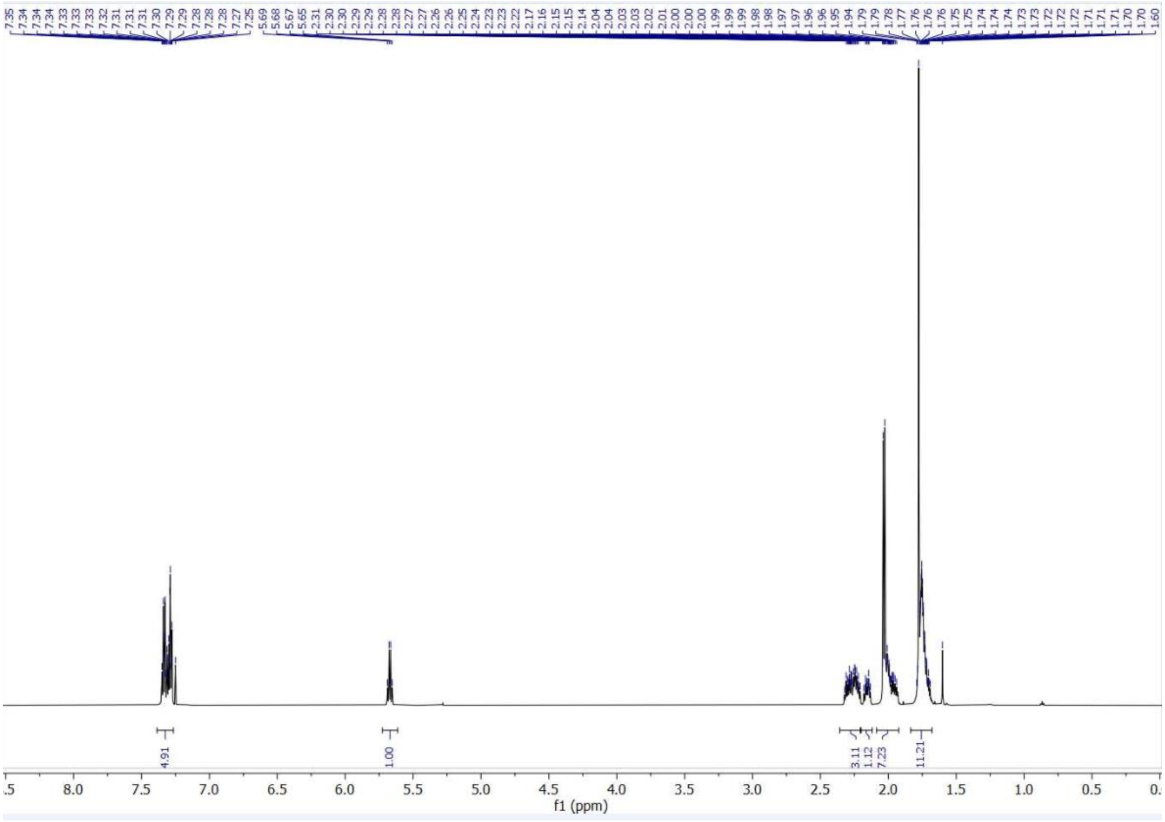

Fig. S21a. The ^{1}H NMR spectrum of **10l**.

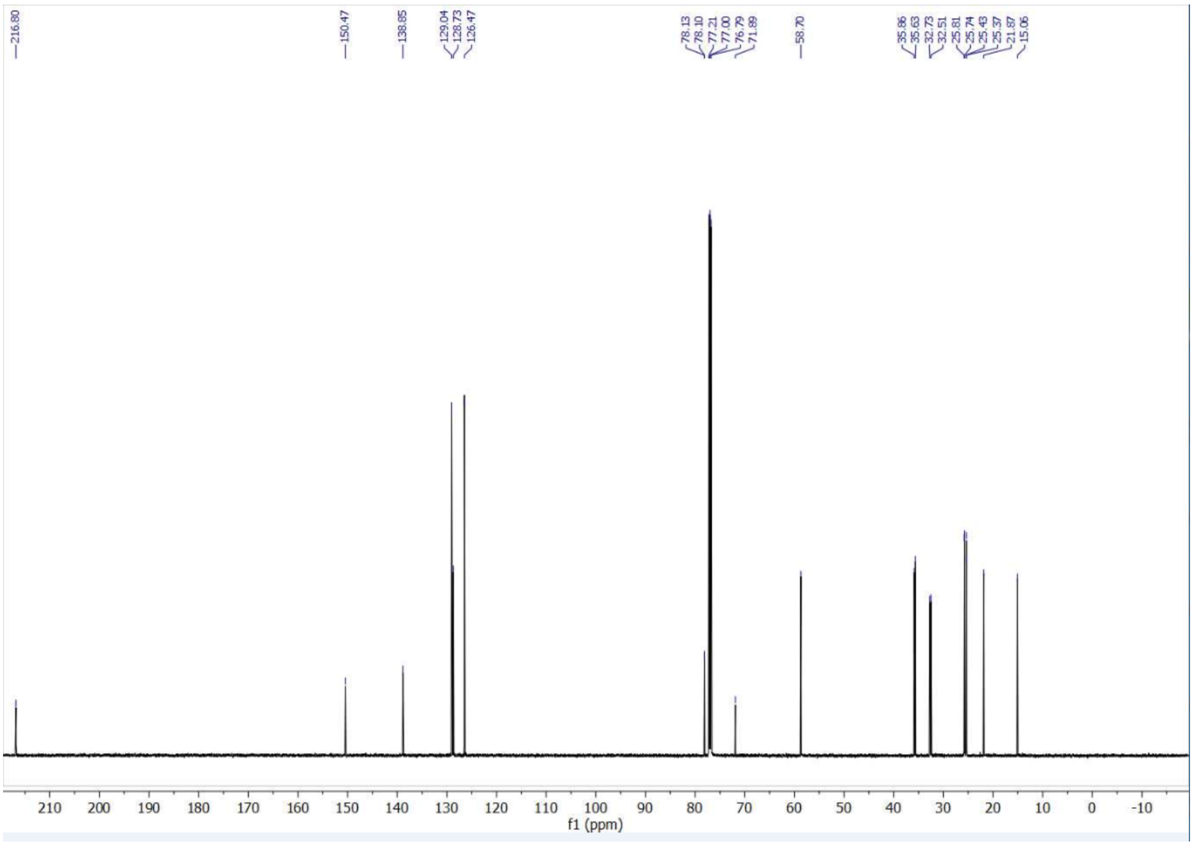

Fig. S21b. The ^{13}C NMR spectrum of **10l**.

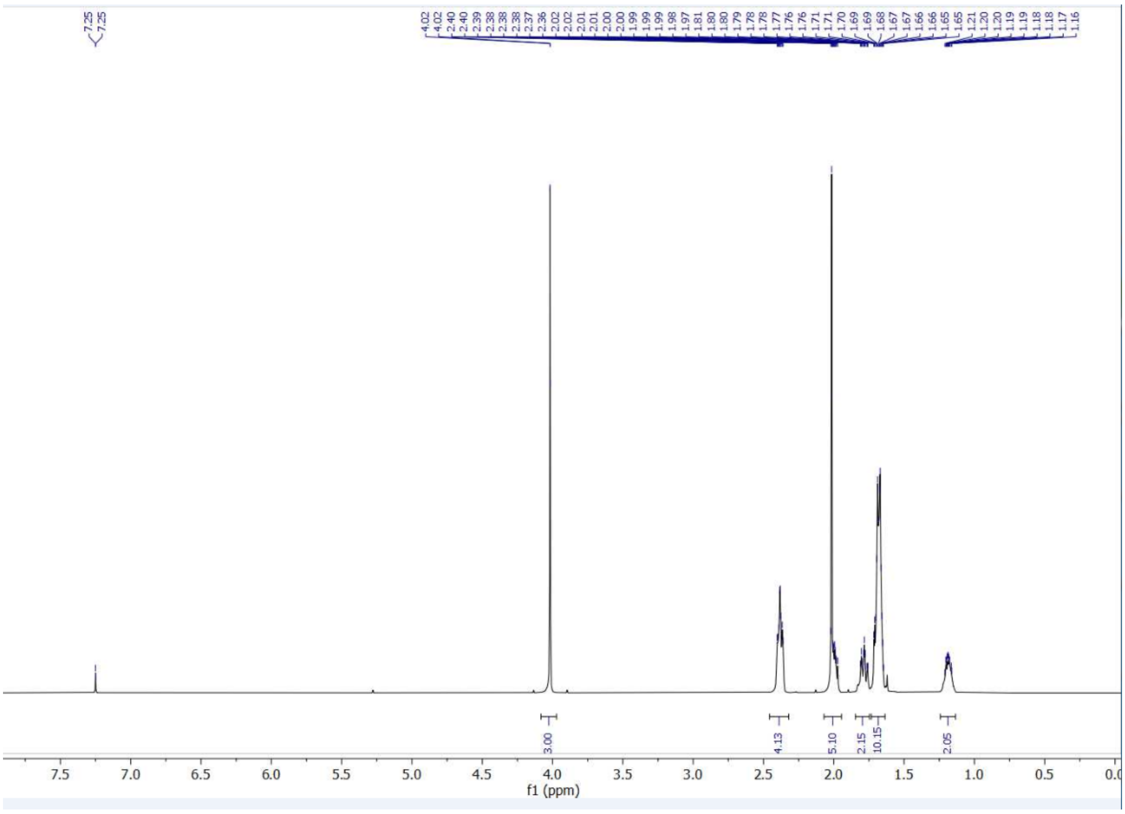

Fig. S22a. The ^{1}H NMR spectrum of **10m**.

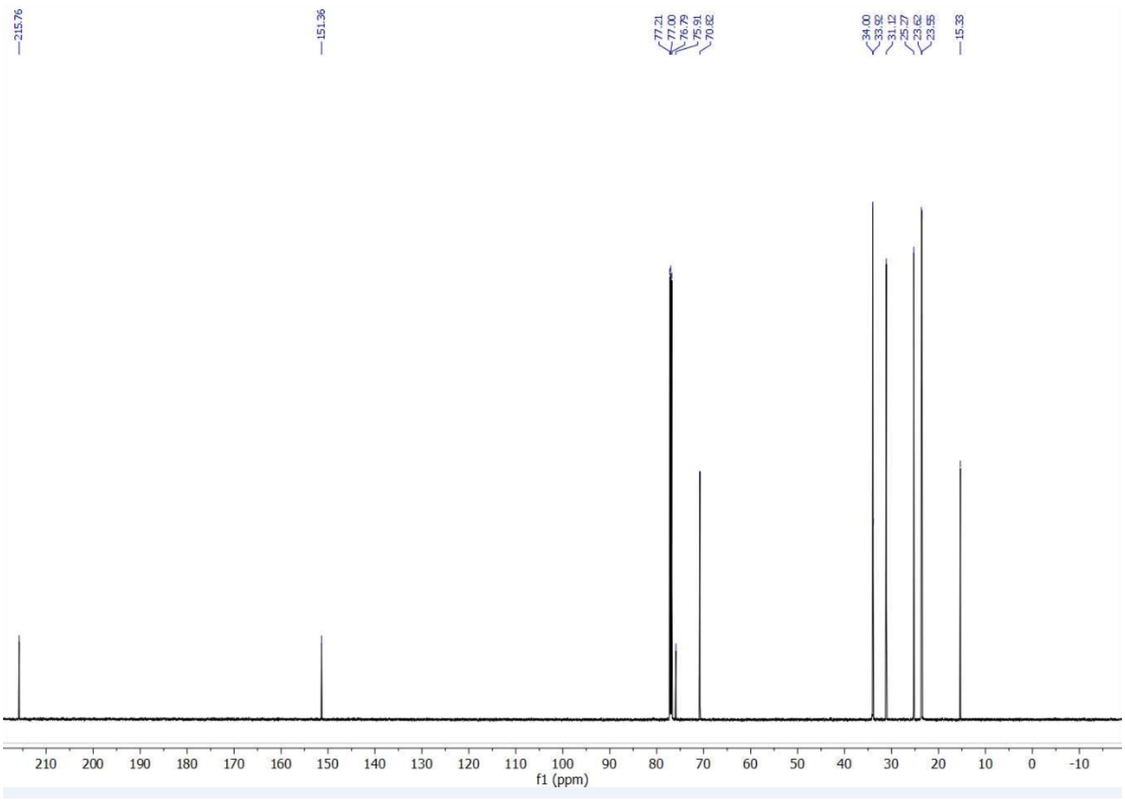

Fig. S22b. The ^{13}C NMR spectrum of **10m**.

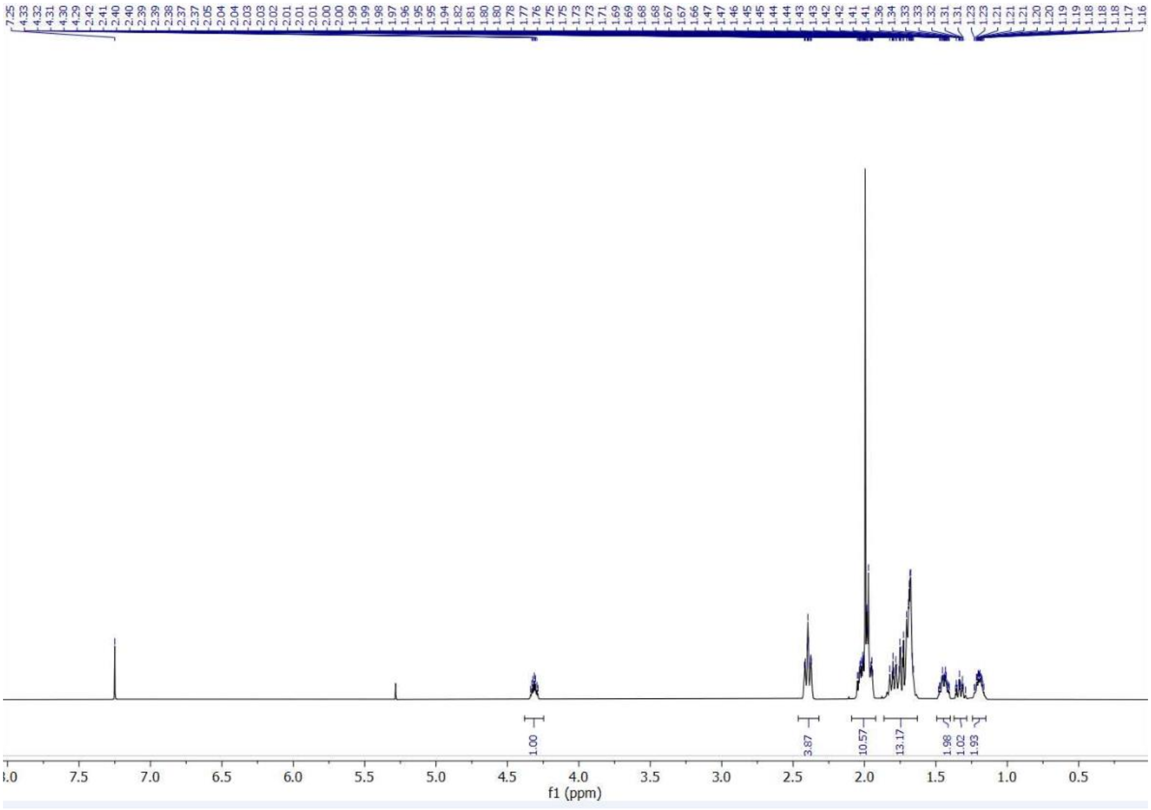

Fig. S23a. The ^{1}H NMR spectrum of **10n**.

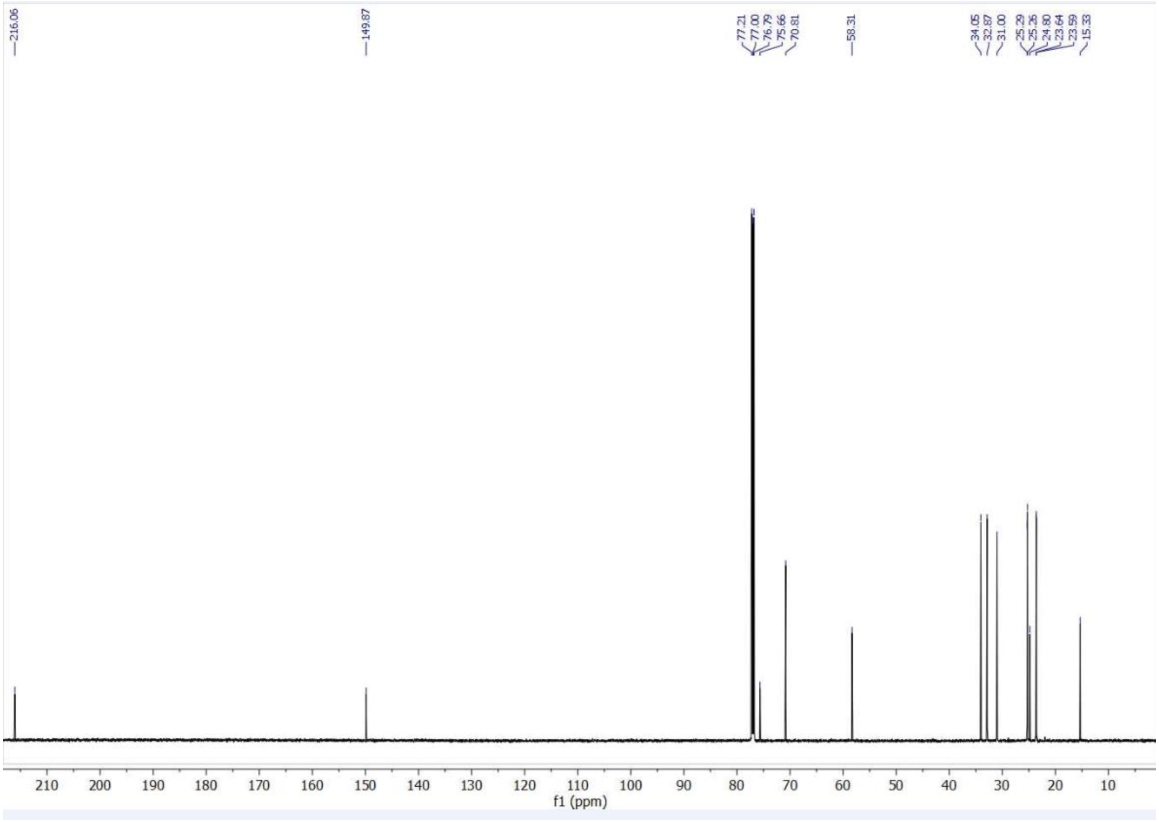

Fig. S23b. The ^{13}C NMR spectrum of **10n**.

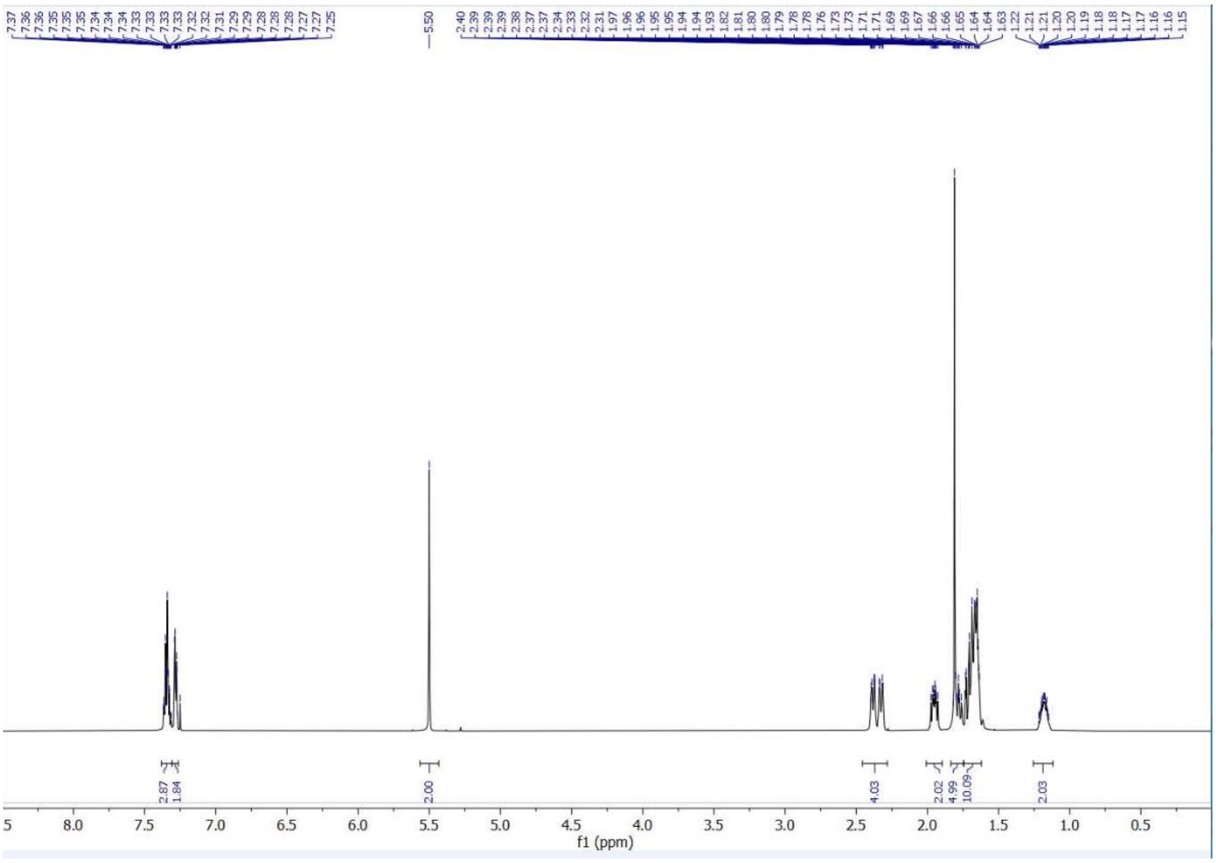

Fig. S24a. The ^{1}H NMR spectrum of **10o**.

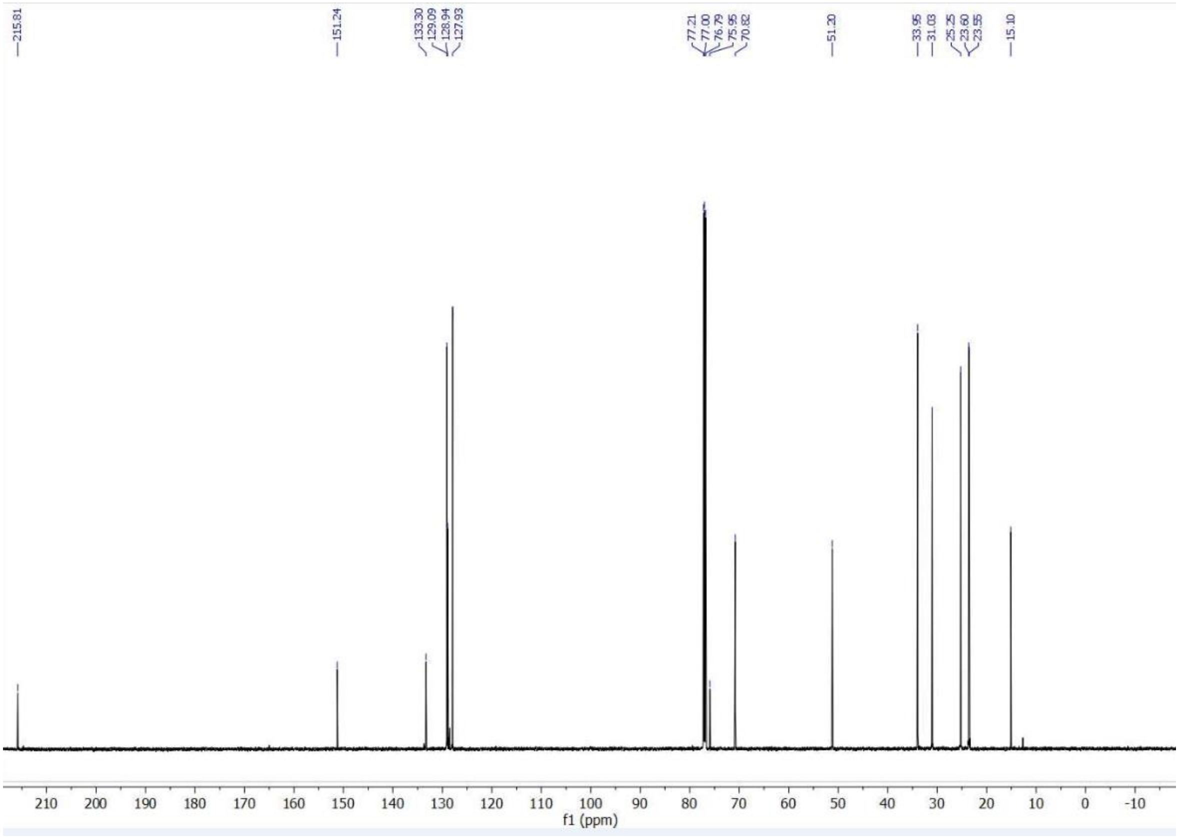

Fig. S24b. The ^{13}C NMR spectrum of **10o**.

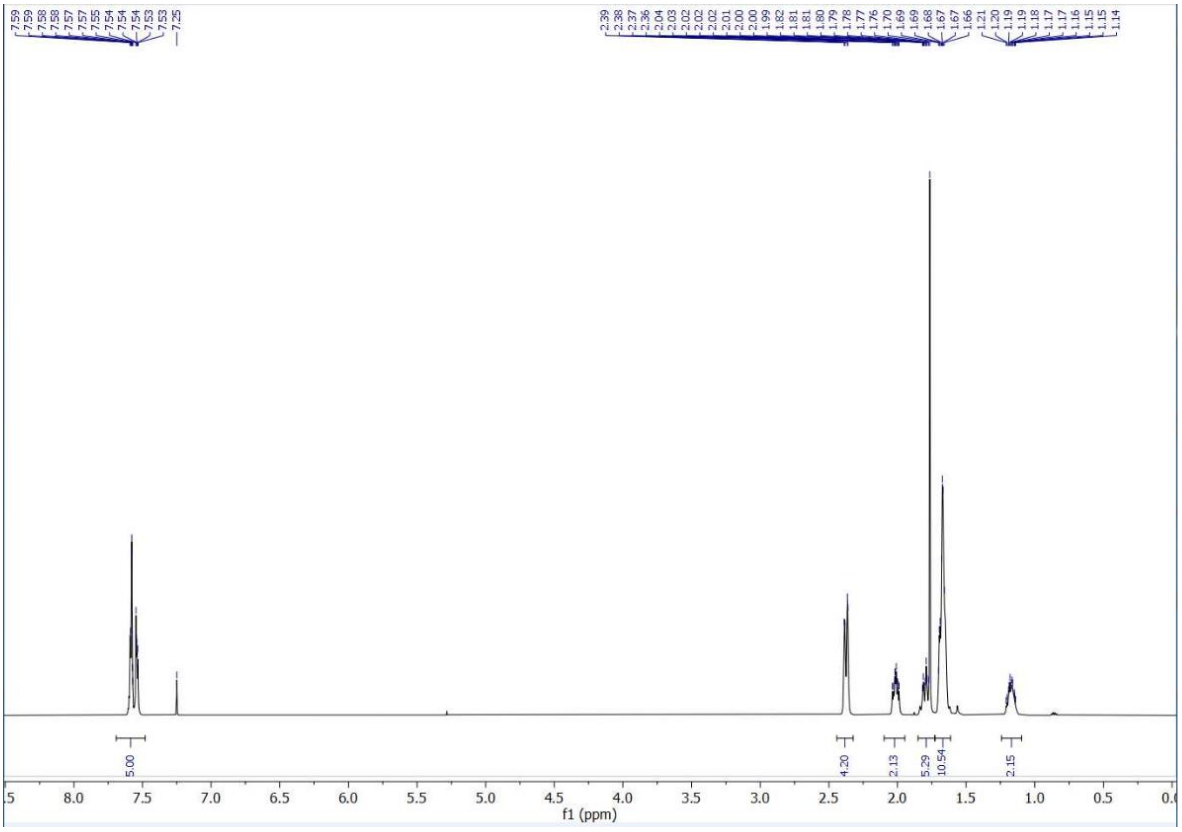

Fig. S25a. The ^{1}H NMR spectrum of **10p**.

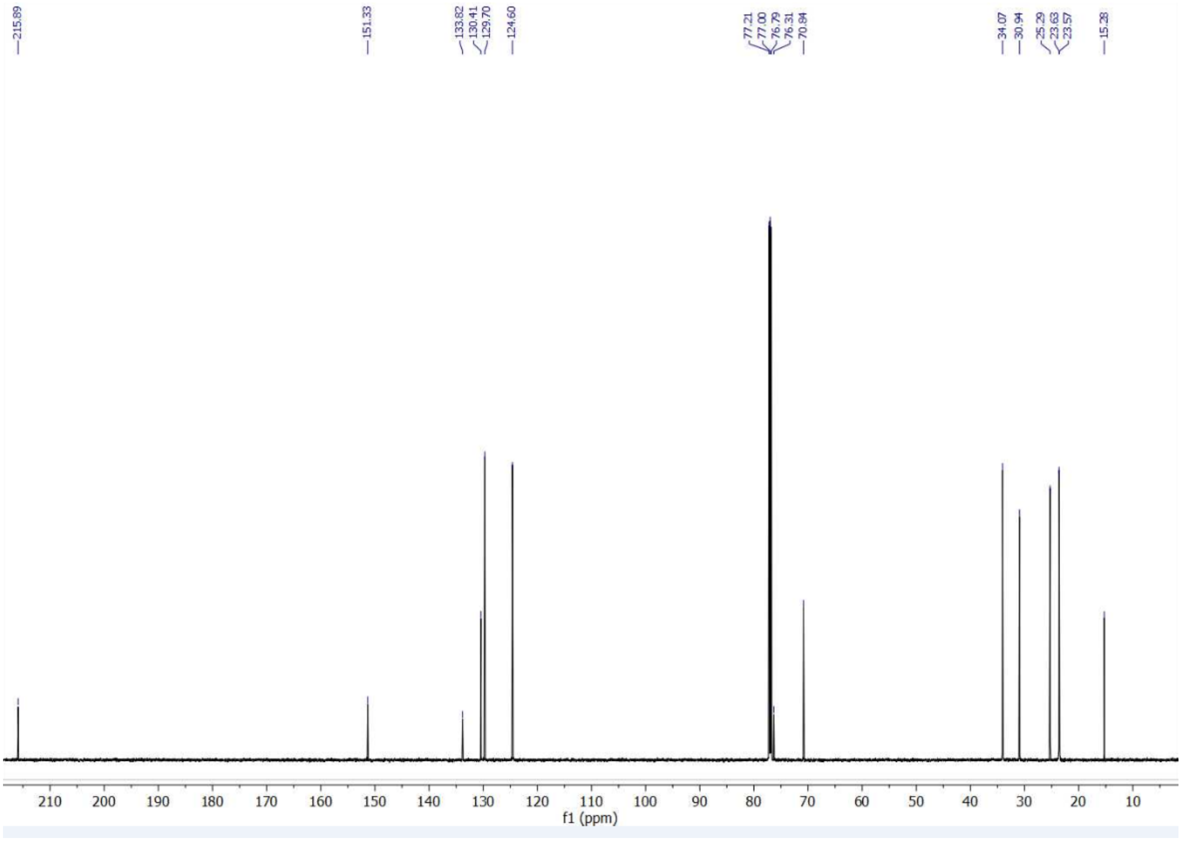

Fig. S25b. The ^{13}C NMR spectrum of **10p**.

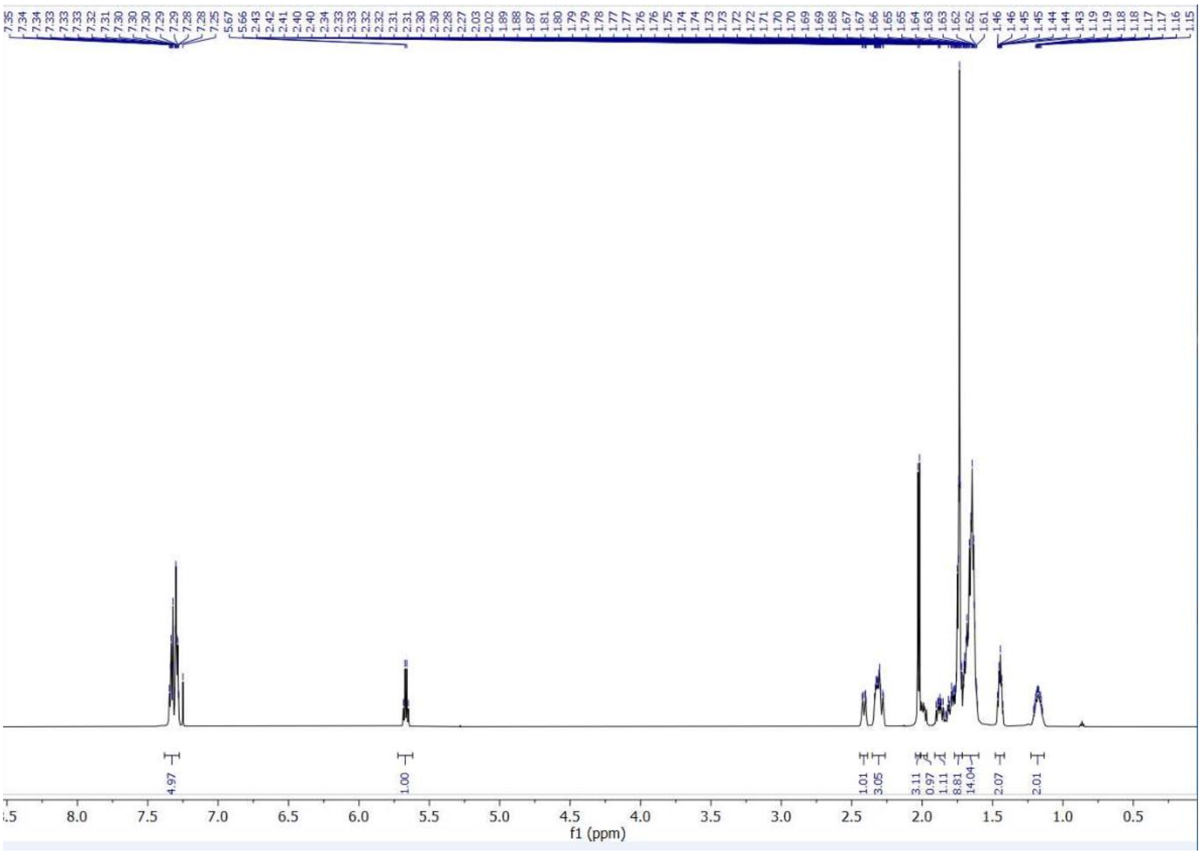

Fig. S26a. The ^{1}H NMR spectrum of **10q**.

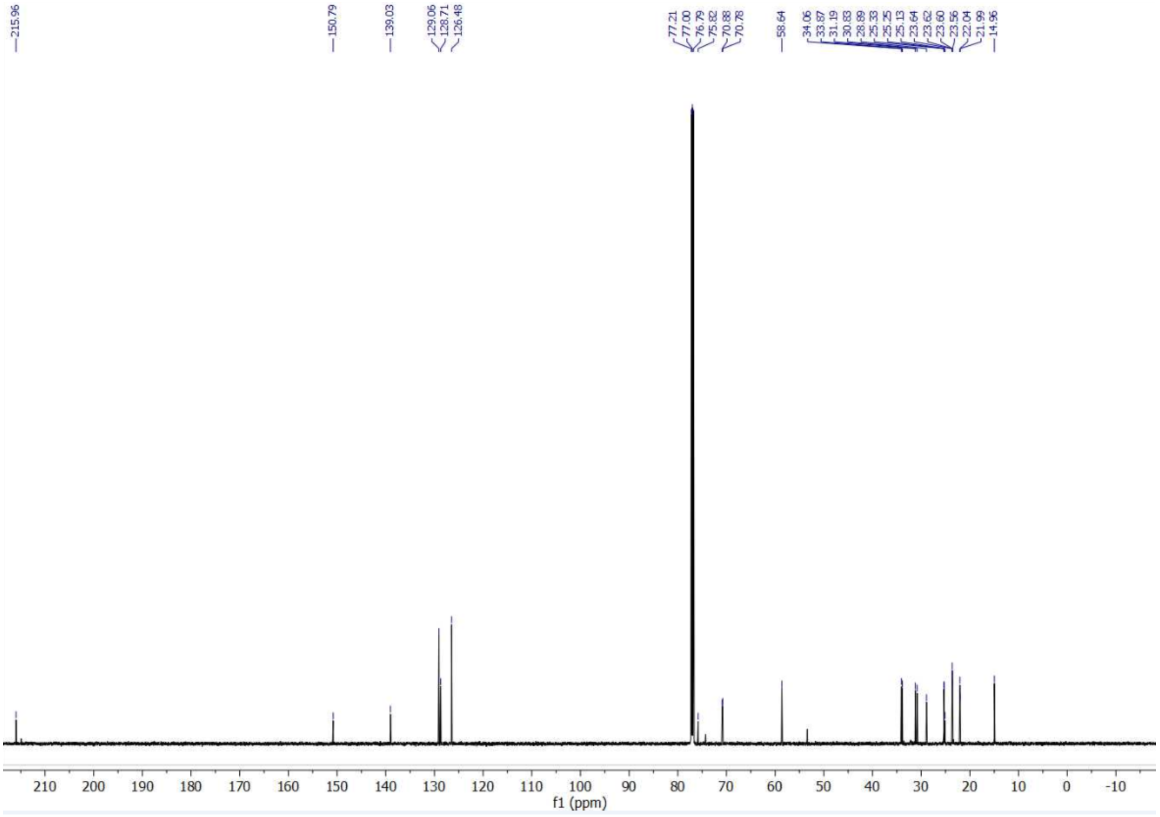

Fig. S26b. The ^{13}C NMR spectrum of **10q**.

Section 4: *X-Ray structure determination of compounds **9i** and **10i***

Description of X-ray data collection experiments: X-ray diffraction data for **9i** and **10i** was collected on an XtaLAB Synergy, Dualflex, HyPix diffractometer. Integration of the intensities and corrections for Lorentz effects, polarization effects, and analytical absorption were performed with CrysAlis PRO [2]. Using Olex2 [3], the structure was solved with the SHELXT [4] structure solution program using Intrinsic Phasing and refined with the SHELXL [5] refinement package using Least Squares minimization. The hydrogen atoms were introduced in the calculated positions with an idealized geometry and constrained using a rigid body model with isotropic displacement parameters equal to 1.2 of the equivalent displacement parameters of their parent atoms. The molecular geometries were calculated by the PLATON program [6]. The relevant crystallographic data are given in Table S1 (SI). Atomic coordinates, displacement parameters, and structural factors of the analyzed crystal structures are deposited with the Cambridge Crystallographic Data Centre CCDC (reference number: 2420216 and 2420217 for **9i** and **10i**, respectively) [7].

Table S1. Crystal data and structure refinement for **9i** and **10i**.

Identification code	**9i**	**10i**
Empirical formula	$C_{20}H_{30}N_4OS_2$	$C_{80}H_{123}N_{16}O_4S_8$
Formula weight	406.60	1629.502
Temperature/K	294.78(10)	295.5(2)
Crystal system	monoclinic	monoclinic
Space group	$P2_1/c$	Cc
a/Å	9.90450(10)	23.3932(3)
b/Å	19.38640(10)	13.3079(1)
c/Å	11.64400(10)	28.0596(3)
α/°	90	90
β/°	108.2260(10)	100.966(1)
γ/°	90	90
Volume/Å^3	2123.62(3)	8575.85(16)
Z	4	4
ρ_{calc}mg/mm^3	1.272	1.262
μ/mm^{-1}	2.402	2.380

F(000)	872.0	3520.1
Crystal size/mm^3	0.295 × 0.165 × 0.104	0.339 × 0.254 × 0.092
Radiation	Cu Kα (λ = 1.54184)	Cu Kα (λ = 1.54184)
2Θ range for data collection	9.124 to 152.982	6.42 to 137
Index ranges	-11 ≤ h ≤ 12, -23 ≤ k ≤ 24, -13 ≤ l ≤ 14	-28 ≤ h ≤ 29, -15 ≤ k ≤ 16, -34 ≤ l ≤ 34
Reflections collected	40027	64064
Independent reflections	4371 [R_{int} = 0.0309, R_{sigma} = 0.0134]	14607 [R_{int} = 0.0980, R_{sigma} = 0.1020]
Data/restraints/parameters	4371/749/370	14607/2/977
Goodness-of-fit on F^2	1.076	1.019
Final R indexes [I>=2σ (I)]	R_1 = 0.0308, wR_2 = 0.0879	R_1 = 0.0777, wR_2 = 0.1990
Final R indexes [all data]	R_1 = 0.0326, wR_2 = 0.0893	R_1 = 0.0907, wR_2 = 0.2151
Largest diff. peak/hole / e Å^{-3}	0.20/-0.21	0.94/-0.58
CCDC numer	2420216	2420217

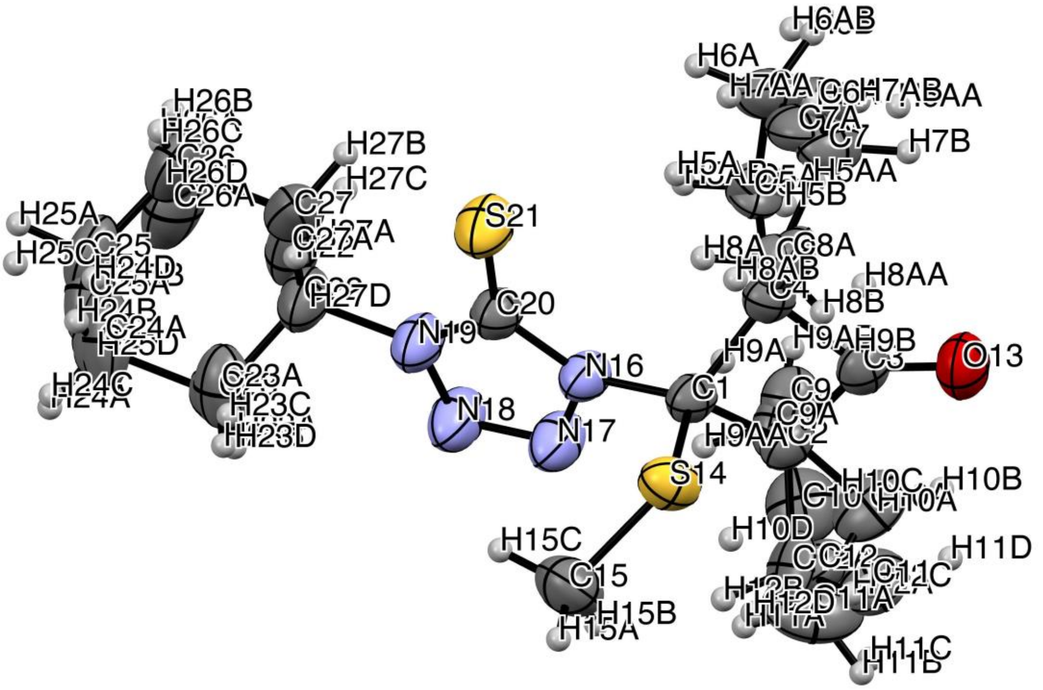

(a)

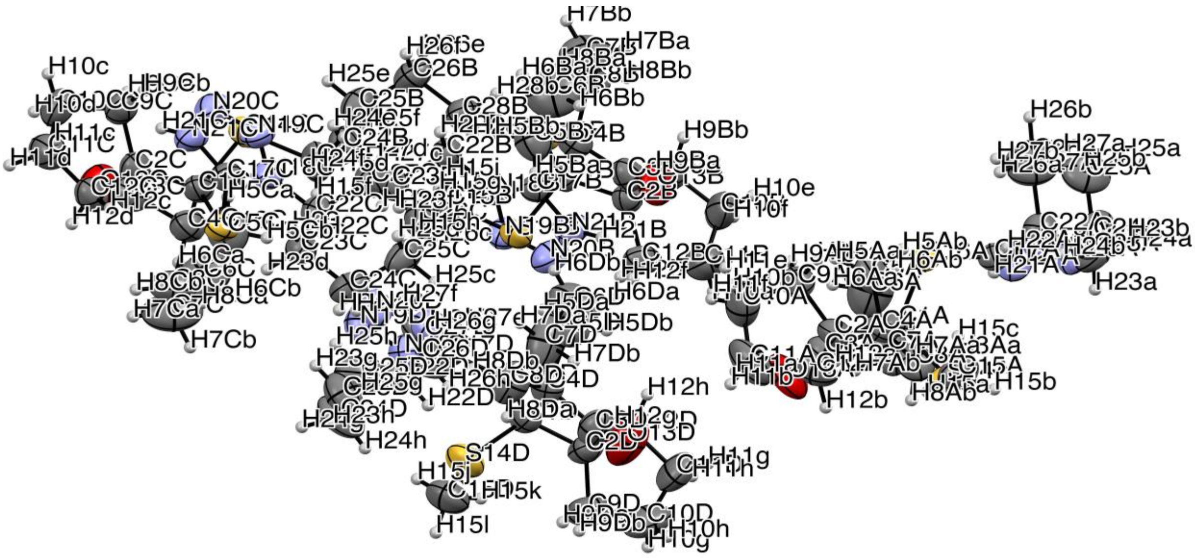

(b)

Figure S27. ORTEP molecular graphs with crystallographic labelling scheme for **9i** (a) and **10i** (b). Ellipsoids drawn with 50% probability. Molecule of **9i** is strongly disordered in the unit cell. In case of **10i** there are four molecules in the asymmetric cell unit.

Section 5: *List of references*

[1] Białek-Pietras, M.; Olejniczak, A. B.; Paradowska, E.; Studzińska, M.; Jabłońska, A.; Leśnikowski, Z. J. Synthesis, susceptibility to enzymatic phosphorylation, cytotoxicity and in vitro antiviral activity of lipophilic pyrimidine nucleoside/carborane conjugates. *J. Organomet. Chem.* **2018**, *865*, 166–172. DOI: org/10.1016/j.jorganchem.2018.03.026]

[2] CrysAlisPRO software system, Oxford Diffraction/Agilent Technologies UK Ltd, Yarnton, England, 2015.

[3] Dolomanov, O.V.; Bourhis, L.J.; Gildea, R.J.; Howard, J.A.K.; Puschmann, H. OLEX2: A Complete Structure Solution, Refinement and Analysis Program. *J. Appl. Crystallogr*. **2009**, *42*, 339–341. DOI: 10.1107/S0021889808042726.

[4] Sheldrick, G.M. SHELXT - Integrated space-group and crystal-structure determination. *Acta Cryst. Sect. A*: Foundations and Advances **2015**, *71*, 3–8. DOI: 10.1107/S2053273314026370.

[5] Sheldrick, G. M. Crystal structure refinement with SHELXL. *Acta Crystallogr. Sect. C: Struct. Chem.* **2015**, *71*, 3–8. DOI: 10.1107/S2053229614024218.

[6] Spek, A. L. Structure validation in chemical crystallography. *Acta Crystallogr. Sect. D: Biol. Crystallogr*. **2009**, *65*, 148–155. DOI: 10.1107/S090744490804362X.

[7] C. R. Groom, I. J. Bruno, M. P. Lightfoot and S. C. Ward, The Cambridge Structural Database. *Acta Cryst*. **2016**. *B72*, 171–179. DOI: 10.1107/S2052520616003954.
